# Ancestral Genome Estimation Reveals the History of Ecological Diversification in *Agrobacterium*

**DOI:** 10.1093/gbe/evx255

**Published:** 2017-12-06

**Authors:** Florent Lassalle, Rémi Planel, Simon Penel, David Chapulliot, Valérie Barbe, Audrey Dubost, Alexandra Calteau, David Vallenet, Damien Mornico, Thomas Bigot, Laurent Guéguen, Ludovic Vial, Daniel Muller, Vincent Daubin, Xavier Nesme

**Affiliations:** Ecologie Microbienne, CNRS, INRA, VetAgro Sup, UCBL, Université de Lyon, Villeurbanne, France; Biométrie et Biologie Evolutive, CNRS, UCBL, Université de Lyon, Villeurbanne, France; Ecole Normale Supérieure de Lyon, Lyon, France; Commissariat à l’Energie Atomique et aux Energies Alternatives (CEA) Direction de la Recherche Fondamentale, Institut de Biologie Francois-Jacob (IBFJ), Genoscope, Evry, France; Laboratoire d’Analyse Bioinformatiques pour la Génomique et le Métabolisme, CNRS, UMR 8030, Evry, France; UEVE, Université d’Evry Val d’Essonne, France

**Keywords:** reverse ecology, ancestral genome, HGT, tree reconciliation, cotransferred genes, *Agrobacterium tumefaciens*

## Abstract

Horizontal gene transfer (HGT) is considered as a major source of innovation in bacteria, and as such is expected to drive adaptation to new ecological niches. However, among the many genes acquired through HGT along the diversification history of genomes, only a fraction may have actively contributed to sustained ecological adaptation. We used a phylogenetic approach accounting for the transfer of genes (or groups of genes) to estimate the history of genomes in *Agrobacterium* biovar 1, a diverse group of soil and plant-dwelling bacterial species. We identified clade-specific blocks of cotransferred genes encoding coherent biochemical pathways that may have contributed to the evolutionary success of key *Agrobacterium* clades. This pattern of gene coevolution rejects a neutral model of transfer, in which neighboring genes would be transferred independently of their function and rather suggests purifying selection on collectively coded acquired pathways. The acquisition of these synapomorphic blocks of cofunctioning genes probably drove the ecological diversification of *Agrobacterium* and defined features of ancestral ecological niches, which consistently hint at a strong selective role of host plant rhizospheres.

## Introduction

Our understanding of bacterial ecology is fragmentary. We usually know a subset of the environments from which species can be sampled, a few laboratory conditions in which they can be grown, and sometimes the type of interactions they establish with other organisms. Their genomes, believed to encode all the information that make their lifestyle possible, are now available. However, even if we succeeded in describing the molecular function of each single base in a genome, we would not necessarily know whether this function is significant in the prevalent environment of the organism (Doolittle 2013). In order to discover those functions that are ecologically relevant, an approach consists in recognizing traces of selection for functional adaptation in the histories of genomes. Comparing genomes reveals historical signals that can be used to retrace genome evolution, by estimating their hypothetical ancestral state and the course of the evolutionary events that shaped them over time. Using models of null expectation under neutral evolution, we can discern the decisive events in the adaptive evolution of species.

Bacterial genomes are in constant flux, with genes gained and lost at rates that can exceed the nucleotide substitution rate (Lawrence and Ochman 1997). Recognition of this dynamics led to the concept of pangenome, that is, the set of all homologous gene families present in a group of related genomes. The pangenome is the sum of the core and accessory genomes, which, respectively, gather the genes shared by all genomes in the data set and those found in some genomes only. In *E. coli*, for example, the core genome is estimated to include 1,800 gene families, whereas the accessory genome has >80,000 gene families, with two random strains typically differing by a thousand (Touchon et al. 2009; Land et al. 2015). In a genome, accessory genes are regularly gained (notably by transfer) or lost, leaving patterns of presence in genomes that are inconsistent with the strain phylogeny (Young et al. 2016).

For a majority of accessory gene families, this process occurs so rapidly that they are effectively observed in a single genome, caught by the snapshot of genome sequencing. This suggests that they only have transient, if any, adaptive value for their bacterial host (Daubin et al. 2003). However, this constant input of genes also allows adaptive accessory genes to settle in genomes, and become part of the core genome of a lineage. Such “domestication” events amidst the rapid turnover of genome gene content constitute the most remarkable deviations from a neutral model in which all genes are equally likely gained and lost. Clade-specific conservation of a gene is thus suggestive of adaptation to a particular ecological niche (Lassalle et al. 2015).

In a previous study, we investigated the diversity of gene repertoires among strains of *Agrobacterium* biovar 1 (Lassalle et al. 2011). This taxon contains several bona fide yet unnamed “genomic” species, numbered G1 to G9 and G13 and collectively named “*Agrobacterium tumefaciens* species complex” (*At*) according to the proposal of Costechareyre et al. (2010). Genes specific to the species under focus—G8, for which we proposed the name *A. fabrum*—were usually physically clustered in the genome, and these clusters in turn gathered genes that encoded coherent biological functions (Lassalle et al. 2011). The conservation of cofunctioning genes in genomic clusters appears unlikely in the context of frequent gene turnover. This pattern could be a trace of purifying selection that led to retain the whole gene clusters, because the selected unit was the function collectively encoded by the constituent genes. However, it could also result from a neutral process of gene flow, whereby neighbor genes with related functions (e.g., operons) happen to be transferred together and are then slowly eroded. These hypotheses may however be distinguished by analyzing the historical record of evolutionary events that led to the clustering of cofunctioning genes.

Most genes have complex histories, marked by many events of gene duplication, loss and, especially in the case of micro-organisms, horizontal transfers. The set of events affecting each homologous gene family in the pangenome under scrutiny can be summarized into an evolutionary scenario that can be seen as the path of gene evolution within and across branches of the tree of species. Evolutionary scenarios can be inferred by comparing the phylogenetic history of genes to the phylogenetic history of species, and by reconciling their discordances through the explicit inference of duplication, transfer and loss events (Doyon et al. 2011; Scornavacca et al. 2012). This in turn makes it possible to deduce the incremental shaping of genome gene contents, from ancestral to contemporary genomes, and to try and deduce the functional and ecological consequences of these changes.

We used the Rhizobiaceae family as a model taxon, and more particularly focused on the *At* clade for which we gathered a data set of 22 strain genomes from ten different species, including 16 newly sequenced genomes. We designed a new phylogenetic pipeline for the estimation of ancestral genome gene contents that accounts for horizontal gene transfer and gene duplication. Applied to our data set, this approach estimated blocks of cotransferred and coduplicated genes, enabling us to test hypotheses on how cofunctioning gene clusters were formed. Then we compared the level of functional cooperation of genes within blocks of cotransferred clade-specific genes to the expectation under a neutral model of horizontal gene transfer where genes are randomly picked from the donor genome. This comparison showed that clade-specific genes were more functionally related than expected, supporting the hypothesis that domestication of at least some clade-specific genes resulted from ecological selection.

Our estimated pangenome history—from single gene trees with transfer and duplication events to blocks of coevolved genes and functional annotations—was compiled in an integrative database called Agrogenom, which can be visualized and queried through an interactive web interface accessible at http://phylariane.univ-lyon1.fr/db/agrogenom/3, last accessed December 7, 2017.

## Materials and Methods

### Bacterial Culture Experiments

Bacterial growth was analyzed in the presence of phenylacetate (5 mM) using a Microbiology Bioscreen C Reader (Labsystems, Finland) according to the manufacturer’s instructions. Precultures of *Agrobacterium* strains were grown overnight in AT medium supplemented with succinate and ammonium sulfate. They were inoculated at an optical density at 600 nm (OD_600_) of 0.05 in 200 µl AT medium supplemented with appropriate carbon and nitrogen sources in Bioscreen honeycomb 100-well sterile plates. Cultures were incubated in the dark at 28 °C for 3 days with moderate shaking. Growth measurements (OD_600_) were performed at 20-min intervals.

### Genome Sequencing and Assembly

Genomic DNAs of the 16 *At* strains (table 1) extracted with the phenol–chloroform method were used to prepare libraries with DNA sheared into 8-kb inserts (median size). Raw sequence data were then generated using 454 GS-FLX sequencer (Roche Applied Sciences, Basel, Switzerland) with a combination of single-read (SR) and mate-pair (MP) protocols that yielded coverage ranging from 6.5× to 11× and from 5× to 8×, respectively (supplementary table S1, Supplementary Material online). Genome sequences were then assembled with Newbler version 2.6 (Roche Applied Sciences, Basel, Switzerland), using 90% identity and 40-bp thresholds for alignment of reads into contigs and the “-scaffold” option to integrate duplicated contigs into the scaffold assembly. Virtual molecules (chromosomes and plasmids) gathering scaffolds were manually created on the basis of plasmid profiles obtained from Eckhart gels (data not shown) and minimizing rearrangements between closely related genomes by taking into account whole-genome alignments obtained with the NUCmer program from the MUMMER package version 3.0 (Kurtz et al. 2004). Genome sequences were then annotated with the MicroScope platform (Vallenet et al. 2013) and made available through the MaGe web interface (www.genoscope.cns.fr/agc/microscope; last accessed December 7, 2017) or the European Nucleotide Archive (http://www.ebi.ac.uk/ena/data/view/ <ACCESSION NUMBERS>; last accessed December 7, 2017, with accessions marked with an a in table 1).

**Table 1:** List of the 47 Rhizobiales Strains Used in This Study

Clade/Taxon	Strain Name	NCBI TaxID	EMBL Sequence Accession Number	Nb. of Genes
*Agrobacterium biovar 1*/*A. tumefaciens* species complex (*At*)				
*A. sp.* G1	H13-3	861208	CP002248-CP002250	5,345
	5A	1107544	AGVZ00000000	5,518
	CFBP 5771	1183421	LT009762-LT009764^a^	5,546
	S56	1183429	LN999991-LN999996^a^	5,627
	TT111	1183430	LT009714-LT009717^a^	5,856
*A. sp.* G2 (*A. pusense*)	CFBP 5494	1183436	LT009718-LT009722^a^	6,013
*A. sp.* G3	CFBP 6623	1183432	LT009723-LT009726^a^	5,378
*A. sp.* G4 (*A. radiobacter*)	B6	1183423	LT009758-LT009761^a^	5,875
	CFBP 5621	1183422	LT009727-LT009729^a^	5,330
	Kerr 14	1183424	LT009730-LT009734^a^	5,870
	CCNWGS0286	1082932	AGSM00000000	4,979
*A. sp.* G5	CFBP 6626	1183435	LT009735-LT009738^a^	5,332
	F2	1050720	AFSD00000000	5,321
*A. sp.* G6	NCPPB 925	1183431	LT009739-LT009744^a^	6,139
*A. sp.* G7	NCPPB 1641	1183425	LT009775-LT009778^a^	6,041
	RV3	1183426	LT009745-LT009747^a^	5,182
	Zutra 3/1	1183427	LT009748-LT009751^a^	5,685
*A. sp.* G8 (*A. fabrum*)	C58	176299	AE007869-AE007872	5,639
	ATCC 31749	82789	AECL00000000	5,535
	J-07	1183433	LT009752-LT009755^a^	5,592
*A. sp.* G9	Hayward 0363	1183434	LT009779-LT009780^a^	4,502
*A. sp.* G13	CFBP 6927	1183428	LT009756-LT009757^a^	4,993
*Allorhizobium*				
*Allorhizobium vitis*	S4	311402	CP000633-CP000639	5,389
*Rhizobium sp.*	PDO1-076	1125979	AHZC00000000	5,340
*Rhizobium*				
*R. rhizogenes*	K84	311403	CP000628-CP000632	6,684
*R. etli*	CIAT 652	491916	CP001074-CP001077	6,109
	CFN 42	347834	CP000133-CP000138, U80928	6,016
	CNPAF512	993047	AEYZ00000000	6,544
*R. leguminosarum* bv. *viciae*	3841	216596	AM236080-AM236086	7,263
*R. leguminosarum* bv. *trifolii*	WSM1325	395491	CP001622-CP001627	7,001
	WSM2304	395492	CP001191-CP001195	6,415
*Ensifer/Sinorhizobium*				
*E. meliloti*	1021	266834	AL591688, AE006469, AL591985	6,234
	BL225C	698936	CP002740-CP002742	6,354
	CCNWSX0020	1107881	AGVV01000000	6,844
	AK83	693982	CP002781-CP002785	6,510
	SM11	707241	CP001830-CP001832	7,093
*E. medicae*	WSM419	366394	CP000738-CP000741	6,213
*E. fredii*	HH103	1117943	HE616890-HE616899	6,787
	NGR234	394	CP000874, CP001389, U00090	6,366
*Mesorhizobium/Chelativorans*				
*M. alhagi*	CCNWXJ12-2	1107882	AHAM00000000	7,184
*M. amorphae*	CCNWGS0123	1082933	AGSN00000000	7,075
*M. australicum*	WSM2073	7540353	AGIX00000000	5,934
*M. ciceri* bv. *biserrulae*	WSM1271	765698	CP002447, CP002448	6,264
*M. opportunistum*	WSM2075	536019	CP002279	6,508
*M. loti*	MAFF303099	266835	AP003017, BA000012, BA000013	7,281
*Chelativorans sp.*	BNC1	266779	CP000389-CP000392	4,543
*Parvibaculum*				
*P. lavamentivorans*	DS-1	402881	CP000774	3,636

a Accessions of strain genomes newly sequenced in this study.

### Genomic Sequence Data Set

The study focused on the *Agrobacterium* biovar 1 species complex a.k.a. *A. tumefaciens* (*At*) with an original data set of the aforementioned 16 new genomes, plus six publicly released ones (Goodner et al. 2001; Wood et al. 2001; Li et al. 2011; Ruffing et al. 2011; Wibberg et al. 2011; Hao, Lin, et al. 2012; Hao, Xie, et al. 2012). These 22 genomes covered ten closely related but genomically differentiated species (G1 to G9 and G13), with up to five isolates per species. The data set also included all Rhizobiaceae genome publicly available at the time of the database construction (spring 2012), and a few more distant relatives from the Phyllobacteriaceae and Rhodobiaceae families (table 1 and supplementary fig. S1, Supplementary Material online).

### Homologous Gene Family Database

Based the 47 complete genome sequence data set, we built a database of homologous gene families following the model of Hogenom databases (Penel et al. 2009). All annotated protein coding sequences (CDSs) were extracted and translated into protein sequences on which a all-versus-all pairwise BLASTP similarity search was performed to build a similarity network. Homologous gene families were derived from the connected components of the network using HiFix (Miele et al. 2012). Gene family sequences were then aligned at the protein level using MUSCLE (Edgar 2004) and reverse-translated into CDS alignments with pal2nal (Suyama et al. 2006).

### Reference Species Tree

To construct the reference species tree, we used 455 unicopy core gene families (i.e., families with exactly one copy per genome, listed supplementary table S2, Supplementary Material online), proceeding to 500 jackknife samples (draws without replacement) of 25 gene alignment sets, which were each concatenated and used to infer a maximum-likelihood (ML) tree using PhyML (Guindon and Gascuel 2003) using the same parameters as for gene trees (see supplementary text, Supplementary Material online). The reference phylogeny was obtained by making a consensus of this 500-tree sample with the CONSENSE algorithm from the Phylip package (Felsenstein 1993), and branch supports were derived from the frequency of the consensus tree bipartitions in the sample (supplementary fig. S2, Supplementary Material online). Alternative phylogenies were searched using the concatenate of the whole set of 455 universal unicopy families or from a concatenate of 49 ribosomal protein gene families (supplementary table S3, Supplementary Material online) to compute trees with RAxML (version 7.2.8, GTRCAT model, 50 discrete site-heterogeneity categories) (Stamatakis 2006).

### Reconciliation of Genome and Gene Tree Histories

We computed gene trees using PhyML (Guindon et al. 2003) for all 10,774 gene families containing at least three genes (supplementary text, Supplementary Material online) and estimated the branch support using the SH-like criterion. We rooted these gene trees using the combo criterion of TPMS (Bigot et al. 2013) so that, knowing the species phylogeny, both species multiplicity and taxonomic depth of all subtrees were minimized. A root minimizing these criteria favors reconciliation scenarios with less ancient gain (duplication and transfer) event, leading to scenarios more parsimonious in subsequent losses (supplementary fig. S3, step 1, Supplementary Material online). As this criterion yields poor results in the absence of ancestral duplications and the presence of many transfers, we used another method to root unicopy gene trees (i.e., trees of gene families with one gene per genome at most): we ran Prunier (Abby et al. 2010) for HGT detection (see below) and retained the root consistent with the most parsimonious transfer scenario.

We then inferred an evolutionary scenario for each gene family, that is, a mapping in the species tree of the presence/absence of gene lineages and of the events that led to their emergence. We reconciled the gene tree topologies with the species tree by annotating each of the 467,528 nodes found in the 10,774 gene trees with an estimated event of origination, duplication, transfer (ODT), or speciation. We used a bioinformatic pipeline that combines several methods dedicated to the recognition of different signals of duplication and horizontal transfers, fully detailed in the supplementary text, section 3, Supplementary Material online, and summarized below and in supplementary table S4, Supplementary Material online. In brief, gene trees were processed as follows: likely duplication events were first located by looking for clades with multiple gene copies per species (supplementary fig. S3, step 2, Supplementary Material online). Within the implied paralogous clades, subtree pruning and regrafting (SPR) moves that did not disturb branches with high (≥0.9) support were attempted, and retained as topology updates when they decreased the incidence of duplication events (by reducing the count of events or the count of descendant gene tree leaves). Another 17,569 nodes remained marked as putative duplications, out of which 28,343 potential paralogous lineages emerged. We used those as guide to extract subtrees in which every species was represented once, that is, unicopy subtrees. To deal with lineage-specific paralogues (“in-paralogues”), we extracted the several possible combinations of coorthologous gene copies (see Kristensen et al. 2011), producing unicopy subtrees with different but overlapping leaf sets (supplementary fig. S3, step 3, Supplementary Material online). Prunier, a parsimony-based method that takes into account the phylogenetic support of topological incongruences (Abby et al. 2010), was run on the unicopy subtrees to detect replacing transfer events based on significant topological conflict, that is, involving branches with statistical support >0.9 (supplementary fig. S3, step 3, Supplementary Material online). These reconciliations of potentially overlapping local subtrees yielded point estimate scenarios (involving a total of 22,322 phylogenetically supported transfer events), which were mapped back to the gene trees (supplementary fig. S3, step 4, Supplementary Material online). When several alternative (possibly conflicting) reconciliation scenarios were generated by independent inferences on overlapping lineage subtrees (“replicates”), the most likely scenario was chosen based on the number of similar events inferred in the neighboring gene families (supplementary fig. S3, step 5, Supplementary Material online), favoring the events involved in the largest block events (see the “Block event inference” section below).

In the next step, we completed the reconciliation of gene tree topologies with the species tree topology: topological incongruences may still have remained, notably involving gene tree branches with statistical support too low for Prunier to identify them as significant topological conflicts and to propose a transfer event. These topological incongruences needed to be explained—notwithstanding branch supports—by scenarios involving duplications or transfers (and subsequent losses), transfer scenarios being usually more parsimonious in the count of invoked events. We thus used the taxonomic incongruence algorithm from Bigot et al. (2013) to identify 1,899 conflicting branches as the places of additional transfer events, where otherwise 10,229 additional counts of duplication events would have been necessary (supplementary fig. S3, step 6, Supplementary Material online). This gave us a final estimate of the collection of duplication and horizontal transfer events leading to the emergence of new gene lineages. We then defined subfamilies of orthologues (nested in homologous gene families) as the descendants of every gene gain (ODT) event (supplementary fig. S3, step 6, Supplementary Material online). Finally, we used the Wagner parsimony algorithm implemented in the Count program (Csűrös 2008) to estimate scenarios of orthologous subfamily evolution, where transfers can be inferred to explain heterogeneous profiles of gene occurrence. This led to the annotation of 19,553 additional transfer events (supplementary fig. S3, step 7, Supplementary Material online). The illustrated description and programming details of the reconciliation pipeline used in this studies are available at: https://github.com/flass/agrogenom/blob/master/pipeline; last accessed December 7, 2017, and intermediary input/ouput files and data sets are available at: https://figshare.com/projects/Ancestral_genome_reconstruction_reveals_the_history_of_ecological_diversification_in_Agrobacterium/20894, last accessed December 7, 2017.

### Coordinates of Origination, Duplication and Transfer Events in the Species Tree

**Fig. 1.**
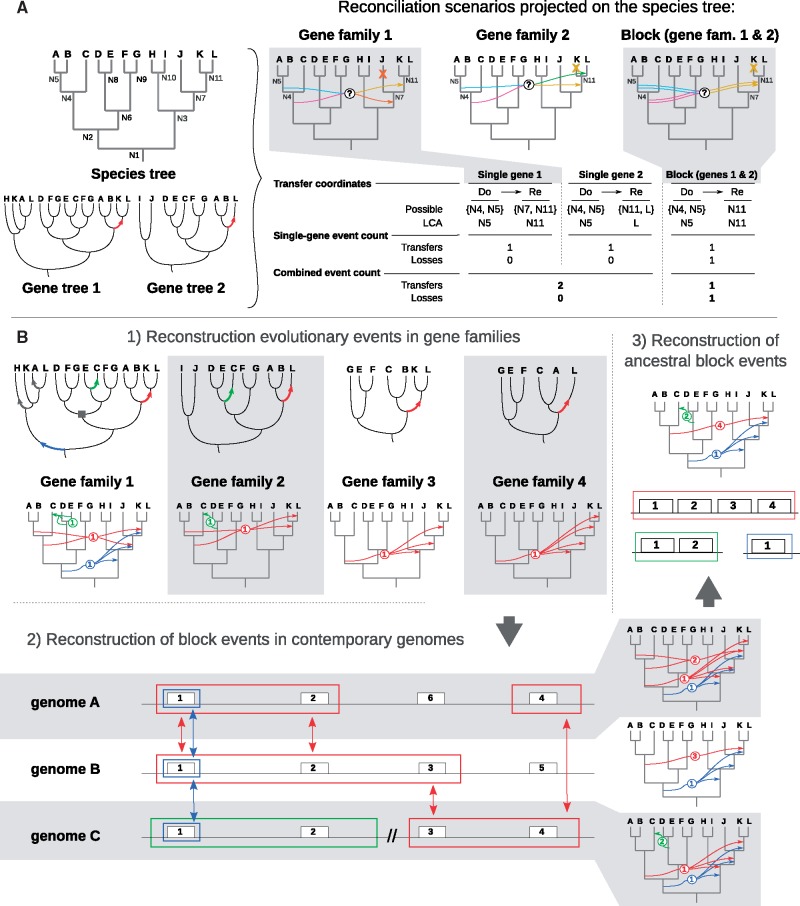
—Single gene versus block event reconciliation. (*A*) Transfers inferred in reconciled gene trees 1 and 2 can be translated into several possible scenarios in the species tree, and each scenario involves different donor (Do) and receiver (Re) pairs (multiple arrows with question marks, uncertain scenarios). If each gene family is reconciled separately, the scenarios that place the ancestral receiver as the last common ancestor of extant recipient genomes were chosen because they were the most parsimonious in losses (crosses mapped on the species tree and “Local event count” in inset table). In that way, the global scenario for the combined loci totalizes two transfers and no subsequent loss (inset table, “Combined event count”). If the transfer event coordinates are compatible (i.e., nonnull intersection: Re:{N7, N11} ∩ Re:{N11, L} = Re:{N11}) between gene families, we hypothesized the cotransfer of neighbor genes 1 and 2 as a common (Block) transfer event. By accounting for cotransfer events, a scenario was chosen which was not necessarily the most parsimonious one as regards losses for each gene. In this example, the most parsimonious global scenario for the combined loci totaled one block transfer and one subsequent gene loss. (*B*) Scheme of block event estimation. Origination, duplication and transfer events were first estimated separately in each gene family (1); for the sake of clarity, the example shows only transfer events, represented as arrows on gene tree branches (top) and between species tree branches (bottom). Compatible events affecting genes that were neighbor in at least one extant genome was aggregated into blocks (colored frames) (2) and this approach was then repeated across genomes (vertical double arrows) to estimate in which ancestral genomes the events occurred (3). Circled numbers indicate the number of genes combined into a same event.

Transfer events are characterized by the location of both donor and receiver ancestor nodes in the species tree (further referred to as “event coordinates”), which specifies the direction of the transfer; other gene gain events—gene origination or duplication—are only characterized by their location at an ancestral node in the species tree. The inference of coevents (events that involved several genes, see “Block event inference” below) relies on the detection of similar events across gene families, that is, events with the same coordinates. However, this can be challenging because independent evolution of gene families after a coevent may leave very different patterns in the respective gene trees, for instance due to different histories of gene loss after a common ancestral gain by cotransfer. When losses are considered, the right counts and locations of events are notoriously hard to estimate, as many combinations of loss events are possible for a fixed number of gain events, with little information—only gene absence, that is, missing data—to inform a choice. To get a point estimates of a scenario with gains and losses, one typically applies a criterion of parsimony on the count of loss events subsequent to a gain (e.g., by transfer), so the gain event is estimated to be located at the last common ancestor of the species represented in the recipient clade. Given that gene families often have different species representations, this can result in family-specific systematic biases when estimating event coordinates. Unmatched biases in coordinate estimates would strongly affect our ability to recognize that a same coevent affected neighbor gene families (fig. 1*A*). To reach a higher sensitivity in detecting similar events, we left counts and locations of loss events undetermined. This resulted in degrees of freedom on the ODT scenarios, with several connected branches of the species tree on which ODT events could possibly have happened (fig. 1*A*). As a result, we represented ODT event coordinates as sets of species tree nodes; two such sets are necessary in the case of transfers to characterize both donor and recipient locations (fig. 1*A*, inset table).

### Block Event Inference

We define block events as unique ODT events that involved a block of several contiguous genes in an ancestral genome (“ancestral block event”); by extension, “leaf block events” refer to the blocks of genes descended from such an ancestral block event, which typically form syntenic blocks in extant genomes and share a similar evolutionary pattern. We used a greedy accretion procedure that 1) linked matching events from neighbor gene families together into leaf block events, and 2) linked all homologous leaf block events to a common ancestral block event (fig. 1*B*). The complete algorithm for block event inference is described in the supplementary text, section 4, Supplementary Material online, and summarized below.

#### Leaf Block Event Inference

Using a greedy algorithm similar to that defined by Williams et al. (2012), we built leaf block events by iterative inclusion of events from contiguous gene families with compatible coordinates. For each replicon (chromosome or plasmid) in the database, we iterated over each gene following their position on the replicon; the nodes on the reconciled gene tree lineage leading to this gene were evaluated from tip to root. If a node was associated to an ODT event, we initiated a leaf block event containing this event as seed, and set the block coordinates as those of the seed ODT event. Then we looked for a similar event in the gene tree of the direct neighbor gene, using the same procedure to scan its lineage from tip to root. If the event associated to a node was of the same nature (O, D, or T) and with compatible coordinates (fig. 1*A*), it was appended to the leaf block event; the coordinate set of the leaf block event was then refined as the intersection of the coordinate sets of the block event and of the newly added event. When a matching event was found, this iterative search was repeated on the next neighbor gene’s lineage. In spite of finding such matching event, a leaf block event was extendable with a maximum of *g* “gap” genes (*g*_O_ = 1; *g*_D_ = 0; *g*_T_ = 4), and its elongation was terminated if no gene with a matching event was found beyond (supplementary fig. S4*A* and *B*, Supplementary Material online).

In the particular case of transfer (T) events, after the termination of a leaf block, inner gap genes were checked for phylogenetic compatibility of their gene tree with the scenario associated to the leaf block event (supplementary fig. S4*C*, Supplementary Material online): we checked that clades of donor and receptor species were not separated from each other in the gene tree by any strongly supported branches. When no branches or only branches with weak statistical support (<0.9) separated the clade pair, the transfer event hypothesis was not rejected and the leaf block event integrity was maintained. Conversely, when the gene tree of a gap gene carried a strong signal rejecting the transfer event, the original leaf block was split into two leaf blocks representing separate transfer events (supplementary fig. S4*D*, Supplementary Material online).

#### Ancestral Block Event Inference

Then, we estimated ancestral block events by searching homology relationships between leaf block events. Block homology was defined as the presence in each leaf blocks of at least one homologous gene associated to the same gene tree event (fig. 1*B*, step 2); this relationship can be found between leaf block events from different extant genomes or from a same genome. Ancestral block events were iteratively assembled from homologous leaf block events, and their coordinates were estimated by intersecting the coordinates of their members (fig. 1*B*, step 3).

This last step notably united certain leaf block events scattered in an individual genome. This allowed us to infer the unity of ancient gene blocks that were larger than their derived forms in extant genomes. Because of gene insertion/deletion or genomic rearrangement, contiguity of genes descending from a same coevent could easily have been disrupted. Due to this mutational process, the gene content of putative homologous leaf block events could differ, and their estimated block event coordinates could differ too. The leaf block homology relationship is supposed to be transitive, but due to these potential differences, incompatibilities could arise during the iterative accretion of leaf block events into ancestral events; in that case a heuristic was used to resolve the conflict between putative homologous leaf block events and distribute them into a number of self-compatible ancestral blocks.

#### Detection of Block Events in Agrogenom Scenarios

Block events were investigated for origination (O), duplication (D), and transfer (T) events. We did not investigate losses (L), because random convergent losses occur at a higher rate (Kuo et al. 2009; David and Alm 2011; Szöllősi et al. 2012), and the larger solution space of loss scenarios leads to a higher risk of nonspecific aggregation of unrelated loss events. For a similar reason of a high risk of false positives, we did not investigate O and D block events on the deep, long branches of the species tree (supplementary fig. S1, Supplementary Material online: N1, N2, and N3, respectively, leading to *Parvibaculum lavamentivorans*, the *Mesorhizobium*/*Chelativorans* clade, and the Rhizobiaceae clade), where many events were annotated with undistinguishable coordinates that likely occurred separately over time (2,586 O events and 2,934 D events overlooked). After all homology search, the coordinates of the ancestral block events for O, D, and T were finally reduced to their most recent possible location in the species tree and subsequent losses were inferred accordingly to complete the gene evolution scenarios (point estimates for each gene family).

### Detection of Clade-Specific Genes from Phylogenetic Profiles

Clade-specific genes were defined as genes gained (or lost) by the clade ancestor and conserved (not regained) in all clade members since. We first identified genes marked by gain/loss events in the genome of a clade ancestor. Then, we identified clade-specific genes by searching for contrasting patterns in the phylogenetic profile of the presence or absence of each gained/lost gene. These profiles were established from the scenarios of orthologous subfamily evolution (see above and supplementary text, section 3, step 6, Supplementary Material online). A background clade was chosen as the one corresponding to the next higher taxonomic unit (genus, species complex, etc.) in which the focal (foreground) clade was nested. Contrast was initially defined between the foreground and background clades, where foreground genomes had a consistently opposite pattern to that of genomes in the background clade. However, possible subsequent transfer or loss events in the background clade can blur the contrasting pattern in phylogenetic profiles. Clade-specific genotypes were thus identified using a relaxed definition of clade specificity, that is, where the presence/absence contrast could be incomplete, with up to two genomes in the background clade sharing the foreground state.

### Functional Homogeneity of Gene Groups

To measure to which extent cotransferred genes showed coherence in the functions they encoded, we used metrics of semantic similarities of the Gene Ontology (GO) terms annotated to the gene products. First, we retrieved GO annotations from UniProt-GOA (http://www.ebi.ac.uk/GOA/downloads, last accessed December 7, 2017) (Dimmer et al. 2012) for public genomes, and used a similar pipeline of association of GO terms to gene products to annotate the genomic sequences produced for this study. The results of several automatic annotation methods were retrieved from the PkGDB database (Vallenet et al. 2013) based on similiraty searches: HMM profile searches on InterPro, HAMAP, and PRIAM databases and BLASTP searches on the SwissProt and TrEMBL databases (as of the February 5th, 2013), with a general cut-off *e*-value of 10*e*-10. GO annotations were then mapped to gene products using mappings between those method results and GO terms as provided by Uniprot-GOA for electronic annotation methods (http://www.ebi.ac.uk/GOA/ElectronicAnnotationMethods, last accessed December 7, 2017): InterPro2GO, HAMAP2GO, EC2GO, UniprotKeyword2GO, UniprotSubcellular_Location2GO. The annotation data set was limited to the electronically inferred data to avoid biases in the annotation of certain model strains or genes. The resulting functional annotations of proteomes were analyzed in the context of Gene Ontology term reference (full ontology file downloaded at http://www.geneontology.org/GO.downloads.ontology.shtml, last accessed December 7, 2017) (Ashburner et al. 2000). Functional homogeneity (*FH*) within a group of genes is defined as the average value of the pairwise functional similarities between all gene products in the group, each of which is the average value of pairwise similarities between all terms annotated to a pair of genes. Similarities were measured using the *Rel* (within a gene) metric and the *funSim* metric (between genes) (Schlicker et al. 2006; Pesquita et al. 2009). Computations were done using a custom Python package derived from AIGO package v0.1.0 (https://pypi.python.org/pypi/AIGO; last accessed December 7, 2017).

To assess if cotransfer of genes was associated with coherent functions, we compared the *FH* of cotransferred gene blocks to that of random groups of genes, obtained either by uniformly sampling (i.e., by random drawing without replacement) individual nonlinked genes or by sampling genomic windows of neighbor (linked) genes. *FH* values were computed for all windows of neighbor genes around a replicon, and a sample of the same size was drawn for random combinations of nonlinked genes. Because the size of the group of genes strongly impacts the computation of the similarity metrics, and because the annotation density can vary among organisms and replicons (contiguous DNA molecules), the distributions of *FH* values were calculated per replicon and per group size. Note that the set of blocks of cotransferred genes is included in the set of all genomic windows, but that we used nonoverlapping subsets for statistical comparisons.

To test if functional coherence of a block of cotransferred genes impacted its probability of retention after transfer, we compared the *FH* values of genes from two sets of ancestral block events: those where all consituent genes were conserved in all descendant leaf block events, and those where part of the genes were lost in at least one descendant leaf block events. To avoid biases linked to variation in age of transfer events, this comparison was made only for events that occurred in ancestors of species-level clades of *At.*

### Agrogenom Database

All data about genes (functional annotations and gene families), genomes (position of genes, architecture in replicons …), the species tree (nodes and taxonomic information), reconciliations (gene trees and ODT events), block events, inference analyses (parameters, scores …), and all other data relative to the present work were compiled in a PostgreSQL relational database called Agrogenom. The database schema, input data and build procedure are available at https://github.com/flass/agrogenom/tree/master/pipeline/database; last accessed December 7, 2017; its content is browsable through a web interface at http://phylariane.univ-lyon1.fr/db/agrogenom/3/, last accessed December 7, 2017.

## Results and Discussion

### Genomic Data Set and Reference Species Tree

To explore the genomic diversity of the Rhizobiaceae pangenome, we gatherred 47 genomes from the *Agrobacterium*, *Rhizobium*, *Sinorhizobium*/*Ensifer*, *Mesorhizobium*/*Chelativorans*, and *Parvibaculum* genera into the Agrogenom database. These genomes contain 281,223 coding sequences (CDSs, or genes hereafter) clustered into 42,239 homologous gene families. Out of these families, 27,547 were singletons with no detectable homologues (ORFan families) and 455 were found in exactly one copy in all 47 genomes (unicopy core gene families). Following the procedure used in Abby et al. (2012), a species phylogeny was inferred from the concatenation of unicopy core gene family alignments, using jackknife resampling of genes to compute branch supports (supplementary fig. S2, Supplementary Material online). Significant support was obtained for all clades corresponding to previously described species: *S. melitoti, R. etli, R. leguminosarum*, and in particular *At* species G1, G8, G4, G5, and G7. In contrast, branch support was low for the relative positioning of most strains within species, showing conflicting (or a lack of) signal among concatenated genes. Within the *At* clade, higher-order groupings were also highly supported: G8 with G6 (hereafter named [G6–G8] clade), G5 with G13, ([G5–G13] clade), G1 with [G5–G13] ([G1–G5–G13] clade), G3 with [G1–G5–G13] ([G3–G1–G5–G13] clade), G7 with G9 ([G7–G9] clade), and G4 with [G7–G9] ([G4–G7–G9] clade). Only a few deep splits such as the position of species G2 and [G6–G8] clade relatively to the *At* root were poorly supported (supplementary fig. S2, Supplementary Material online). We compared this species tree topology to two others obtained with alternative data sets (see Materials and Methods): all three methods yielded very similar results concerning the placement of the different genera and species (supplementary fig. S5, Supplementary Material online); the main difference resided in the rooting of *At* within the Rhizobiaceae clade, and the placement of lone representatives for species G2 and G3. Investigation of the pangenome-wide support for alternative hypotheses (see supplementary text, section 1 and supplementary fig. S6, Supplementary Material online) confirmed that the best topology was provided by the jackknife sample consensus tree presented supplementary figure S2, Supplementary Material online. A phylogeny estimated from the genome gene contents proved less appropriate to discriminate species, indicating the occurrence of a large quantity of HGTs (supplementary fig. S7, Supplementary Material online).

### Reconciliation of Gene and Species Histories

To estimate the history of HGT and other macro-evolutionary events that shaped the Rhizobiaceae pangenome, we reconciled the topologies of gene trees with the species tree, that is, we explained their incongruence by assigning events of origination, duplication, transfer (ODT), or speciation to the gene tree nodes. We used a succession of heuristics for the reconciliation of gene and species trees aimed at solutions parsimonious in losses and transfers (supplementary fig. S3, Supplementary Material online). The combination of events estimated in each gene tree resulted in an estimated scenario of evolution of the gene family along the species tree.

Out of the 467,528 nodes found in the rooted gene trees of the 10,774 families that contained at least three genes, our pipeline assigned a total of 7,340 duplication events (1.5% of all gene tree nodes) and 43,233 transfers (9.2%). The remainder of unannotated gene tree nodes corresponded to speciation events (where gene tree topologies locally follow the species tree) and originations (emergence of the gene family in our data set, mapped at the root of the gene tree) (table 2). Based on the estimated ancestral genome gene contents, we distinguished additive transfers that brought new genes, as opposed to those that replaced current orthologous genes. Replacing transfers accounted for a quarter of total transfers (9,271 events). Additive transfers contribute almost five times more than duplications to the total gene input in genomes (table 2), showing that transfer is the main source of gene content innovation in *At*.

**Table 2:** Origination, Duplication, Transfer, and Speciation Events Estimated in Reconciliations of the Agrogenom Database

Event Type	Single Gene Events	Block Events	(of Size >1)	Difference After Event Integration into Blocks
Originations	5,189	4,267	(667)	−922
Duplications	7,340	5,819	(778)	−1,521
Total transfers	43,233	32,255	(5,649)	−10,978
Replacing transfers^a^	9,271	—	—	—
Additive transfers^a^	33,962	—	—	—
Total ODT	55,762	42,341	(7, 094)	−13,421
Implied losses	29,843	32,739	—	+2,896
Total ODTL	85,605	75,080	—	−10,525

O, origination; D, duplication; T, transfer; L, loss; ODT refers to the combination of all O, D, and T events, while ODTL also includes losses.

a Replacing and additive transfers were not distinguished in block events.

### Identification of Coevents Involving Neighbor Genes Leads to a More Parsimonious Genome-Wide Scenario

Large-scale comparative genomics analyses revealed that insertions in genomes are typically composed of several consecutive genes, indicating that blocks of genes can evolve in linkage across genomes (Vallenet et al. 2009). Yet, to date, gene evolution scenarios have generally been evaluated for each gene tree independently of its neighbors (Makarova et al. 2006; Kettler et al. 2007). This is questionable because a scenario may be optimal (e.g., more parsimonious) for a given gene, but suboptimal in a model where genes can be part of the same event (fig. 1). We developed a procedure to identify blocks of genes that likely coevolved through the same event, based on the compatibility of their coordinates in the species tree (see Materials and Methods).

By assembling compatible ODT events from individual reconcilations of neighbor genes, we inferred putative “block events”, that is, unique evolutionary events that involved blocks of neighbor genes (fig. 1*B* and *C*). At the pangenome scale, we found numerous such block events in *At* genomes, with 17.5% of transfers and 13.3% of duplications involving at least two genes (table 2). Several thousands of transfer events were infered to involve 2–6 genes, and a few hundreds to span a dozen or more consecutive genes in extant genomes (supplementary fig. S8*A*, Supplementary Material online). Moreover, blocks of ancestral genes that we estimated to have been transferred among ancestral genomes (“ancestral block events”) often appeared as larger units than their extant counterparts (supplementary fig. S8*B*, Supplementary Material online), indicating that rearrangements and partial losses in descendant genomes frequently dismantled the syntenic blocks involved in ancient transfers.

As many groups of ODT events that individually appeared as convergent were factorized into unique coevents, the relative frequency of event types that were estimated dramatically changed: relatively to scenarios inferred using a parsimony criterion (minimization of losses) independently applied to single gene histories, block event scenarios resulted in a decrease of 13,421 ODT events, most of them transfer (T) events (10,978, −25.4%), and an increase of loss (L) events (2,896, +9.7%) (table 2). However, the count of additional losses was certainly overestimated, because block events of gene loss are bound to have occurred, but we did not intend to factorize loss events in this study (see Materials and Methods).

This difference in the estimated number of gene losses was due to the frequent underestimation of the event age when considering only scenarios for individual gene families, relatively to joint scenarios for several gene families. Indeed, the loss scenarios were generally estimated by fixing the timing of the preceding gene gain (O, D, or T) events to their most recent possible location—the most parsimonious solution with respect to losses. In the case of block event scenarios, ODT events were dated to the most recent *common* location of all single-gene event parts, which by definition must be equally ancient as, or more ancient than the single-gene estimates. This resulted in globally older ancestor for block gain events, with a higher number of lineages between the ancestor and extant representatives in which to invoke subsequent losses (fig. 1*A*). ODT events are thought to be less frequent than gene loss (L) events, and the more complex pattern of transfers (characterized by a donor and a recipient) makes it less likely for T events with similar coordinates to have occurred convergently in neighbor genes in the absence of a linkage hypothesis. As a consequence, factorizing similar ODT events for neighbor genes appears a to be a suitable approach to obtain a pangenome-wide scenario that is much more parsimonious in the total number of all kinds of events, that is, ODTL events.

### Inferred Genome Histories Suggest Selection for New Genes in Ancestors of Key *At* Lineages

**Fig. 2.**
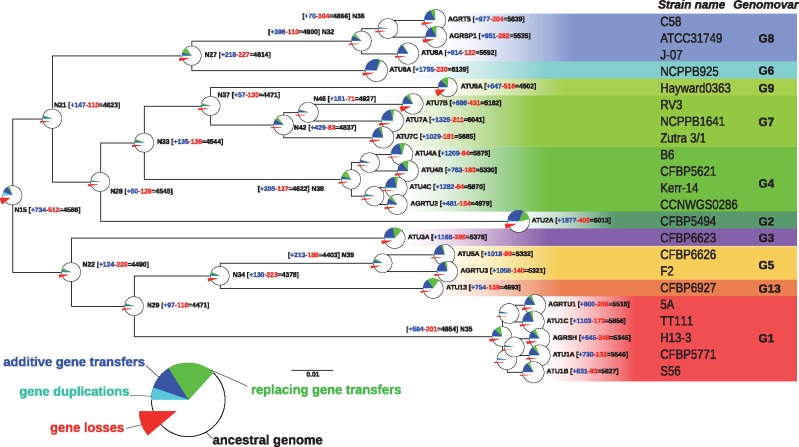
—Ancestral genome sizes and gain/loss events. The tree is a subtree of that presented in supplementary figure S1, Supplementary Material online, and focuses on the *At* clade. Net gains (+) and losses (−) and resulting genome sizes (=) are indicated next to nodes. Disc at inner and terminal nodes represent estimated ancestral genomes and extant genomes, respectively; surfaces are proportional to genome sizes. Prevalence of events shaping the gene content are indicated by pie charts indicating the fraction of losses (red), gains by duplication (cyan), gains by transfer (blue), and gene conversions/allelic replacements (green). The relatively high number of event occurring at the *At* root is related to the long branch from which it stems in the complete Rhizobiales tree (supplementary fig. S1, Supplementary Material online), which is not represented here.

Our inferred history of gain and loss in ancestral genomes of *At* showed heterogeneous dynamics across the species tree. First, the estimated genome sizes were significantly lower in estimated ancestral genomes than in extant genomes (fig. 2 and supplementary table S5, Supplementary Material online). For instance, the estimated genome gene content, or gene repertoire, of the *At* clade ancestor contained around 4,500 genes, whereas extant genomes had an average size of 5,500 genes. This 1,000-gene difference approximately corresponds to the number of genes recently gained along the terminal branches of the species tree (fig. 2), indicating a divide in contemporary genomes between a long-standing gene repertoire and a large fraction of newly acquired genes still segregating in the population. Our ancestral genome estimation procedure did not estimate the count of unobserved ancient genes; however, a similar-size polymorphic gene repertoire probably existed in the *At* clade ancestors and was mostly lost in all sampled descendants.

**Fig. 3.**
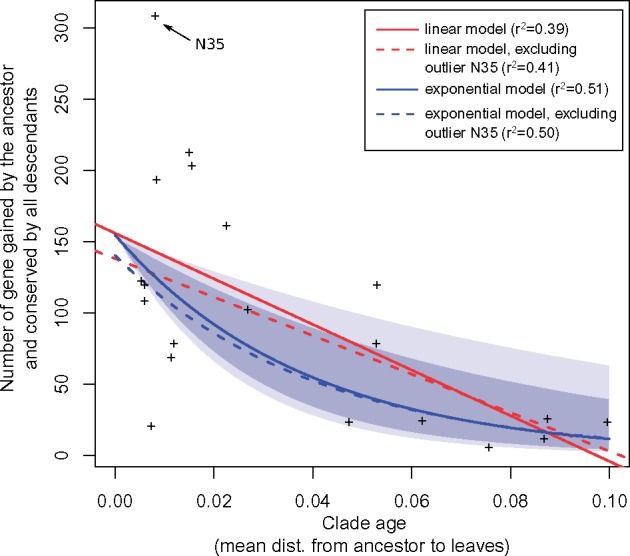
—Retention of gained genes within *At* genomes follows a survival model. Node “N15” (the G1 species ancestor) is the strongest driver in the linear regression. Dark and light shaded areas represent the 95% and 99% confidence intervals of the exponential model, respectively (solid blue line).

The length of the branch leading to the ancestor best explained the number of genes gained and lost by an ancestor (linear regression, *r*^2^ = 0.59 and 0.32 for gains and losses, respectively), although removing the extreme point of node N35 (the G1 species ancestor) sharply decreased the correlation coefficients (*r*^2^ = 0.27 and 0.28) (supplementary fig. S9*A* and *B*, Supplementary Material online). Interestingly, the number of genes gained by an ancestor and subsequently conserved in all members of the descendant clade, that is, clade-specific genes, was robustly explained by the ancestor age (*r*^2^ = 0.39, or 0.41 when removing N35) (supplementary fig. S9*F*, Supplementary Material online). This relationship was better described by a decreasing exponential regression (*r*^2^ = 0.51, or 0.50 when removing N35), which reflected a process of “gene survival” in genomes over time (fig. 3). Alternatively, these trends may have resulted from a systematic bias in our estimation procedure: for instance, because our block event inference algorithm tended to place gene gains higher in the species tree than an inference considering a gene family alone would have done (fig. 1*A*), subsequent losses may have been inferred too frequently in early ancestors, generating this pattern of decay over time; however similar trends were observed for scenarios without block agregation (data not shown).

We identified outlier genomes in this putative “gene survival” process, as the nodes with the largest residuals in the exponential regression (out of the 95% confidence interval). They were, in a decreasing order of excess of conservation relative to their age, the ancestors of the [G6–G8], G1, G5, [G5–G13], G8 clades and those of subclades of G4 and G7 (supplementary figs. S9*F* and S10, Supplementary Material online). These excesses of conservation did not systematically reflect a particular excess of gains in the ancestors: ancestors of G1 and G8 (nodes N35 and N32) did indeed gain more genes than predicted by their respective branch lengths, whereas ancestors of [G6–G8], [G5–G13] and G5 (nodes N27, N34, and N39, respectively) rather lost genes in excess (supplementary fig. S9*C* and *D*, Supplementary Material online). In the latter cases, excess conserved gains may thus have stemmed from a fixation bias like natural selection for new genes. The outliers that fell above this trend—those clades that conserved more genes than predicted by their age—all belonged to [G1–G5–G13] and [G6–G8] clades (supplementary fig. S10, Supplementary Material online). The higher rate of conservation in these clades suggests a higher proportion of genes under purifying selection since their ancestral acquisitions, that is, domesticated genes.

Clade-specific genes conserved for a long time likely provide a strong adaptive feature to their host organism. A new adaptive trait can improve an organism’s fitness by increasing the differentiation of its ecological niche relatively to cognate species, and thus enable it to escape competition. This emergence of a new ecotype—an ecologically differentiated lineage—can for instance occur through a gain of function (e.g., via additive HGT) that allows for exclusive consumption of a resource (Lassalle et al. 2015) or the change in relative reliance on a set of resources (Kopac et al. 2014). The spread of such niche-specifying traits to close relatives of the ecotype should be counter-selected (Cohan and Koeppel 2008), so that their occurrence is expected to be restricted to the descendants of the ecotype, that is, to be clade-specific. Identifying such adaptive traits among clade-specific genes is thus the key to the understanding of the unique ecological properties of a bacterial clade.

### Clusters of Clade-Specific Genes Are Under Purifying Selection for Their Collective Function

Niche-specifying traits are expected to provide higher differential fitness if they are less likely to be already present in, or independently acquired by, competing relatives. Hence, the best candidates for niche-specifying traits consist of novel and complex traits relying on an array of biochemical functions coded by separate evolving units (genes) and do not depend on preexisting pathways, making it unlikely to occur several times by chance. In such a case, it is crucial for the complete set of underlying biochemical functions to be gained at once for it to provide any kind of advantage. Such an event can typically happen with the cotransfer of a complete operon. In a previous study focused on G8 genomes (Lassalle et al. 2011), we observed that clade-specific genes tended to occur in clusters of genes with related biochemical function. This apparently nonrandom pattern of gene conservation suggests that cotransferred groups of genes collectively coding for a function were selected among incoming transferred genes: initially by positive selection for their new function upon trnasfer reception, and later on by negative (purifying) selection against the destruction of the group by rearrangement or partial deletion. This led us to consider clusters of cofunctioning clade-specific genes as good candidates for niche-specifying determinants (Lassalle et al. 2011).

Yet, it is well known that bacterial genomes are organized in functional units such as operons, super-operons, etc. (Rocha 2008), and the cotransfer of cooperating genes could neutrally result from the functional structure of the donor genomes. However, the transferred DNA segments are most probably taken randomly from donor genomes, apart from the special case of genes encoding their own mobility. Thus, under a neutral model, cotransferred genes should not always be cofunctioning, and the probability for a transferred fragment to span a functional element like an operon is expected to be close to that of any similarly sized fragment of the donor genome.

**Fig. 4.**
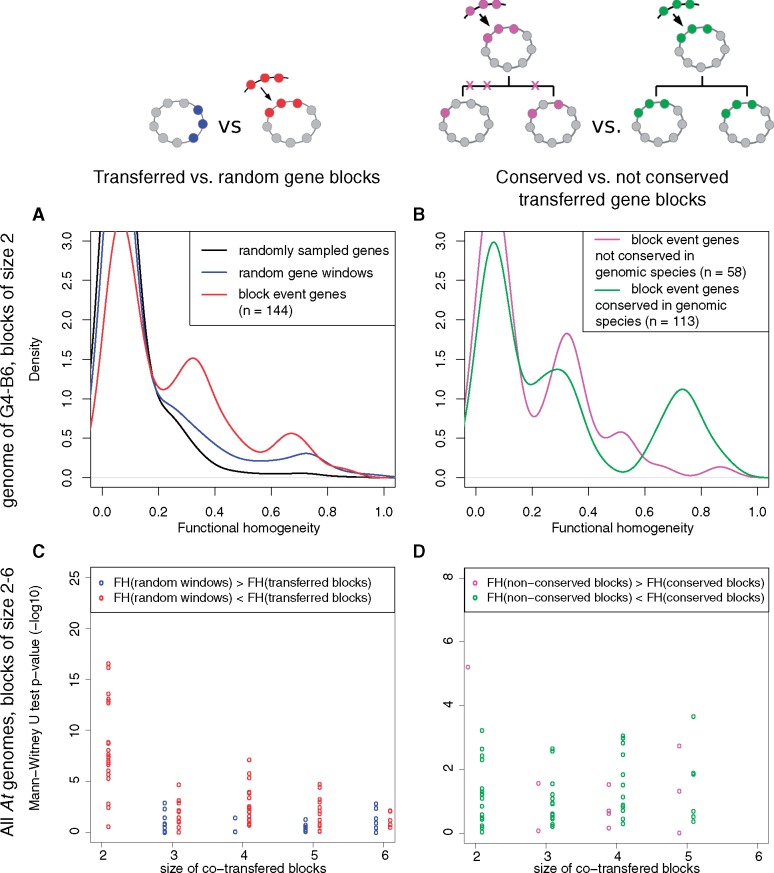
—Functional homogeneity of gene clusters. (*A*, *B*) Distribution of functional homogeneity (*FH*) values of genes within clusters using representative plots comparing clusters of two genes in the B6 genome (a G4 member). (*A*) Comparison of *FH* values of groups of two genes taken from the B6 genome: randomly distant pairs (black), any pair of neighbor genes without a common transfer history (blue), or pairs of cotransferred neighbor genes (red). (*B*) Comparison of *FH* values of pairs of cotransferred genes from families conserved across all G4 strains (green) or not conserved (red). (*C*, *D*) Distribution of *P* values of Mann–Whitney–Wilcoxon sum of ranks test comparing the distributions of *FH values* (made independently for all *At* genomes at all discrete block sizes) of (*C*) random windows of non cotransferred genes versus blocks of cotransferred genes or (*D*) conserved versus nonconserved blocks of cotransferred genes. Each point represents an observation from an extant *At* genome for a given gene group size (on the *x*-axis). Point colors indicate the higher-*FH* category: (*C*) blue, *FH*(random windows) > *FH*(transferred blocks), 29/95 tests (4/49 significant tests); red, *FH*(random windows) < *FH*(transferred blocks), 66/95 (45/49); (*D*) purple, *FH*(nonconserved blocks) > *FH*(conserved blocks), 11/60 (2/13); green, *FH*(nonconserved blocks) < *FH*(conserved blocks), 49/60 (11/13). Tests were considered significant at *P* < 0.01.

To test whether clustering of functionally related clade-specific genes resulted from natural selection, we designed tests that assessed the relationship between gene transfer history and functional homogeneity (*FH*) (see Materials and Methods). First, we verified that random groups made of physically distant genes had lower *FH* values than groups of neighbor genes, confirming that *FH* captures the functional structure of a genome (fig. 4*A*).

Then we compared random groups of neighbor genes without a shared transfer history to blocks of cotransferred genes of the same size. The distribution of *FH* values showed that while blocks of cotransferred genes generally gathered genes that do not encode related functions or for which functional annotations are insufficient (FH ∼ 0), a minor fraction presented intermediate to high functional relatedness (e.g., in the G4–B6 genome, minor modes at *FH* ∼ 0.35 and *FH* ∼ 0.75, fig. 4*A*). Blocks of co-transferred genes had significantly higher *FH* values than random groups in 45 out of 49 significant tests performed on independent combinations of genomes and block sizes (fig. 4*A* and *C*). This shows that fixation of transferred blocks of genes in genomes was biased towards blocks that code for functional partners in a biological process. This observation supports the hypothesis of positive selection favouring fixation in a recipient genome of the transferred genes that can immediately provide a selectable function. It is also compatible with the “selfish operon” model proposed by Lawrence and Roth (1996): in host genomes, transfer followed by selection for readily functional multi-genic traits is thought to lead to the prevalence of genes clustered into tightly linked functional units.

In addition, among the groups of genes acquired by transfer, those that were conserved in all descendants of the recipient ancestors had more coherent annotated functions than the nonconserved ones (11/13 significant tests are positive, fig. 4*B* and *D*). The hypothesis of conserved cotransferred genes encoding more related functions than nonconserved ones was previously proposed based on manual inspection of the functional relatedness of a few transferred operons in *E. coli* (Homma et al. 2007) or the metabolic flux coupling of spatially clustered transferred genes (from possibly mixed origins) in Gammaproteobacteria (Dilthey and Lercher 2015). The present study presents a first quantitative estimation of functional relatedness within blocks of cotransferred genes, and provides a statistical argument for purifying selection enforcing their collective conservation in genomes. This supports our initial hypothesis that clusters of clade-specific genes participating to a same pathway were more likely to carry sufficient information to encode a new adaptive trait, and had been under continued selection since their acquisition. It follows that the adaptations that characterize the ecological niche of a clade should be revealed by identifying of the genes specifically conserved inside a clade, and notably those grouped in clusters with related functions.

### Identification of Clade-Specific Genes in *A. tumefaciens* Key Clades

**Fig. 5.**
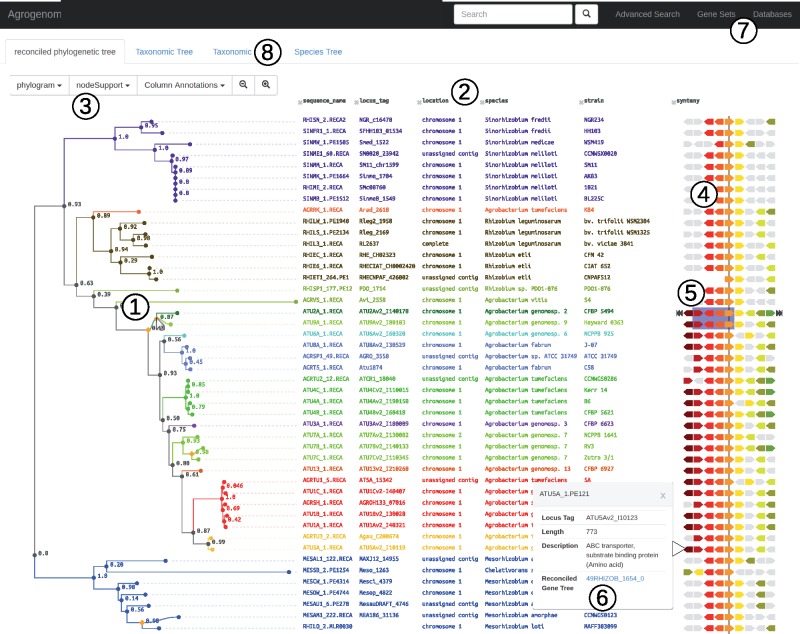
—Snapshot of the Agrogenom web interface. View of the *recA* gene family. 1) Reconciled gene tree; the orange diamond under the mouse cursor indicates a transfer event from G2-CFBP 5494 to G9-Hayward 0363. 2) Detailed annotation of the sequences at the tip of the tree, including locus tag (linking out to MaGe genome browser), chromosomal location, taxon name, database cross-references, etc. 3) Dynamic menu to adapt the level of displayed information. 4) Syntenic view in the genomic neighborhoods of the focal gene family; homologues share the same color, defined with reference to a chosen sequence (indicated by the navigation arrows on the sides). 5) The blue frame indicates a block transfer event involving four gene families; this block appears dynamically when hovering the cursor above the transfer node in the gene tree. 6) A pop-up window with the functional annotation and characteristics of a gene can be generated by double-clicking on the gene; it contains the link to the gene tree of the gene family. 7) Search menus: rapid search using gene names; “Advanced search” to reach a gene family from its various annotation fields; “Gene Sets” to browse lists of genes: clade-specific genes, core genome, ancestral gene content, clade-specific gains/losses. 8) Alternative views: the reference species tree and a projection of the gene family distribution among taxa.

We investigated the histories of gene gain and loss in the clades of *At* to identify the synapomorphic presence/absence of genes in these clades. We used an automated method that recognizes profiles of contrasted gene occurrence among sister clades by spotting ancestral gene gains or losses that resulted in their conserved presence or absence in the descendant clade (see Materials and Methods). Doing so, we accounted for convergent gains/losses of orthologous genes in distant clades, notably in cases of a transfer from one clade ancestor to another; this allowed us to evidence the specific sharing of genes between nonsister species of *At*. Listings of clade-specific genes of those key *At* clades can be found in Data Set S1, Supplementary Material online, or can be browsed on the Agrogenom database website http://phylariane.univ-lyon1.fr/db/agrogenom/3/; last accessed December 7, 2017 (fig. 5). Generally speaking, clade-specific genes were often located in relatively large clusters encoding coherent biochemical functions or pathways, which are summarized in supplementary table S6, Supplementary Material online and hereafter numbered with the AtSp prefix. Those clade-specific gene clusters often matched transfer or origination block events as estimated above (supplementary Data Set S1, Supplementary Material online), although often with limited coverage or with several transfer blocks mapping to a single clade-specific cluster. This suggests that block gain events are likely to cluster at the same loci. Alternatively, it suggestes a limitation of our search procedure in the face of the complexity of gene histories, with different patterns of multiple consecutive transfers in different gene families preventing recognition of their common history. Extended description of the noteworthy biochemical functions encoded in these clade-specific gene repertoires can be found in the supplementary text, section 6, Supplementary Material online. Species G1, G8, G4, and G7, were represented by several closely related extant genomes, and therefore were particularly amenable for the accurate definition of clade-specific gene repertoires. For these species, chromosomal maps (supplementary figs. S11, S12, S13, and S14, Supplementary Material online) show that species-specific genes were unevenly located on the various replicons of *At* genomes, with a bias towards accumulation on the linear chromosome (Lc), and an unexpected presence on the At plasmid (pAt) (supplementary tables S6 and S7, Supplementary Material online).

### Secondary Replicons of Agrobacterium Genomes Bear Clade-Specific Innovations

Rhizobiaceae have complex genomic architectures composed of a primary chromosome, plus a secondary chromosome or megaplasmid bearing essential genes, called the chromid (Harrison et al. 2010), and a variable complement of plasmids of various sizes (Young et al. 2006). More specifically, the chromid of the *Agrobacterium* genus (Mousavi et al. 2015; Ormeño-Orrillo et al. 2015), which includes the *At* clade, is linear (Slater et al. 2009, 2013) as a result of a unique ancestral event of linearization and thus constitutes a synapomorphy of this clade (Ramírez-Bahena et al. 2014). Another general feature of *At* genomes is the frequent presence of a pAt, a megaplasmid that was long referred to as the cryptic plasmid because its role in agrobacterial cell biology remains largely unknown. We found that different pAt types were restricted to certain genomic backgrounds (based on their replication gene phylogenies) and carried clade-specific gene clusters at the species level (in G1, G8, G4, and G7 species) or higher (in [G6–G8] clade) (supplementary figs. S11, S12, S13, and S14 and supplementary text, section 8, Supplementary Material online). pAts therefore appear as core replicons of a majority of *At* species. In addition, while many megaplasmids of the same *repABC* family are known to recombine intensely within species (Kumar et al. 2015; Epstein et al. 2012), the occurrence of clade-specific genes on pAts and never on the other plasmids (pTis and smaller ones) suggests the existence of barriers to its transfer. Within Cohan’s ecotype framework, we interpret this pattern as the presence of determinants of the species’ ecological niche on these particular extrachromosomal elements, which selectively prevented their spread among closely related species (Cohan and Koeppel 2008). This suggests that the pAt is probably an essential replicon for most species of *At* in their natural environments and qualifies it as a bona fide chromid (Harrison et al. 2010). Deletion-mutant competition experiments on the distantly related chromid pSymB (diCenzo et al. 2016) demonstrated that the chromid had a significant regulatory impact on the bacterial host and contribution to its fitnesst in the plant rhizopshere (i.e., outside of a symbiotic nodule). Consequently, these megaplasmids possibly play an determining role in adaptation to their core ecological niche (Lassalle et al. 2015). Functional investigation of the core functions borne by agrobacterial pAts could thus provide a better understanding of the specific ecophysiology of each *At* species.

### Clade-Specific Gene Functions Provide Insights into the Possible Ecological Speciation of Clade Ancestors

The nature of putative ecological specialization is not obvious for agrobacteria, which are ubiquitous soil-dwellers. Different *Agrobacterium* species frequently cooccur in soils, sometimes in the same micro-metric sample (Vogel et al. 2003); based on the competitive exclusion principle (Gause 1932), they must have distinct ecologies. Certain soils and/or host plants are preferentially colonized by certain species (Costechareyre et al. 2010). In parallel, G2 members appear to have developed a capacity towards opportunistic pathogenicity in humans (Aujoulat et al. 2011). This shows some kind of niche differentiation occurs among *Agrobacterium* species, but the precise nature of the underlying environmental factors still remains to be decyphered. Because clade-specific genes are expected to encode what makes the ecology of a clade to be distinct from that of its relatives (Lassalle et al. 2015), we investigated the specific functional repertoire of At clades. Strikingly, in most clades, including species or higher-level groups, the sets of clade-specific genes recurrently presented the same classes of functions. These include transport and metabolism of phenolic compounds, aminoacids and complex sugars, and production of exopolysaccharides and siderophores, all of which can be related to bacterial life in the plant rhizosphere (Lassalle et al. 2011).

Among these, we can notably report the specific presence of a supernumerary chemotaxis regulation operon *che2* in species G1, which is uniquely linked to an array of genes with predicted functions involved in the catabolism of (possibly aminated) aromatic compounds (supplementary table S6, Supplementary Material online). This suggests that G1 strains are able to specifically degrade certain—yet unknown—aromatic compounds, for which they might display specific tropism and/or induction of biofilm formation.

G8 species and the [G6–G8] clade presented a number of clade-specific gene clusters (supplementary table S6, Supplementary Material online), as previously reported (Lassalle et al. 2011), among which the largest were the ferulic acid degradation and siderophore biosynthesis operons. These operons have been reported to provide a growth advantage and to be expressed in a coordinated manner in a plant rhizosphere environment (Campillo et al. 2014; Baude et al. 2016). Taken together, these results show that G8 lineage-specific genes jointly participate in the adaptation to a plant-related specific ecological niche. Interestingly, the gain of a siderophore biosynthesis locus in the [G6–G8] clade ancestor coincided with the loss of the locus encoding biosynthesis of another siderophore, agrobactin, otherwise ubiquitous in, and unique to, the *At* clade. This conserved switch to a different pathway for iron scavenging—a crucial function in iron-depleted plant rhizospheres—may provide a competitive advantage with respect to cooccurring agrobacteria.

The [G5–G13] species group specifically presented a phenylacetate degradation pathway operon (supplementary table S6, Supplementary Material online), which biochemical function was demonstrated in vitro (supplementary fig. S15, Supplementary Material online). This discovery readily provides us with a specific biochemical identification test for these species, and again hints to the particular affinity of agrobacteria for aromatic compounds likely to be found in plant rhizospheres.

Finally, the large cluster that encodes the nitrate respiration (denitrification) pathway, including the *nir*, *nor*, *nnr*, and *nap* operons was absent from the [G1–G5–G13] clade. More recently, that gene cluster was also lost by strains G9-NCPPB925 and G8-ATCC31749, and its presence in strain G3-CFBP6623 seems to result from later transfer from a mosaic of sources within *At*. Considering the absence of this super-operon in close relatives of *At* such as *A. vitis* and *R. leguminosarum*, it was likely acquired by the ancestor of the [G2–G4–G7–G9–G6–G8] clade (node N21 on fig. 1), one of the two large clades that divide the *At* complex. Strains possessing the denitrification pathway may be selectively advantaged under certain anaerobic or micro-aerophilic conditions, like those met in certain soils and rhizospheres; such an adaptation may have supported an early differentiation of *At* lineages towards the colonization of partitioned niches.

Species G1 and G8 presented a particular case of convergence of their clade-specific functional repertoire. Firstly, they shared 57 synapomorphic genes (supplementary tables S7 and S8, Supplementary Material online), in most cases with phylogenetic support for transfer events among respective ancestors. These traits were previously hypothesized to provide key adaptation to life in the plant rhizosphere of G8 (=*A. fabrum)* (Lassalle et al. 2011). For instance, these species share homologous genes involved in the biosynthesis of curdlan—a cellulose-like polysaccharide—and the biosynthesis of O-antigens of the lipopolysaccharide (LPS) (supplementary table S6 and supplementary text, section 5.1, Supplementary Material online). These two capsular components may define attachment properties of the cell to the external environment, possibly in a similar way than the LPS synthesized by homologous enzymes in *Brucella spp*., which mediates a specific interaction with cells of a eukaryotic host (Vizcaíno et al. 2001). In addition, nonhomologous G1 and G8 clade-specific genes encoded similar functional pathways, that is, phenolic compound metabolism and exopolysaccharide production (supplementary table S6, Supplementary Material online).

This convergence of the niche-specifying gene repertoires of species G1 and G8 may have caused a stronger overlap of their ecological niches, which in turn might have led to interspecies competition for resources. However, shared niche-specifying genes occur in combination to different sets of species-specific genes in the core-genome of each species, and different epistatic interactions could induce strong divergence in their phenotype. Typically, even though the loci for LPS O-antigen biosynthesis in G1 and G8 are highly similar (>93% amino acid identity in average for proteins of the homologous AtSp14 loci, supplementary fig. S16, Supplementary Material online) and most likely produce a structurally equivalent compound, regulation of biofilm production by these species is probably different. Indeed, several regulatory genes specific to the G1 genomes are involved in the regulation of chemotaxis/biofilm production, such as the *che2* operon (cluster AtSp2) and hub signal-transducing protein HHSS (“hybrid–hybrid” signal-sensing, see supplementary text, section 5.1, Supplementary Material online) found in cluster AtSp14 (supplementary figs. S11 and S16, Supplementary Material online), and a sensor protein (cluster AtSp3) modulating c-di-GMP—a secondary messenger involved in the switch from motile to sessile behaviors. Those specific regulators were all in close linkage to G1-specific genes involved in phenolics catabolism or biofilm production. These latter genes may be the downstream regulatory targets of what seems to be a coherent regulation network controlling motility, biofilm production and phenolics degradation; this locus is potentially coding for a whole pathway for responses to specific environmental conditions of the niche of G1, such as the availability of phenolics to use as nutrients. Similarly, G8-specific genes of the AtSp26 cluster (supplementary fig. S12, Supplementary Material online) formed a regulatory island involved in the perception and transduction of environmental signals, including mechanosensitive channels and a receptor for phenolic compound related to toluene (Lassalle et al. 2011).

Both the G1 and G8 species are thus likely to orchestrate the production of similar polysaccharides under different regulation schemes, involving the coordination of their expression with other specific traits—in both cases the catabolism of (likely different) phenolics. Similarly, coordinated expression of several clade-specific genes resulting in conditional phenotypes has recently been observed in G8-C58 (Baude et al. 2016), strengthening the idea of the existence of an ecological niche to which species G8 is specifically adapted through the expression of a particular *combination* of clade-specific genes. The partial hybridization of the G1- and G8-specific genomes probably led each species to tap the same resources in different ways, avoiding any significant competition between them. These species may thus form guilds of relatives that exploit partitions of a largely common ecological niche (Lassalle et al. 2015), enabling them to cooccur in soils (Vogel et al. 2003; Portier et al. 2006).

Although such evolutionary mechanisms of late hybridation and reassortment of niche-specifying genes have previously been observed (Sheppard et al. 2013), it is unclear whether they are common among other soil/rhizosphere-dwelling bacteria. A recent investigation of the pangenome diversity of *R. leguminosarum* genomic species revealed similar patterns of occurrence of species-specific genes, but none could be related to a species-specific metabolic or symbiotic property, challenging the notion that species could have specific ecological adaptations (Kumar et al. 2015). However, this study only relied on the analysis of the pattern of homologous gene presence/absence, not their gain history, and could have overlooked parallel synapomorphic gene gains. Using our estimation of scenarios of gene evolution, we see that convergent evolution was important in shaping *At* genomes (supplementary tables S7 and S8, Supplementary Material online) and that ecological niche differentiation may occur through finer processes, including specific regulation of complex sets of functions.

## Conclusion

We developed an original method to estimate the history of all genes in a bacterial pangenome and applied it to the *Agrobacterium* biovar 1 species complex (*At*) to unveil the gain and loss dynamics of the gene repertoire in this taxon. Genes specifically gained by major *At* clades were mostly organized in large blocks of co-evolving genes that encode coherent pathways. This pattern constitutes a departure from a neutral model of gene transfer in bacterial genomes and indicates purifying selection has enforced their conservation. We therefore considered these blocks of clade-specific genes as likely determinants of clade core ecologies. Genes specific to each species and to the *At* species complex as a whole recurrently encoded functions linked to production of secreted secondary metabolites or extracellular matrix, and to the metabolism of plant-derived compounds such as phenolics, sugars, and amino acids. These clade-specific genes probably represent parallel adaptations to life in interaction with host plant roots. This suggests that ecological differentiation of *Agrobacterium* clades occurred through the partitioning of ecological resources available in plant rhizospheres. In the future, sampling of within-species diversity, coupled with population genomics approaches, could further reveal ecological properties of agrobacteria, including those that may be nonubiquitous but dynamically maintained by recombination within species (Kashtan et al. 2014; Rosen et al. 2015). Gene coevolution models, such as the one developed here, could be extended to the investigation of interlocus linkage in genome populations (Cui et al. 2015). Such analyses could reveal complex interactions between molecular pathways under ecological selection, opening onto new steps towards the understanding of bacterial adaptation to the infinite diversity of microenvironments.

## Supplementary Material

Supplementary data are available at *Genome Biology and Evolution* online.

## Supplementary Material

Supplementary Materials
