# Supplementary material for: Ancestral Genome Estimation Reveals the History of Ecological Diversification in Agrobacterium
Source: Genome Biol Evol. 2017 Dec 6;9(12):3413–31. doi: 10.1093/gbe/evx255 (PMC5739047; doi:10.1093/gbe/evx255)
Supplement: Supplementary Materials [file evx255_supp.zip › genomes-agro_SupText_rev2_FL7.pdf]

Supplementary Material

Table of contents

I Legends of Supplementary Figures.......... p. 2
II Supplementary Tables.......... p. 4
III Supplementary Files.......... p. 4
IV Supplementary Text.......... p. 5
1 Comparison of several hypotheses for the core-genome reference phylogeny.......... p. 5
2 Construction of Agrogenom database.......... p. 7
3 Reconciliation of gene trees with the species tree.......... p. 8
4 Block event inference: algorithms.......... p. 10
1 Definitions of concepts.......... p. 10
2 Algorithm 1: Construction of leaf blocks.......... p. 11
3 Algorithm 2: Construction of ancestral blocks.......... p. 12
5 Clade-specific genes: insights into the ecological properties of clades.......... p. 13
1 Genomic synapomorphies of G1.......... p. 13
2 Genomic synapomorphies of G8 and [G6-G8] clade.......... p. 16
3 Genomic synapomorphies of [G5-G13] clade.......... p. 17
4 Genomic synapomorphies of [G1-G5-G13].......... p. 17
5 Genomic synapomorphies of the *A. tumefaciens* complex.......... p. 17
6 Selected cases of large transfer events.......... p. 18
7 Secondary replicons of *Agrobacterium* genomes bear clade-specific innovations.......... p. 19
8 References.......... p. 20

I Legends of Supplemental Figures

Fig. S1: Phylogeny of 131 genomes of Alpha-proteobacteria.
This tree was obtained by maximum-likelihood (ML) from the concatenated alignment of 61 universal unicopy gene families using RAxML (version 7.0.4) (Stamatakis, 2006) with PROTMIXWAGF model (with 25 rate categroris) and with a start tree obtained from the consensus (using CONSENSE program from PHYLIP package (Felsenstein, 1993), MRE rule) of the 61 individual ML trees obtained with PhyML (Guindon and Gascuel, 2003) under a LG+4G model. The tree is rooted as proposed in Williams et al. (2007) based on the branching of outgroups; the purple arrow indicates an alternative root proposed in Abby et al. (2012) that minimized the number of horizontal transfer events in a reconciliation approach. The Rhizobiaceae clade is highlighted in green. Species that were integrated in the 47-Rhizobiales dataset are coloured in red. Tip names correspond to those defined in Table 1 for the red labels and to the Uniprot 5-letter code of organisms for the remainder of the tree (http://www.uniprot.org/docs/speclist).

Fig. S2: Rereference phylogeny of Rhizobiales history.
Obtained by consensus of ML trees built from concatenates of 500 jackknife samplings of 25 genes among the 455 unicopy genes from the core of the 47 genomes. Supports of short branches within *S.meliloti* were all ≥ 0.9.

Fig. S3: Bioinformatic pipeline for reconciliation of gene and genome histories.
(1-6) Detection of horizontal transfer events; (7) Inference of gene content of ancestral genomes and integration of reconciliations with evolutionary scenarios; (8,9) Estimation of blocks of co-evolved genes.

Fig. S4: Algorithm for construction of blocks of co-transferred genes.
A. Course of the extension of a block. A gene detected transferred (green ’T’ boxes) is encountered during the course of the replicon (1). The block is initiated (2) and extended to gene having a consistent signal transfer (other ’T’ boxes) or no signal of transfer (boxes ’?’), In which case a gap is opened (3). When the extension leads to create a gap greater than size $g = 2$, the block is stopped (4), then extremal genes without signal for a transfer are removed from the block (5). The search for block events resumes after the last gene block (6).
B. Concordant or discordant scenarios of transfer. A block of events has an overall scenario corresponding to the intersection of those of its constituent genes. The scenarios are described as sets of possible receiver (Rec) and donor (Don) nodes, as exemplified Fig. 2. The dark green block consists of genes whose transfer scenarios are identical (light green boxes, light green scenario) and gap genes without signal: the block has an overall scenario identical to light green genes (1). The block is extended to the brown gene for which the sets of possible receivers and donors are smaller: the global scenario sees its coordinates refined accordingly (2). When continuing the march on the chromosome to extend the dark green block, a pink gene is met; the donor set of the dark green block and that of the pink gene have an empty intersection: the scenarios are considered discordant, and the block is stopped (2); a new block is initiated (3). **C-D. Checking the compatibility of ’gap’ genes with the scenario of the block.** C. Test if the scenario of the transferred genes (green boxes) is rejected by the ’gap’ genes (blue boxes). D. Breaking of an inconsistent block into several blocks and re-computation of their respective scenarios.

Fig. S5: Alternative reference tree topologies obtained with different methods.
(A) Consensus of ML trees obtained from concatenated alignments of jackknife gene samples (500 draws of 25 genes among 455 core unicopy genes), branch supports are based on their frequency in the 500 jackknife trees; (B) ML tree obtained from concatenated alignments of ribosomal protein genes, branch supports are based on 1,000 random bootstrap replicates ; (C) ML tree obtained from concatenated alignments of 455 core unicopy genes, branch supports are based on their frequency in 200 bootstrap replicates.

Fig. S6: Support for monophyply of groups in all gene trees.
Support for grouping of species into different clades, including some that are absent in the reference phylogeny (left), was tested in every gene tree. Tested groups are shown as pairs of clades in the reference tree, indicated by brackets on the right. Fractions indicate the count of gene families in the Agrogenom database that matched a pattern of monophyly over the number of families for which the monophyly could

be tested (i.e. given the set of represented species covers representatives of the clades).

Fig. S7: Hierarchical clustering of *Rhizobiales* genomes according to their gene content.
Hierarchical clustering was performed with the Neighbour Joining algorithm on euclidean distances computed from the occurence profiles of 41,664 gene families (possibly several counts) in extant genomes. Computations and plots were made using R package 'ape' (Popescu et al. 2012).

Fig. S8: Distribution of sizes of block events. (A) Leaf block event sizes (maximum number of genes in a genome amongst genes linked to the same ancestral block event). (B) Ancestral block event sizes (number of unique gene lineages, i.e. clades derived from unique gene tree events).

Fig. S9: Gene gain, loss and conservation within *At* clade ancestors.
Node numbers as in Figure 1 and Table S4.

Fig. S10: Residuals of negative exponential regression of clade age vs. conservation of gained genes. Node numbers as in Figure 1. The regression is define as:

$$cg \sim \exp(-26.038 \cdot age + 5.042)$$

with *cg* the conserved gains. The regression has the following summary statistics:

$$\mathrm{Cov}(\log(cg), age) = -0.0269;\ \mathrm{sd}(age) = 0.0321;\ \mathrm{sd}(\log(cg)) = 1.165$$

Residues in red fall outside of the 95% confidence interval of the regression. Clade names are indicated below corresponding species tree node labels when above the species level, or otherwise indicate in parenthesis the name of the species including the clade.

Fig. S11: Historical stratification of gains in the lineage of the *Agrobacterium sp.* G1 *s*train TT111. The four replicons of the genome are represented circularly or linearly according to their molecular topology; replicons are not drawn to scale. Tracks within outermost ring (lowermost layer for linear chromosome) represent location of CDSs on both DNA strands. Other rings (layers) show genes that were acquired along the history of the TT111 lineage, and are labelled (*a*-*d*) according to the species phylogeny in the right inset.. Colors of genes indicate their specific presence in one of the clade that includes TT111, or their common specific sharing with another clade (see legend box). Outer (lower) vs. inner (upper) tracks in the same rings (layers) distinguish clade-specific genes with strict vs. relaxed specificity criterion. Numbered frames show particular gene clusters within TT111 genome: (1-4) G1-specific clusters: (1) AtSp2: chemotaxis regulation (*che2*) and aromatic compound metabolism locus; (2) AtSp3: phenolics and amino-acid catabolism; (3,4) AtSp7 and AtSp9: phenolic compounds downstream degradation; (5-8), clusters specifically shared by G1 and G8: (5) AtSp14: lipopolysaccharide O-antigen biosynthesis and neoglucogenesis locus with G1-specific chemotaxis-regulating hybrid sensor (red arrow; red gene in Fig. S10); (6) AtSp12: outer-membrane lipoprotein and sensory protein; (7) AtSp17: deoxyribose uptake and assimilation; (8) AtSp15: exopolysaccharide (curdlan) synthesis, peptidoglycan modification and sensory protein; (9-10): clusters gained by TT111: (9) AtSp29: non-ribosomal peptide synthases involved in siderophore biosynthesis, shared by [G8-G6]; (10) prophage, partially shared by G3-CFBP6623 and G7-Zutra 3/1 (see Fig. S14).

Fig. S12: Historical stratification of gains in the lineage of *Agrobacterium sp.* G8 (*A. fabrum*) strain C58. Legend as in Fig. S11. Numbered frames show particular gene clusters within C58 genome: (1-4) G8-specific gene clusters: (1) Atsp21: degradation of hydroxy-cinamic acids (ferulic acid); (2) AtSp23: degradation of complex amino-acids (opine-like compounds); (3) AtSp24 and AtSp25: Drug/toxic resistance (extrusion transporters), sarcosine oxidase; (4) AtSp26: sensing of environmental signals (phenolic compound, mechanical constrains); (5-8) clusters specifically shared by G1 and G8: (5) AtSp15: exopolysaccharide (curdlan) synthesis, peptidoglycan modification and sensory protein; (6) AtSp13: iron-sensing two component system FeuPQ; (7) AtSp14: lipopolysaccharide O-antigen biosynthesis; (8) AtSp12: outer-membrane lipoprotein and sensory protein; (9-12) [G6-G8]-specific gene clusters: (9) AtSp29: sugar (L-sorbose) uptake and catabolism; (10) AtSp30: non-ribosomal peptide synthases involved in siderophore biosynthesis, shared by G1-TT111 (see Fig. S11); (11) AtSp31: sugar metabolism; (12) dipeptide uptake and degradation; cluster specifically shared by G1 and [G6-G8]: (13) AtSp18: D-glucuronate uptake and degradation; (14-15) clusters specifically shared by G2 and G8: (14) AtSp27: Toxic extrusion / secondary metabolite secretion; (15) AtSp28: xanthine/cyclic compound degradation, two-component sensor.

Fig. S13: Historical stratification of gains in the lineage of *A. sp* G4 (*A. radiobacter*) strain B6. Legend

as in Fig. S11. Numbered frames show particular gene clusters within B6 genome: (1-3) G4-specific gene clusters: (1) Atsp37 : aromatic compound (acriflavine) perception, degradation and efflux; (2) Atsp38 : uptake and catabolism of sugars (sorbose, dehydro-fructose) (shared by [G5-G13] clade); (3) Atsp39 : gamma-glutamyl cycle for detoxification of periplasmic compounds; (4-5) [G4-G7-G9]-specific gene clusters: (4) Atsp40 : uptake and degradation of a (sulfated) polygalacturonide/polyglucuronide; (5) Atsp41 : ferrichrome-iron sensing and uptake. Note that the major part of pTiB6 is shared by G1-TT111, as was previously shown (Lassalle et al., 2011), suggesting that pTiB6 and pTiTT111 are related by a recent transfer event.

Fig. S14: Historical stratification of gains in the lineage of *A. sp. G7* strain Zutra 3/1.
Legend as in Fig. S11. Numbered frames show particular gene clusters within Zutra 3/1 genome: (1-4) G7-specific gene clusters: (1) Atsp42: Quorum sensing- regulated periplasmic protein-disulfide isomerase DsbABG (ensures proper conformation of disulfide-bonded proteins like c-type cytochromes for covalent haem attachment); (2) AtSp46: prohibitin (DNA synthesis represession), phage shock protein (represses the expression of sigma54-dependent operons); (3) AtSp47: system for sensing (FecIR), TonB-dependent import (FhuACD) and utilization (ViuB) of iron(3+)-hydroxamate siderophore; (4) AtSp48: enzymes and regulators of unknown function.

Fig. S15: Growth curves of representative of *At* genomic species on phenylacetate
Agrobacterium strains grown overnight in AT medium supplemented with succinate and ammonium sulfate were inoculated at an optical density at 600 nm (OD_{600}) of 0.05 in 200 µl AT medium supplemented with appropriate carbon and nitrogen sources.

Fig. S16: Syntenic conservation of the AtSp14 cluster in G1, G8 and Brucelaceae.
Microscope MAGE's view of the locus in G1-H13-3 linear chromosome (top), with a projection of syntenic genes found in compared genomes (bottom), including (from top to bottom) genomes of G1 (rows 1-4), G8 (rows 5-7), G13 (row 8) and G5 (row 9), as well as genomes of selected Brucellaceae (rows 10-16). These genes code the biosynthesis of an O-antigen decoration of the lipopolysaccharide (LPS) ; HHSS gene is coloured in red. View accessible at https://www.genoscope.cns.fr/agc/microscope/mage/viewer.php? when selecting *Agrobacterium sp.* H13-3 chromosome CP002248 as a reference with window ranging from genomic positions 400,000 to 480,000.

II Supplementary Tables

Table S1: Statistics of the 16 new genome sequences.

Table S2: List of the 455 universal unicopy gene families. The list of sequences belonging to these gene families and corresponding gene trees can be obtained from searching the family identifier in the 'Family Accession' field at http://phylariane.univ-lyon1.fr/db/agrogenom/3/search/

Table S3: Matrix of presence/absence of the 49 ribosomal gene families in the 47 Rhizobiaceae genomes. The list of sequences belonging to these gene families and corresponding gene trees can be obtained from searching the family identifier in the 'Family Accession' field at http://phylariane.univ-lyon1.fr/db/agrogenom/3/search/

Table S4. Bioinformatic pipeline for homologous database construction and Gene tree/Species tree reconciliation.

Table S5: Statistics of gains and losses per contemporary and ancestral genome and replicon

Table S6: Location and functional description of clade-specific gene clusters in *A. tumefaciens* genomes

Table S7: Summary of clade-specific genes in TT111 genome.

Table S8: Summary of clade-specific genes in C58 genome.

III Supplementary Files

S1 Datatset: Lists of clade-specific genes per clade. The same colour code for clade-specific and specifically shared genes is used than in Figures S11, S12, S13 and S14 for the respective clades: G1 and [G1-G5-G13] as in Figure S11; G8 and [G6-G8] as in Figure S12; G4 and [G4-G7-G9] as in Figure S13; G7 as in Figure S14.

IV Supplementary Text

1 Comparison of several hypotheses for the core-genome reference phylogeny

Studies using data from the whole genome of bacteria showed in many case that almost each locus of the genome has a distinct history, i.e. supports a unique tree topology (Abby et al. 2012; Shapiro et al. 2012). Several methods exist to summarize the main phylogenetic signal of a genome-wide dataset, among which the most commonly used is the concatenation of alignments of conserved genes. Such "super-matrix" approaches can recover a majority signal from a large dataset, even if it is hidden by a large amount of noise due to phylogenetic irresolution and HGT. This global approach is powerful, but has a notable drawback: it does not document what fraction of genome supports the tree inferred from the main signal. In fact, it has been shown that the main tree can be representative of only a handful of gene trees among the hundreds that are inferred for individual loci, even though the main tree is strongly supported by high bootstrap or likelihood supports (Abby et al. 2012).

The simple supermatrix tree approach is thus too extremely opposed to the observation of all possibly different gene tree topologies. To satisfy this trade-off, we used a a jackknife resampling methodology that allowed us to recover the main signal from genome-wide data, putting it in perspective with the amount of genes that support it. We thus proceeded in sampling 500 times (drawing without replacement) sets of 25 gene families among the 455 unicopy core gene families (i.e. families with exactly one copy in our 47 Rhizobiales genomes), for which the alignments were concatenated and trees computed with PhyML (version 3.0, GTR+ 8+I model of evolution, best of SPR and NNI moves, SH-like supports) (Guindon & Gascuel 2003). The extended majority-rule consensus of these 500 jackknife trees was then computed to reflect the main phylogenetic signal of the dataset, using the frequency of its bipartitions in the jackknife sample to reflect the fraction of the core genome in support of this main signal; we refer to the output of this method as Unicopy Core Jackknife Consensus (UCJC). We tried alternative methodologies and/or datasets to explore the robustness of our refenrece phylogeny: using the concatenation of the whole set of 455 universal unicopy gene families (Unicopy Core Concatenante, UCC) or a concatenation of 49 ribosomal protein gene families (Ribosomal Concatenate, RC) (Table S3), in both case to compute trees with RAxML (version 7.2.8, GTRCAT model, 50 discrete site-heterogeneity categories) (Stamatakis 2006).

The consensus tree from UCJC approach is presented Figure S2. It groups together all named genera and species, exception made of strain *Rhizobium sp*. PD01-76 which groups with *A. vitis* S4, suggesting strain PD01-76 must belong to a clade related to *Agrobacterium* biovar 3. Focusing on *Agrobacterium* biovar 1, i.e.

A. tumefaciens species complex (*At*), all genomic species were found monophyletic with high support, and grouping of some genomic species based on *recA* or *telA* marker gene phylogenies (Costechareyre et al. 2010; Shams et al. 2013; Ramírez-Bahena et al. 2014) were recovered with good support: G8 and G6 (hereafter named [G6-G8] clade), G1 with G5 and G13 ([G1-G5-G13] clade), G7 with G9 ([G7-G9] clade) and G4 with the latter ([G4-G7-G9] clade). Also, the single representative of G2 groups with [G4-G7-G9] clade, in accordance to some studies but not all. Altogether, comparisons of the UCJC tree to the RC and UCC trees (Figure S5) and to phylogenies previously built with marker genes yield one robust feature: the separation of [G4-G7-G9] and [G1-G5-G13] clades marking a bifurcation at the root of *At*. The remaining groups, G2 and G3 species and [G6-G8] clade, have a labile positioning within *A. tumefaciens*. Notably, *At* clade has the the same topology in the tree based on UCC and UCJC methodologies, but is rooted differently within Rhizobiaceae, making G2 strain and [G6-G8] clade to flip root sides (Figure S5 A,C).

The basal position of G2 and G3 in these core phylogenies is linked to long branches leading to those species that have only one representative strain (Figure 1), suggesting a possible artefact of long branch attraction. The basal position of these two species and their long branches are observed neither in classic conserved marker genes like *recA* and *gyrB* phylogenies when two strains per species are present in the dataset (Ramírez-Bahena et al. 2014; Costechareyre 2007), nor in the RC tree based on slowly evolving ribosomal protein genes (Figure S5 B), comforting the hypothesis of an estimation artefact due to insufficient sampling and/or saturation of phylogenetic signal. Alternative topologies found in *recA*, *gyrB* (Ramírez-Bahena et al. 2014; Costechareyre 2007) and RC phylogenies (Figure S5 B) share common features: the grouping of G2 strains directly with G9, and the grouping or at least proximity of G3 strains with [G6-G8] clade. These hypotheses of grouping based on slowly evolving markers could therefore be good proposals for an optimal reference tree.

As the species tree is intended as a reference for reconciliation of gene and genome histories, the optimal topology is the one the most representative of the history of the pangenome, i.e. resulting in the most parsimonious pangenome-wide count of evolutionary events when reconciling incongruent gene trees. We thus tested which hypothesis of grouping had the strongest support by assessing the prevalence of relevant branching patterns within a larger set of gene phylogenies – as opposed to relying on the sole unicopy core genes, which represent only ~10% of each genome. We wished to make no assumption on the preferred qualities (e.g. evolutionary rates) of the genes to be considered and thus chose to exploit the information from all the gene families in the pangenome dataset. For this, we used the program TPMS (Bigot et al. 2013) to search the collection of gene trees for the occurrence of particular phylogenetic patterns signing the monophyly of different groups of strains (Figure S6). Phylogenetic patterns are defined as topological constraints on the local branching pattern of (groups of) species – here specifically the monophylly of pairs of clades. These patterns are matched against subtrees where at least one representative of each target groups was present, with a minimum branch support of 0.9 at the root of the monophyletic pattern. Searches were limited to subtrees containing only *At* members and rooted by an outgroup ensuring the appropriate rooting of the subtree. Gene trees can contain multiple copies of gene per species and thus several matches can be found for the pattern; in the presence of in-paralogs, different matches can be made on overlapping leaf sets. To account for this possible bias, we used as an output score the number of unique genes found at the leaves of any matching subtrees. For each pattern search, another control pattern was searched for the occurrence of the same set of species but without constrains on their relative branching, to obtain the number of unique genes for which the branching hypothesis could be tested. We searched for the monophyletic grouping of different pairs of clades of *At*, as summarized in Figure S6. The branching of G2 with [G4-G7-G9] clade, as

in UCJC tree, is found for only 27% of unique genes, but this hypothesis achieves a better score than the alternative hypothesis of G2 branching directly with G9 (12%). Similarly, the grouping of G3 with [G1-G5-G13] clade is also rather infrequent (29%), but still slightly more frequent than the monophyly of G3 and [G6-G8] clades (26%). These low fractions of gene trees supporting the queried patterns could be due to the reduced probability of observing the pattern when only a single representative of the clade was sampled. To test for such an effect, we searched patterns matching well-supported monophylies involving species with a single representative strain. We were able to find that 45% of single G9 strain genes were found supporting its monophyly with G7, 53% of G13 genes supporting grouping with G5, and 71% of G6 genes supporting grouping with G8, which confirms that the TPMS search for monophyly patterns involving single-strain species has the relevant power, and that rather it is the position of G2 and G3 in the species tree which is subject to strong variation depending on the considered gene. Additionally, we found that [G6-G8] clade grouped with [G4-G7-G9] in 71% of cases – though this rely on a small number of testable genes (824), probably due to the constrain of finding representative of each species in these clades monophyletic in gene trees – further supporting the topology that was proposed by the UCJC tree (Figure S5 A) but not in the RC and UCC trees (Figure S5 B,C). In summary, even though some nodes of the UCJC tree are supported by only in a minority of core genes, its topology notheless appears the best compromise in representing the main signal for the whole pangenome history of *At* and thus provides the most parsimonious reference phylogeny for reconciliation of genome and gene histories in this group.

2 Construction of Agrogenom database

Our pipeline for database construction is mostly that of Hogenom databases (Penel et al. 2009) up to the gene tree building and is summarized here.

Genomes were retrieved from ENA (http://www.ebi.ac.uk/ena/) and Microscope (https://www.genoscope.cns.fr/agc/microscope/) databases (Vallenet et al. 2013). The genome sequences of the dataset and details about new sequences from this study are listed Table S1. EMBL flat files were used to build an ACNUC database (Gouy et al. 1985) subsequently used for sequence retrieval from keyword-based queries. This ACNUC database is freely accessible through the client tool Query (http://pbil.univ-lyon1.fr/databases/acnuc/acnuc.html); the name of the database ‘agrogenom’ must be typed in the top box, and the relevant password ‘LCSA’ must then be typed in the prompted box.

CDS sub-sequences were extracted and translated to protein sequences that were compared through an all-against-all BLASTP run (Altschul et al. 1990). The BLAST hits matrix was used to build a similarity network with SiLiX program (version 1.2.5-p1, default parameters: proteins are linked if they present a hit of ≥ 35% similarity over ≥ 80% length) (Miele et al. 2011) which was in turn used to infer families of homologous proteins with HiFiX program (version 1.0.3, default parameters) (Miele et al. 2012). Gene family information was stored in the ACNUC database and protein sequences were retrieved by families in Fasta format.

Protein families were aligned using MUSCLE (version 3.8.31, default parameters) (Edgar 2004) and then retro-translated with pal2nal program (version 14) (Suyama et al. 2006). Nucleic alignments were restricted to conserved blocks with Gblocks (version 0.91b, minimum 50% of sequences in conserved and flank positions and all gaps allowed, DNA mode) (Castresana 2000) and gene trees were computed from these alignment with PhyML (version 3.0, GTR+ 8+I model of evolution, best of SPR and NNI moves, SH-like supports) (Guindon & Gascuel 2003).

A PostgreSQL relational database, Agrogenomdb, was built (implemented in PostgreSQL) by an extension of the Phylarianne schema (http://phylariane.univ-lyon1.fr/) to house data relative to genomes, gene and species trees, reconciliations, block events and functional annotations. A UML diagram of the database schema is accessible at https://github.com/flass/agrogenom/blob/master/pipeline/database/scheme_agrogenom_db.pdf. This database is accessible through an interactive graphical web interface at http://phylariane.univ-lyon1.fr/db/agrogenom/3/

3 Reconciliation of gene trees with the species tree

Inferrence of pangenome evolutionary history was done using a specially developed bioinformatic pipeline. It is mostly constituted of shell and Python scripts (relying notably on Python module tree2, https://github.com/flass/tree2) that allow manipulation of phylogenetic trees and provide an API to interact with diverse third-party phylogenetic programs and the relational database Agrogenomdb, where all reconciliation data were stored. This pipeline implements a stepwise procedure for genetree/species tree reconciliation, as described below and on the dedicated Github repository (where all cited scripts and related documentation can be found):

https://github.com/flass/agrogenom/tree/master/pipeline

Following steps of the procedure are illustrated in Figure S3 (see corresponding numberred boxes), the corresponding computer commands are described online (Github repository), and their standard input/output is described in Table S4:

1. Rooting of gene trees with TPMS program (Bigot et al. 2013) using the combo criterion (*tpms_computations* program, 'RC' command) minimizing both unicity of subtrees and their taxonomic depth (knowing the species phylogeny). Root minimizing these criteria favours reconciliation scenarios more parsimonious in ancient events (implying many losses) of duplication and transfer, respectively. Unicopy gene trees were however rooted using a transfer event parsimony approach, using Prunier to test all roots of the gene tree and to select the one which induces the lower number of transfers (using root_as_prunier.py script).

2. Using the *find_ancestral_duplications.py* script, subtree pruning and regrafting (SPR) moves were attempted on weakly supported branches of gene trees (SH-like support < 0.9) in order to minimize the number of duplication events (merging events) or the number of tree leaves involved (bring events closer to the tips). Branch lengths and supports were re-estimated with PhyML (Guindon & Gascuel 2003) using the same model as previously. Subtrees of (co-)orthologous (unicopy) genes to be tested for transfers with Prunier (Abby et al. 2010) were then identified as follows: all possible combination of unique representants of the species in a lineage defined by duplication node – supposed to be (co-)orthologs (Kristensen et al. 2011) – were extracted, with each gene being potentially represented in several gene combination sets; the majority of nodes in the full gene tree are thus covered by several unicopy subtrees that will are used as independent input for Prunier for HGT inferrence.

3. Run of Prunier (version 2.0, fast algorithm, forward search depth = 2, branch support threshold = 0.9) on each unicopy subtree to detect transfer events based on statistically supported phylogenetic conflict

between species tree and unicopy subtree of genes.

4. Mapping of each of these independent transfer inferences to nodes of the full gene tree using *rec_to_db.py* script ; nodes covered by several independent Prunier tests may have been or not detected as a transfer depending on the test, and several inferences of transfer may yield different scenarios (transfer direction and/or precision of the mapping to the reference species tree, see detailed procedure in Annex 1 at https://github.com/flass/agrogenom/tree/master/doc/).

5. Block event reconstution using *get_block_events.py* script (see detailed algorithm description in Section 4 and Annex 2 at https://github.com/flass/agrogenom/tree/master/doc/). At gene tree nodes for which several possibilities of event were retained, the choice of the event was made based on the following criteria: chosing the event that was found in the largest block event, and in case of ties, choosing the event that was inferred in the majority of cases among Prunier replicates on different unicopy subtrees.

6. Completion of the gene tree/species tree reconciliation either by inference of transfers in presence of taxonomic incongruence (detected using the taxonomic oncongruence search algorithm introduced in TPMS software (Bigot et al. 2013)) or by inference of duplications.

7. Ancestral gene contents inference through estimation of the history of gene gain and loss independently for each sub-family of orthologs (obtained by pruning subtrees from the full gene tree at every node annotated as a duplication or transfer). Inference of gain/loss scenarios was done using the Wagner parsimony algorithm implemented in Count software (Csűrös 2008), which returns the most parsimonious combination of gene gains and losses given a gain/loss penalty ratio. The latter parameter was set to 1 for a best compromise between keeping coherent ancestral genome sizes (penalizing losses enough to have the genome size of an ancestor smaller or equal to that of its children) and coherent gene histories (penalizing gains enough to avoid multiple gains happening among groups of closely related strains). This method infers several gains that are not mapped to the LCA of the species represented in the subtree, and these additional gains were interpreted as transfers, which were subsequently mapped to nodes in the full gene trees. The transfer events inferred here are less reliable since they are not supported by topological incongruences between the gene and species tree. Nevertheless, they greatly improves the reconciliation in case of clade-specific groups of genes by avoiding to map their acquisition too high in the species phylogeny and inferring excessive counts of losses.

The reconciliation of gene trees can be translated into an evolutionary scenario in the species tree, where presence/absence states and the duplication, transfer and speciation events are mapped on the reference tree (Figure S3, step 7).

The complete reconciliation scenario were then integrated into the Agrogenom database, that notably stored the gradual process of inferences, with the intermediary data such as the possible coordinates in the species tree of the various transfer, duplication and speciation events inferred at steps 2-6.

4 Block event inference: algorithms

The leaf blocks are built following Algorithm 1, a greedy algorithm of systematic exploration of gene histories along replicons to find compatible events across gene families. The ancestral blocks are built accretion of leaf blocks following Algorithm 2.

The algorithms description rely on the definition of a set of variables and helper function, which description relies on a certain number of concepts:

Definitions of concepts:

A *gene tree event* is annotated at the node of a reconciled gene tree to explain the topology of the gene tree given the reference species tree. It can be an origination (O), a speciation (S), a duplication (D) or a horizontal transfer (T).

The *location* of an evolutionary event is the mapping of the event in the species tree, i.e. the identification of the ancestor in which the event occurred. It consists of the label of the node representing the ancestral lineage. Horizontal transfers are characterized by a dual location: that of the donor and that of the recipient lineage. In a complete reconciliation, the location of an event in the species tree should correspond to only one node, e.g. N_1 (or a pair for transfers, e.g. $N_1 \rightarrow N_5$). To achieve better sensitivity in further search for event location similarity, the reconciliation is not completed by the determination of the count and location of loss events, and thus the inferred locations of events remain uncertain; uncertain locations of events are represented by a set of alternatively possible ancestral node labels (Figure 1), e.g. $N_1|N_2|N_3 \rightarrow N_4|N_5$.

Events are *compatible* if they have a non-null intersection of their sets of possible locations in the species tree.

A *leaf block event* is a block of gene tree events involving genes occurring consecutively in an extant genome, where these gene tree events are compatible; the refined location of the leaf block event is the intersection of locations of member gene tree events.

An *ancestral block event* is agglomeration of homologous leaf-block events, i.e. that involve the same gene tree events. Ancestral block events represent the actual events that occurred in the past and involved the respective common ancestors of extant genes grouped in leaf block events. The refined location of the ancestral block event is the intersection of locations of member leaf block events. The leaf block events gathered in an ancestral block should be compatible because by definition they are built from homologous events; however, heterogeneous presence of gene families in extant genomes may lead to incompatibilities between putative homologous leaf blocks, revealing their spurious assembly and preventing consistent accretion of ancestral leaf blocks; a heuristic is used to split conflicting leaf blocks until all parts are compatible with putative homologous leaf blocks, after what ancestral block events are built

The algorithm description below are of rather high level and ommit the description of the heuristics used in the split, merge and resolve operations. These are described in detail in Annex 2 at https://github.com/flass/agrogenom/tree/master/doc/.

Algorithm 1: Construction of leaf block events.

The following pseudo-code describes the procedure operated on each replicon (autonomous DNA molecule in a genome) in the database, in the particular case where no gap in the leaf block events are allowed, and when considering a transfer (T) event; for origination (O) and duplication (D), the event locations are defined by a single set of possible species tree node, and the compatibility of two such events is simply the non-null intersection of their locations.

INPUT:

- a replicon R, which is a vector of r genes $(G_1, \dots, G_r)$
- a collection $\{T\}$ of all reconciled gene trees, of which every internal node n is annotated with a gene tree event E, and which every leaf is associated to a gene G,
- a map $\mathcal{Location}$ associating any event object X (a gene tree event, a leaf block event, or ancestral block event) to its location in the species tree, which is a set of species tree nodes, such as: $\mathcal{Location}(X) \rightarrow (d_X, r_X)$ with d_X the set of possible donor species $\{s_a, s_b, \dots\}$ and r_X the set of possible recipient species $\{s_u, s_v, \dots\}$. It initialy contains values associated to all gene tree events.

OUTPUT:

- a list $\mathcal{B}$ of leaf block events on replicon R

Notation:

- we define as a lineage L in a gene tree T as the vector of nodes $(n_1, \dots, n_l)$ located on the path from the tip representing gene G to the root of T.

```
function LocationOverlap(X, Y):
    Let d_X, r_X = Location(X) and d_Y, r_Y = Location(Y)
    r_XY = r_X ∩ r_Y
    d_XY = d_X ∩ d_Y
    return (d_XY, r_XY)

function CompatLocation(X, Y):
    d_XY, r_XY ← LocationOverlap(X, Y)
    if (r_XY ≠ ∅ and d_XY ≠ ∅) then return True
    else return False

procedure BuildLeafBlocks( R, {T} )
    Let 𝓑 ← ∅
    Let 𝓖𝓔 ← ∅                    // initiate an empty list of (gene, gene tree event) 2-tuples
    for i = 1 to r do
        Let G_i be the i-th gene on replicon R
        Let L_i be the lineage of gene G_i on its gene family tree T_i
        for each node n_i,j in L_i do          // explore the gene tree events above gene G_i
          Let E_i,j be the event associated to n_i,j
          if (G_i, E_i,j) not in 𝓖𝓔
            Let B ← { (G_i, E_i,j) }
            Let Location(B) ← Location(E_i,j)
            𝓖𝓔 ← 𝓖𝓔 + (G_i, E_i,j)
            Let c ← True, k ← 1
            while (c is True) and ( i+k <= r ) do
              Let G_k be the k-th right-hand side neighbour gene of G_i
              Let L_k be the lineage of gene G_k on its gene family tree T_k
              for each node n_k,l in L_k do       // explore the gene tree events above gene G_i
                Let E_k,l be the event associated to n_k,l
                if CompatLocation(B, E_k,l) then
                  B ←B + (G_k, E_k,l)
                  Location(B) ← LocationOverlap(B, E_i,j)
                  𝓖𝓔 ← 𝓖𝓔 + (G_k, E_k,l)
                  break for                  // when found matching event, stop the search iteration at node n_k
                else
                  if n_k,l is root(T_k) then
                       c = False              // root of G_k lineage has been reached without finding a matching event
                  end if
                end if
              end for
              k = k+1
            end while
            𝓑 ← 𝓑 + B                        // store the leaf block in the global list to be returned
          end if
        end for
    end for
    return 𝓑
    end procedure
```

Algorithm 2: Construction of ancestral blocks.

The following pseudo-code describes the procedure operated on a set of leaf block events as generated with **Algorithm 1** from every replicon in the database (i.e. covering all extant genomes). It associates leaf block events with unique ancestral block events. Simply, this procedure searches connections between leaf block events via any gene tree event they include; a same ancestral block event is referenced for every leaf block events that share such connection. Compatibility between leaf block event coordinates is then tested and when positive, the ancestral block event coordinates are derived from their intersection. In case of incompatibility, a heuristic is used to resolve it by splitting spurious leaf blocks into smaller ones until they can associate with compatible counterparts without conflict.

INPUT:

- the list of all leaf block events $\mathscr{B}$

OUTPUT:

- the list of all ancestral block events $\mathscr{A}$
- a map *Ances* recording the association of each leaf block B to its respective ancestral block $Ances[B] \to A_B$
- a map *Location* recording the location of any event object X (see **Algorithm 1**). It initialy contains values associated to all gene tree events and leaf block events.

Notation:

- for definition of helper functions RESOLVE and MERGE, please refer to Annex 2 of the extended documentation online (https://github.com/flass/agrogenom/blob/master/doc)

```
procedure BUILDANCESTRALBLOCKS( ℬ )
𝒜 ← ∅
for each leaf block B in ℬ do
   // search for already existing ancestral blocks linked to gene tree events from B
   𝒜_B ← ∅                          // initiate an empty list of related ancestral block events
   for each gene tree event E in B do
        Let 𝒜_E be the subset of 𝒜 including all ancestral blocks connected to event E
        𝒜_B ← 𝒜_B + 𝒜_E
   end for
   if ( 𝒜_B is ∅ ) then
      A = { B }                      // instantiate a new ancestral block
      Location(A) ← Location(B)
      Ances[B] ← A                   // store the ancestral block in leaf block-to-ancestral block map
      𝒜 ← 𝒜 + A                      // store the ancestral block in the global list to be returned
   else
      for each ancestral block A_p in 𝒜_B do
         if not COMPATLOCATION(B, A_p) then
            Let (B', B", … ) be the result of RESOLVE(A_p, B)  // split leaf block B into parts compatible with A_p (B') or not ( B", … )
            ℬ = ℬ + (B', B", … )                              // append new sub-blocks to the global leaf block list
            break                                             // the case of B is dropped, its sub-parts will be evaluated later
         else
            if Ances[B] is ∅ then
               Ances[B] ← A_p
            else
               Ances[B] ← MERGE(A_p, Ances[B])
            end if
         end if
      end for
   end if
end for
return Ances, (A)
end procedure
```

5 Clade-specific genes: insights into the ecological properties of clades

In the following text, we will describe what gene sets are specific to clades of *A. tumefaciens* and present the main functions they encode. Those clade-specific genes are mostly located in relatively large clusters which often encode coherent metabolic or physiological pathways. These gene clusters will be systematically labelled with the prefix AtSp followed by a numeric suffix. This nomenclature aims to name homologous clusters uniquely across the study and transversally among different genomic context (Table S6).

5.1 Genomic synapomorphies of G1

There are 78 genes present in all G1 members and in no other strains of At and 87 genes present in all G1 and sparsely distributed in other clades, totalizing 165 G1-specific genes (S1 Dataset). In addition, 108 genes present in all G1 members are also found specifically shared with other clades, including 43 genes shared with G8 and 24 with [G6-G8] (Table S7). The large majority of those two kinds of specific genes are grouped in clusters (84/165 G1-specific genes, and 99/108 of genes specifically shared with other species). These clusters are in general annotated with coherent functions (Table S6, Figure S11). Briefly, G1-specific gene clusters can be linked to a restricted set of cellular pathways, which are detailed below.

Chemotaxis and phenolic/aromatic compound degradation pathways

One main gene cluster occupies a 24-kb locus on the circular chromosome next to a rRNA operon (AtSp2, frame 1 in Figure S11). It contains several oxydases, mono-oxygenases and amino-transferases which predicted functions together seem to participate in the degradation of one – or several diverse - aromatic compounds that may be aminated. This last feature is further supported by the cognate presence of a predicted amino-acid ABC transporter. Lastly, this cluster encodes and a complete chemotaxis operon including several methyl-accepting chemotaxis proteins (MCPs) and histidine kinases (HK) (Wibberg et al. 2011) that must mediate the transduction of signal for the presence of chemoattractant(s) potentially related to the aromatic compounds degraded by linked enzyme genes. All Rhizobiaceae have one primary chemotaxis regulation operon *che1* and this secondary one, *che2*, is found only in G1 among *A. tumefaciens*. This nine-gene chemotaxis cluster *che2* is shared by *Rhizobium etli, R. leguminosarum* and *A. vitis*/*R. sp.* PDO1-076 clade, but not the rest of the locus which is rather distantly related to *Mesorhizobium ciceri*. This assemblage of chemotaxis and catabolic genes is thus unique to G1.

A second large locus of the linear chromosome (AtSp4, frame 2 in Figure S11) is found specific to G1 when relaxing the stringency of specificity definition. While several other *A. tumefaciens* strains bear the genes of this locus, the pattern of occurrence outside G1 is heterogeneous across the 50-kb locus and the relations of non-G1 and G1 genes in phylogenetic trees are rarely direct, or in a way indicating a transfer from G1 to the other agrobacteria. Among the functions encoded in this locus, there are notably several mentions of isochorismatase hydrolase, and multiple occurrences of monooxygenase, dioxygenase and peroxygenase enzymes that are often involved in ring-cleavage reactions. In particular, the locus encodes a perhydrolase (non-heme chloroperoxidase) and a multimeric aldehyde oxidase whose homologs are documented as non selective on their substrate (Song et al. 2006; Yasuhara et al. 2005). These genes likely code generalist degrading enzymes that may be involved in detoxication of complex aromatic compounds and/or their catabolism for growth. Interestingly, the locus also harbours a sensory diguanylate cyclase/phosphodiesterase which can regulate the motile/sessile behaviour of the cell through control of cytoplasmic c-di-GMP concentration.

Two other loci on the pAt (AtSp7 and AtSp9) also contain several genes that code enzymes involved in downstream degradation of possibly alkylated phenolic compounds.

It happens that some gene among species-specific gene clusters are found present in other isolated strains of *A. tumefaciens*, showing that gene transfer is occurring frequently, but most of times they result in a very partial sharing of biochemical pathways encoded by clade-specific gene clusters, and thus the potential niche-specifying functions were likely not transmitted. However, some species-specific gene clusters are consistently shared by unrelated strains and sometimes species, showing that gene transfer among species may result in propagation of potentially niche-specifying functions. Here are some cases of gene clusters specifically shared by G1 and other distant species, classified by the general function they confer.

Amino-acid catabolism

There are two loci that are specifically shared by all G1 strains and the only sampled strain of G2 (AtSp3 and AtSp5). They encode two amino-acid ABC transporters and each one is associated to an enzyme gene: an agmatinase, involved in degradation of polyamines, and a monooxygenase, involved in degradation of aromatic compounds. In addition, there are G1-specific genes located further on the linear chromosome that code a tetrameric enzyme resembling a sarcosine oxidase. Together, these features indicate an ability of G1 to import and degrade specific amino-acids and polyamines.

G1+G8: Exopolysaccharide biosynthesis and cell motility regulation

There are five jointly specific gene clusters shared by G1 and G8 species (Table S6), including one coding for lipo-polysaccharide (LPS) O-antigen biosynthesis (AtSp14), another for curdlan polysaccharide biosynthesis (AtSp15), one for deoxyribose uptake and assimilation (AtSp17), and two two-componet regulation systems (AtSp13, AtSp16); all these functions can be related to capsule/biofilm production and regulation of cell motility. Clusters AtSp15 and AtSp17 are found on the At plasmids (pAt) in G1 strains but on the linear chromosomes in G8 strains.

AtSp14 is a 45-kb locus of the linear chromosome of G1 strains that is highly similar to its homolog in G8 (>93% amino-acid identity averaging over 29 proteins shared by H13-3 and C58). The parsimony approach for ancestral gene content estimation (see Sup. Text section 3) inferred AtSp14 locus was gained twice in *A. tumefaciens* history, potentially by transfer from one species to the other. They are both distantly related to one locus found in Brucellaceae (>51% amino-acid identity averaging over 25 proteins shared by H13-3 and *B. melitensis* 16M). The homologous locus in Brucella was identified as encoding a pathway for biosynthesis of a LPS O-antigen (Vizcaíno et al. 2001). Brucellar LPS are extensively described in the literature (reviewed in Cardoso et al. (Cardoso et al. 2006)) because they can confer a smooth phenotype which helps the pathogen to escape recognition by the immune system of the host. Though, the role of these particular genes in the biosynthetic process of LPS is not documented. Since this locus is absent from *B. abortus* genomes compared to those of *B. melitensis* and *B. suis*, one could relate the occurrence of these genes to the differences in O-substitutions of the core LPS between *B. abortus* and *B. melitensis*, the so-called the A and M antigens (Meikle et al. 1989; Kubler-Kielb & Vinogradov 2013). Conserved parts of this [G1-G8]-specific locus are not found in Brucellaceae, including genes involved in neo-glucogenesis from storage polysacchraride (like glycogen), that potentially participate in mobilizing the cell’s resources to support exoploysaccharide production. Similar functional association can be hypothesized AtSp17 cluster coding deoxyribose uptake and assimilation.

One particular gene of the AtSp14 locus is specific to [G1-G8] compared to Bucellaceae: AGROH133_10197 (in H13-3 genome) codes a sensory protein-coding gene that has a different structure in G1 and G8. In G8 genomes, this gene encodes a hybrid sensory histidine kinase-response regulator, whose homolog in G1 appears to be fused to another signal transduction protein: a hybrid CheR-CheB methyl-esterase/methyl-transferease. The G1-encoded ’hybrid-hybrid’ signal-sensing protein (HHSS) should be able to sense an environmental (cytoplasmic) signal, to be auto-regulated and to transduce the signal to other

regulatory proteins by phosphorylation and by methylation – notably the chemotaxis regulation proteins – and also to bind DNA to regulate transcription of genes. The surrounding LPS locus is likely part of the target regulated genes, either by direct DNA binding or by signal transduction mediated by the two LuxR-like response regulators encoded in the locus, one being located right next to the HHSS gene.

The genomic region AtSp15 encoding the biosynthesis of curdlan is found in G8, G1 and G2 strains, but their sequences are highly divergent compared to the rest of the genome (86% identity for the complete genomes of strains G8-C58 and G1-TT111 vs. 71% between AtSp15 loci, ranked in the 2% most divergent fragments between genomes). This distant homology probably confers different functions to the diverged proteins (76% average amino-acid identity over the locus) that may synthesise different forms of the curdlan polymer. This divergence also explains why this cluster in G1 genomes was not recognised as homologous to G8-C58 genome using comparative genome hybridization, as the DNA hybridization threshold lays around 80-85% nucleotide identity (Lassalle et al. 2011).

The common regulation of exo-polysaccharide biosynthesis and motility has already been observed in *A. tumefaciens* G8 strain C58 (Xu et al. 2013), in the related organism *Rhizobium* sp. NT-26 (Andres et al. 2013) and in other bacteria (Barraud et al., 2009; Marchal et al., 2010). This has to do with the switch between motile ans sessile lifestyles, polar flagellum-mediated swimming being opposed to adhesion and formation of biofilm. This transition is in general commanded by the intra-cellular amount of cyclic di-guanylate (c-di-GMP) which can bind the PilZ domain (Schirmer & Jenal 2009; Hickman & Harwood 2008; Krasteva et al. 2010) notably found in cellulose synthase subunit A common to all *A. tumefaciens*. This second messenger is regulated by the relative activity of diguanylate cyclases and phosphodieasterases present in the cell, with high c-di-GMP levels leading to a sedentary lifestyle (Kolter & Greenberg 2006; Xu et al. 2013). In fact, this secondary messenger is much likely involved in regulating local c-di-GMP concentration in compartments of the cell to promote anisomorphous phenotypes like unipolar polysaccharide production (Xu et al. 2013).

In G1, there seems to exist a system of regulation of chemotaxis/EPS production that integrates sensing of more environmental stimuli. Indeed, among the two enzymes active on c-di-GMP coded by G1-specific genes, one (AGROH133_13181) has a sensory PAS domain and is located in the locus AtSp3 that encodes a phenolic compound degradation pathway, suggesting a regulation of c-di-GMP relative to the perception of a substrate for the neighbour gene-coded enzymes.

Altogether, there seem to be in G1 a specific regulon integrating regulation of motility, biofilm synthesis and phenolic compound trophism. These cellular processes would be regulated, on one hand through modulation of c-di-GMP concentration and on the other hand, via the activity of a multi-functional protein, HHSS. The role of this hub protein in G1 cell physiology is certainly central and investigating it is certainly of great interest. For instance, targeted deletion mutagenesis of this gene and of regions coding its sensory, DNA-binding or several signal transduction domains would likely have great influence the cell transcritome and interactome and probably yield strong macroscopic phenotypes such as impaired swimming or modified biofilm production. In fact, phenotypic differences have been observed between G1 and G8 strains that were not predicted given the gene occurrence profiles: curdlan production genes and deoxyribose assimilation genes are specifically shared by G1 and G8, but production of a strongly Congo Red dye-binding curdlan is a specific phenotype of G8 in rich-medium culture conditions (Lassalle et al. 2011) and degradation of 2-deoxyribose was observed to be specific to G1 strains (Vial L., Bourri M., personal communication). This suggests that from a common set of genes and encoded functions, G1 and G8 could have different response to their environment through differential regulation of gene expression.

G1+G9: Extra-cellular secretion

One feature specifically found in G1 At plasmids (AtSp11) and shared by strain G9-Hayward0363, the unique representative of G9, is the presence of a complete type 1 secretion system (T1SS). The canonical T1SS components are accompanied by a putative periplasmic hydrolase and a hybrid enzyme whose first moiety is related to MurF and catalyses the last step of murein biosynthesis and the second moiety is related to glycosylases, suggesting that enzymes modifies the peptidoglycan to help the proper inclusion of T1SS complex in it. There is also one uncharacterized protein with repetitive structure suggesting possible adhesion properties, which might constitute the secreted product. This locus may have an important role in secreting biofilm constituents as it has been shown in *Acinetobacter* (Loehfelm et al. 2008) or to establish interaction with a host extra-cellular matrix.

5.2 **Genomic synapomorphies of G8 and [G6-G8] clade**

The species G8 is characterized by 58 genes present in all G8 strains and in no other At strains, and 42 other genes present in all G8 and shared with a heterogeneous set of strains from other clades, totalizing 100 G8-specific genes (S1 Dataset). In addition, 71 other genes are found specifically shared with other clades, notably with G1 (Table S8). A large fraction of the two gene sets (62/100 G8-specific genes, 69/71 genes specifically shared with other species) are located in clusters of two to more than thirty contiguous genes (Table S6, Figure 14). This specific gene repertoire and its organization in clusters generally match previous findings based on micro-array hybridization experiments (Lassalle et al. 2011), with correspondences with former cluster denomination indicated Table S6.

Considering genes specific to G8 and those specific to clade [G6-G8], we mostly found the same genes than in our previous comparative genomics analysis focused on G8 (Lassalle et al. 2011) and their arrangement in clusters is very similar. For the sake of coherence of denomination throughout this manuscript, these clusters will be renamed with the AtSp nomenclature used above; correspondence with former cluster names from Lassalle et al. (Lassalle et al. 2011) is indicated Table S6.

AtSp21, is the largest G8-specific gene cluster located on the circular chromosome. It contains the operon *braCDEFG*, that encodes an ABC transporter of amino-acids with broad-range specificity, and genes coding enzymes of the ferulic acid degradation pathway, that were recently characterized for their expression and molecular functions (Campillo et al. 2014). AtSp29 cluster, which is found immediately upstream, is specific to [G6-G8] clade and is dedicated to transport and catabolism of sugars and amino-acids. It includes an enzyme (encoded by Atu1408 in C58 genome) which predicted activity of transformation of L-sorbose into L-iditol echoes the specific ability of G8 strains to degrade L-sorbose (Vial L., Bourri M., personal communications).

On the linear chromid, a cluster encoding curdlan exopolysaccharide biosynthesis, AtSp15, was formerly thought to be specific to G8 (Lassalle et al. 2011) but appears specifically shared by the only representative strain of G2 and all strains of G1. Several other G8-specific or [G6-G8]-specific gene clusters are found on the linear chromosome (Table S6), consistently with previous results (Lassalle et al. 2011). In addition, we found clade-specific gene clusters on the At plasmid of G8 strains: G8-specific gene cluster AtSp28 codes the degradation of xanthine or another related cyclic compound and [G6-G8]-specific gene cluster AtSp32 putatively codes the uptake and degradation of dipeptides that include an aromatic amino-acid. The gain of a siderophore biosynthesis locus (AtSp30) in the ancestor of [G6-G8] coincided with the loss of the locus coding biosynthesis of another siderophore, agrobactin, which is otherwise ubiquitous in – and unique to – the *At* clade.

5.3 Genomic synapomorphies of [G5-G13] clade

Thirty-five genes are specific to [G5-G13] (63 when including genes occurring heterogeneously in other *At* strains) (S1 Dataset). Among those, three gene clusters (AtSp34-36) are encoding potential ecologically relevant functions, namely a transporter of oligopeptides, a peptide methionine sulfoxide reductase (oxidative stress response) and the complete pathway for degradation of phenylacetate. The latter metabolic ability was shown to provide a growth advantage to G5 and G13 strains in presence of phenylacetate (Table S6; Figure S15).

5.4 Genomic synapomorphies of [G1-G5-G13]

The large cluster conserved in agrobacteria (AtSp33, Atu4381-Atu4410 in G8-C58 genome) which encodes nitrate respiration (denitrification) pathway, including *nir*, *nor*, *nnr* and *nap* operons was lost in this clade. This gene cluster was parallely lost by strains G9-NCPPB925 and G8-ATCC31749 (for this one this could be an artefact of draft assembly having missed this genomic region, though this is not probable given its size). These strains devoid of the denitrification pathway may be selectively disadvantaged under certain anaerobic conditions. This is not certain however, because other anaerobic respiration pathways are predicted to be conserved in all *A. tumefaciens*, notably the fumarate respiration pathway.

5.5 Genomic synapomorphies of the *A. tumefaciens* complex

Among the 2,919 core genes of the *At* clade, 171 represent genomic synapomorphies; 127 are exclusive to *At*, and 44 are also present in at most two other distant Rhizobiales strains, i.e. excluding the *At*'s sister taxon *A. vitis* (S1 Dataset). *At*-specific genes generally form smaller clusters than species-specific genes (at most 6-gene long vs. up to 30 genes in G1 and G8), fewer gather genes with apparently concerted functions, with the exception of transport of metabolites. Broad categories of At-specific genes can be defined as follows:

Central metabolism

Gain of a methylenetetrahydrofolate reductase MetF, needed to regenerate tetrahydrofolate, related to that specifically present in G8 (AtSp21) (G8 has 2 copies).

There are notable events of non-homologous gene displacements enriching the functional repertoire of *At*: the loss of a NADPH-dependent glutamate synthase (49RHIZOB_2054 gene family) is concomitant with the gain of an isoenzyme of another type (gltB1, 49RHIZOB_3984), together with a sulphite reductase (cysJ) involved in cysteine biosynthesis, both putatively associated to Calvin cycle anabolism (AtSp49).

Phopho-gycerate mutase is an essential enzyme of the glycolysis coded by two genes in Rhizobiaceae, *gpmA* and *gpmB*. In *A. tumefaciens*, *gpmA* is lost but functionally replaced by non-homologous gene of rhodobacterial origin, *gpmI* which codes an iso-enzyme. GmpA and GmpB belong to the family of phopho-gycerate mutase needing 2,3-bis-phospho-glycerate as a cofactor and show high specificity to their substrate mono-phopho-glycerate. GmpI family enzymes do not need a cofactor and catalyse less efficiently the interconversion of phopho-glycerate. They are however more promiscuous in the substrate they can phosphorylate (Rigden et al. 2002), and could therefore provide new kind of phosphorylated compounds, and notably sugars that are integrated to complex polysaccharides, or membrane lipids. This can be linked to the presence of an extra diverged copy of glpD, coding for glycerol-3-phosphate dehydrogenase which is involed in biosynthesis of phospholipids.

Cell wall and outer membrane

Gain of cell wall teichoic acid biosynthesis enzyme (*tagA*, Atu0587) and two succinoglycan biosynthesis transporters (Atu0588 and *mdoC*, Atu3522). Gain of putative scaffold protein for murein sacculus septation (MipA). Presence of specific succinoglyclan EPS biosynthesis genes: *exoU* and a specific extra copy of

exoQ.

Informational processes
Gain of two rRNA modification enzymes (RsmJ and RluA). Loss of a second copy of rpoH. Gain of protelomerase TelA, enabling the linearization of the linear chromid, the major synapomorphy of *At* (Ramírez-Bahena et al. 2014). The history of DNA polymerase III subunit alpha gene family is complex: the copy of *dnaE* shared by all *A. tumefaciens* and mostly borne on the linear chromosome is of distant origin compared to other Rhizobiales, and many strains bear one or several extra copies of more distant type which is more prone to transfer and most often plasmid-borne.

Sensing
Multiple gains of two-component systems, dyguanylate cyclases/esterases and methyl-accepting proteins coupled to sensor domain (AtSp58, AtSp60, AtSp64).

Carbohydrate metabolism
Several gains of transporter and catabolic enzymes for amino-acids or poly-amines (AtSp57, AtSp60, AtSp65).

Iron metabolism
Gain of one free iron transporter (FbpAB), 2 iron-siderophore ABC transporter, including iron-hydroxamate transporter FhuABCD used for uptake of iron-complexed agrobactin, an iron storage protein (Dps), an heme oxygenase and precorin hydrolase (ChiG) involed in heme degradation/transformation.

Gain of genes for biosynthesis of secondary metabolites, including phosphopantetheinyl transferase (*agbD*) catalyzing the fixation of phosphopantethein prosthetic group to catalytic modules of NRPS/PKS mega-enzymes, among which the ones responsible for biosynthesis of agrobactin and G6-G8-specific siderophore.

Detoxification
Two P-type ATPases for extrusion of copper and heavy metals, respectively, putative nickel ABC transporters and a MFS permease of unknown specificity.

6 Selected cases of large transfer events

An integrative and conjugative element (ICE) was transferred between strains G4-Kerr14 and G7-Zutra 3/1. The circular chromosome of Kerr14 and the linear chromosome of Zutra 3/1 are found almost 100% identical over 125 kb (genomic coordinates on CcKerr14: from 2,490kb to 2,615kb). This shared locus is unique to these two strains among *A. tumefaciens*. It contains genes encoding a complete type IV secretion system, partition enzymes and a resolvase-type recombinase at an extremity sign the nature of this locus as an ICE. Right next to the recombinase gene is located a tRNA gene and a rRNA operon, which must have been the substrate of the recombinase for the integration of the ICE.

It bears cargo genes, among which several catabolic genes involved in the uptake and degradation of several compounds, among others: phenolics and degradation products (benzaldehyde, benzoylformate, toluate, catechol, muconate, mandelate), nitrilated compounds (acrylonitrile), sugars and derivated polyols (xylulose, tagatose, sorbitol, xylitol, ribitol). There are also genes for a lipoprotein and an outer membrane

protein of RopB family. The many genes involved in the uptake and degradation of phenolic and sugar derivatives, suggesting that this mobile element could confer selective advantages to these two strains in the adequate environment.

A large block event highlighted the relatedness of 70-kb plasmid pIV from strain G6-NCPPB925 and p79 from *A. vitis* strain S4, which appears to be circularized ICEs, as indicated by the presence of resolvase genes. The common ancestor of these elements must be quite ancient, since their average nucleotide similarity is of 75%, and because most genes they share are associated to their mobility while the metabolic genes they harbor are different.

A 12-kb mobile element bearing mercuric resistance genes was transferred between G3-CFBP6623 and G5-6626 very recently (genes almost 100% identical).

A Mu-like prophage was transferred several times between strains of *A. tumefaciens* and related strains S4 and PDO1-076, with variable sizes of conserved parts.

The only large ancient transfer event we could identify was the transfer between the ancestors of G1 and G8 of a 45-kb DNA segment encoding the lipopolysaccharide (LPS) O-antigen biosynthesis pathway, which constitutes a specific character of both clades (Table S6, AtSp12; Figure S11, S12 and S16).

7 Secondary replicons of Agrobacterium genomes bear clade-specific innovations.

Rhizobiaceae present complex genomic architectures composed of a primary chromosome and a secondary chromosome or megaplasmid bearing essential genes called chromid (Harrison et al. 2010), and a variable complement of plasmids of various sizes (Young et al. 2006). More specifically, the chromid of the *Agrobacterium* genus (Mousavi et al. 2015; Ormeño-Orrillo et al. 2015), which includes the *At* clade, is linear (Slater et al. 2009, 2013) as the result of an unique ancestral event of linearization and thus the linear chromid (Lc) constitutes a synapomorphy of this clade (Ramírez-Bahena et al. 2014). Another general feature of *At* genomes is the frequent presence of a pAt, a megaplasmid which was for long referred to as the cryptic plasmid, for its role in the cell biology remains largely unknown. These pAts belong to the larger family of *repABC* (mega-)plasmids, which can conjugate between a broad range of hosts among Rhizobiaceae, as described for symbiotic (Sym) or tumor-inducing (Ti) plasmids in *Rhizobium* and *Agrobacterium*, respectively (González et al. 2003; Gonzalez et al. 2010; Lassalle et al. 2011). In addition, it is well known that pTis can be transferred between strains of different species of *At*, as confirmed by the large similarity observed here between the pTiB6 and pTiTT111 (Figure S13). This suggests that pAts could similarly be transferred amongst species of *At*.
However, we found that pAt types are restricted to certain genomic backgrounds, with the phylogeny of their replication genes *repA* and *repC* consistent with *At* species delineation; all species representatives group togheter, apart in the case of G4 pAts, from which G8, G6 and G2 pAts seem to have emerged via HGT (see interactive phylogenies links below*). In addition, pAts host gene clusters specific to the host genome's species (namely G1, G8, G4 and G7 species) or higher groups ([G6-G8] clade) (Figures S11, S12, S13, S14), interspersed with genes that are mostly strain-specific. This species restriction pattern and the occurrence of clade-specific genes on the pAt and never on the other plasmids (pTi and smaller ones), in face of the putative ability of the plasmid to transfer widely, suggests a existence of barriers to the transfer of pAts. Within Cohan’s ecotype framework, we interpret this pattern as the presence of determinants of the species' ecological niche on this particular extra-chromosomal element, which selectively prevents its spread among closely related species (Cohan & Koeppel 2008). This suggests that – for most species of *At* – this third

replicon is probably essential in natural environments, which would qualify it as a *bona fide* chromid (Harrison et al. 2010).
Interestingly, the only apparent inter-species transfers of pAts (from the *repAC* perspective) involve G4 species, in which the species-specific gene cluster AtSp39 (Table S6; Fig. S13) is not fixed on the pAt but can instead be located on the Lc (strains G4-Kerr-14 and G4-CFBP5621), whereas in other densely-sampled species (G1, G7 and G8) the pAt-borne species-specific genes are stably located. This suggests that in the G4 species, the pAt may not have yet become fixed as a chromid. In the particular case of G8 species, 25 G8-specific genes and 11 [G6-G8] clade-specific genes were retrieved on the pAts of the corresponding strains. Our previous study using micro-arrays with G8-C58 genome as a reference did not detect G8-specific genes on plasmids, notably because the G8 strain LMG-46 lacked a pAt (Lassalle et al. 2011). This feature is however unique to the strain within G8 species, which could result from a recent plasmid loss with rare prevalence in the species. Similarly, the only available isolate of G9 species, strain Hayward 0363, has no detectable plasmid. In all thoe cases, further sampling of wild population is required to test whether the presence/absence of a pAt and its suggested species-specific status are the rule, or if intermediate prevalences and unstable carriage of core can occur. With respect to the data presented here, the rule seems to be that pAts are core replicons for most *At* species.

* repA and repC interactive phylogenies are accessible at:
http://phylariane.univ-lyon1.fr/db/agrogenom/3/tree/49RHIZOB_2821_0/none
http://phylariane.univ-lyon1.fr/db/agrogenom/3/tree/49RHIZOB_2823_0/none

References

Abby SS, Tannier E, Gouy M, Daubin V. 2010. Detecting lateral gene transfers by statistical reconciliation of phylogenetic forests. BMC Bioinformatics. 11:324–324. doi: 10.1186/1471-2105-11-324.

Abby SS, Tannier E, Gouy M, Daubin V. 2012. Lateral gene transfer as a support for the tree of life. Proc. Natl. Acad. Sci. 109:4962–4967. doi: 10.1073/pnas.1116871109.

Altschul SF, Gish W, Miller W, Myers EW, Lipman DJ. 1990. Basic local alignment search tool. J. Mol. Biol. 215:403–410. doi: 10.1016/S0022-2836(05)80360-2.

Andres J et al. 2013. Life in an arsenic-containing gold mine: genome and physiology of the autotrophic arsenite-oxidizing bacterium Rhizobium sp. NT-26. Genome Biol. Evol. 5:934–953. doi: 10.1093/gbe/evt061.

Ané C, Larget B, Baum DA, Smith SD, Rokas A. 2007. Bayesian Estimation of Concordance among Gene Trees. Mol. Biol. Evol. 24:412–426. doi: 10.1093/molbev/msl170.

Bigot T, Daubin V, Lassalle F, Perrière G. 2013. TPMS: a set of utilities for querying collections of gene trees. BMC Bioinformatics. 14:109. doi: 10.1186/1471-2105-14-109.

Campillo T et al. 2014. Analysis of Hydroxycinnamic Acid Degradation in Agrobacterium fabrum Reveals a Coenzyme A-Dependent, Beta-Oxidative Deacetylation Pathway. Appl. Environ. Microbiol. 80:3341–3349. doi: 10.1128/AEM.00475-14.

Cardoso PG, Macedo GC, Azevedo V, Oliveira SC. 2006. Brucella spp noncanonical LPS: structure, biosynthesis, and interaction with host immune system. Microb. Cell Factories. 5:13. doi:

10.1186/1475-2859-5-13.

Castresana J. 2000. Selection of conserved blocks from multiple alignments for their use in phylogenetic analysis. Mol. Biol. Evol. 17:540–552.

Costechareyre D. 2007. Contribution à la définition de l'espèce génomique chez les bactéries par MLSA et détermination du rôle de l'isolement sexuel dans la cohérence des espèces: exploration aux 'bornes' de l'espèce dans le complexe d'espèces génomiques 'Agrobacterium tumefaciens'. Thèse de doctorat, Université Claude Bernard: Lyon, France.

Costechareyre D et al. 2010. Rapid and Efficient Identification of Agrobacterium Species by recA Allele Analysis : Agrobacterium recA Diversity. Microb. Ecol. 60:862–72. doi: 10.1007/s00248-010-9685-7.

Csűrös M. 2008. Ancestral Reconstruction by Asymmetric Wagner Parsimony over Continuous Characters and Squared Parsimony over Distributions. In: Comparative Genomics. Nelson, CE & Vialette, S, editors. Lecture Notes in Computer Science Springer Berlin Heidelberg pp. 72–86. http://link.springer.com/chapter/10.1007/978-3-540-87989-3_6 (Accessed March 7, 2013).

Didelot X, Lawson D, Darling A, Falush D. 2010. Inference of Homologous Recombination in Bacteria Using Whole Genome Sequences. Genetics. 186:1435–1449. doi: 10.1534/genetics.110.120121.

Doyon J-P, Ranwez V, Daubin V, Berry V. 2011. Models, Algorithms and Programs for Phylogeny Reconciliation. Brief. Bioinform. 12:392–400. doi: 10.1093/bib/bbr045.

Edgar RC. 2004. MUSCLE: a multiple sequence alignment method with reduced time and space complexity. BMC Bioinformatics. 5:113. doi: 10.1186/1471-2105-5-113.

Gouy M, Gautier C, Attimonelli M, Lanave C, di Paola G. 1985. ACNUC--a portable retrieval system for nucleic acid sequence databases: logical and physical designs and usage. Comput. Appl. Biosci. CABIOS. 1:167–172.

Guindon S, Gascuel O. 2003. A Simple, Fast, and Accurate Algorithm to Estimate Large Phylogenies by Maximum Likelihood. Syst. Biol. 52:696–704. doi: 10.1080/10635150390235520.

Hickman JW, Harwood CS. 2008. Identification of FleQ from Pseudomonas aeruginosa as a c-di-GMP-responsive transcription factor. Mol. Microbiol. 69:376–389. doi: 10.1111/j.1365-2958.2008.06281.x.

Kolter R, Greenberg EP. 2006. Microbial sciences: The superficial life of microbes. Nature. 441:300–302. doi: 10.1038/441300a.

Krasteva PV et al. 2010. Vibrio cholerae VpsT Regulates Matrix Production and Motility by Directly Sensing Cyclic di-GMP. Science. 327:866–868. doi: 10.1126/science.1181185.

Kristensen DM, Wolf YI, Mushegian AR, Koonin EV. 2011. Computational Methods for Gene Orthology Inference. Brief. Bioinform. 12:379–391. doi: 10.1093/bib/bbr030.

Kubler-Kielb J, Vinogradov E. 2013. The study of the core part and non-repeating elements of the O-antigen of Brucella lipopolysaccharide. Carbohydr. Res. 366:33–37. doi: 10.1016/j.carres.2012.11.004.

Lassalle F et al. 2011. Genomic Species Are Ecological Species as Revealed by Comparative Genomics in Agrobacterium tumefaciens. Genome Biol. Evol. 3:762–781. doi: 10.1093/gbe/evr070.

Loehfelm TW, Luke NR, Campagnari AA. 2008. Identification and characterization of an Acinetobacter baumannii biofilm-associated protein. J. Bacteriol. 190:1036–1044. doi: 10.1128/JB.01416-07.

Meikle PJ, Perry MB, Cherwonogrodzky JW, Bundle DR. 1989. Fine structure of A and M antigens from Brucella biovars. Infect. Immun. 57:2820–2828.

Miele V et al. 2012. High-quality sequence clustering guided by network topology and multiple alignment likelihood. Bioinforma. Oxf. Engl. doi: 10.1093/bioinformatics/bts098.

Miele V, Penel S, Duret L. 2011. Ultra-fast sequence clustering from similarity networks with SiLiX. BMC Bioinformatics. 12:116. doi: 10.1186/1471-2105-12-116.

Penel S et al. 2009. Databases of homologous gene families for comparative genomics. BMC Bioinformatics. 10:S3. doi: 10.1186/1471-2105-10-S6-S3.

Ramírez-Bahena MH et al. 2014. Single acquisition of protelomerase gave rise to speciation of a large and diverse clade within the Agrobacterium/Rhizobium supercluster characterized by the presence of a linear chromid. Mol. Phylogenet. Evol. 73:202–207. doi: 10.1016/j.ympev.2014.01.005.

Rigden DJ, Mello LV, Setlow P, Jedrzejas MJ. 2002. Structure and mechanism of action of a cofactor-dependent phosphoglycerate mutase homolog from Bacillus stearothermophilus with broad specificity phosphatase activity. J. Mol. Biol. 315:1129–1143. doi: 10.1006/jmbi.2001.5290.

Schirmer T, Jenal U. 2009. Structural and mechanistic determinants of c-di-GMP signalling. Nat. Rev. Microbiol. 7:724–735. doi: 10.1038/nrmicro2203.

Shams M, Vial L, Chapulliot D, Nesme X, Lavire C. 2013. Rapid and accurate species and genomic species identification and exhaustive population diversity assessment of Agrobacterium spp. using recA-based PCR. Syst. Appl. Microbiol. 36:351–358. doi: 10.1016/j.syapm.2013.03.002.

Shapiro BJ et al. 2012. Population Genomics of Early Events in the Ecological Differentiation of Bacteria. Science. 336:48–51. doi: 10.1126/science.1218198.

Song JK, Ahn HJ, Kim HS, Song BK. 2006. Molecular cloning and expression of perhydrolase genes from Pseudomonas aeruginosa and Burkholderia cepacia in Escherichia coli. Biotechnol. Lett. 28:849–856. doi: 10.1007/s10529-006-9016-8.

Suyama M, Torrents D, Bork P. 2006. PAL2NAL: robust conversion of protein sequence alignments into the corresponding codon alignments. Nucleic Acids Res. 34:W609–W612. doi: 10.1093/nar/gkl315.

Vallenet D et al. 2013. MicroScope--an integrated microbial resource for the curation and comparative analysis of genomic and metabolic data. Nucleic Acids Res. 41:D636-647. doi: 10.1093/nar/gks1194.

Vilella AJ et al. 2009. EnsemblCompara GeneTrees: Complete, Duplication-Aware Phylogenetic Trees in Vertebrates. Genome Res. 19:327–335. doi: 10.1101/gr.073585.107.

Vizcaíno N, Cloeckaert A, Zygmunt MS, Fernández-Lago L. 2001. Characterization of a Brucella species 25-kilobase DNA fragment deleted from Brucella abortus reveals a large gene cluster related to the synthesis of a polysaccharide. Infect. Immun. 69:6738–6748. doi: 10.1128/IAI.69.11.6738-6748.2001.

Wibberg D et al. 2011. Complete genome sequencing of Agrobacterium sp. H13-3, the former Rhizobium lupini H13-3, reveals a tripartite genome consisting of a circular and a linear chromosome and an accessory plasmid but lacking a tumor-inducing Ti-plasmid. J. Biotechnol. In Press, Uncorrected Proof. doi: 10.1016/j.jbiotec.2011.01.010.

Xu J et al. 2013. Genetic analysis of Agrobacterium tumefaciens unipolar polysaccharide production reveals complex integrated control of the motile-to-sessile switch. Mol. Microbiol. n/a–n/a. doi: 10.1111/mmi.12321.

Yasuhara A, Akiba-Goto M, Aisaka K. 2005. Cloning and sequencing of the aldehyde oxidase gene from Methylobacillus sp. KY4400. Biosci. Biotechnol. Biochem. 69:2435–2438.

Young JPW et al. 2006. The genome of Rhizobium leguminosarum has recognizable core and accessory components. Genome Biol. 7:R34. doi: 10.1186/gb-2006-7-4-r34.
