# Supplementary material for: Ancestral Genome Estimation Reveals the History of Ecological Diversification in Agrobacterium
Source: Genome Biol Evol. 2017 Dec 6;9(12):3413–31. doi: 10.1093/gbe/evx255 (PMC5739047; doi:10.1093/gbe/evx255)
Supplement: Supplementary Materials [file evx255_supp.zip › SupFigs_S1-S16.pdf]

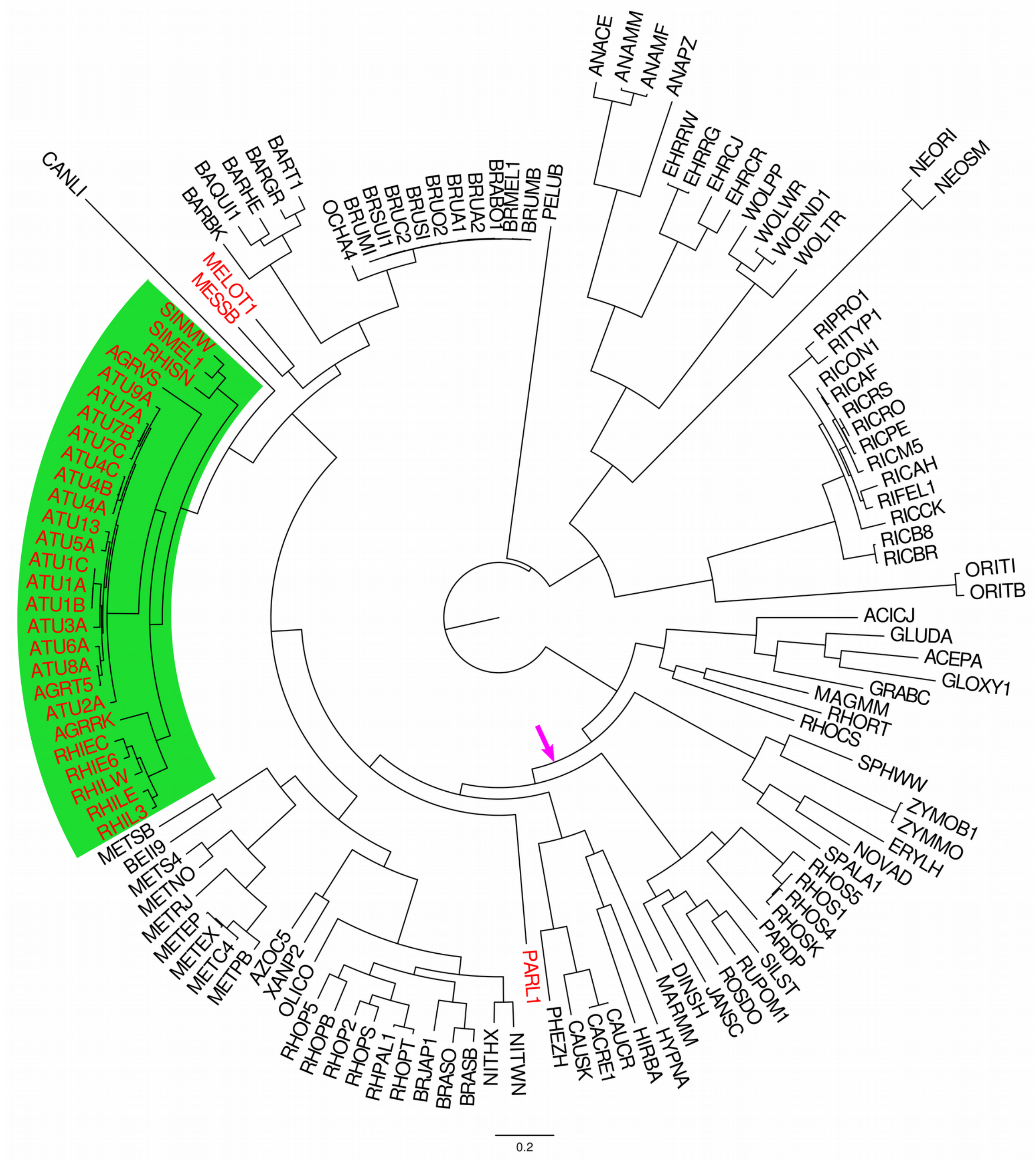

Supplementary Figures

Fig. S1: Phylogeny of 131 genomes of Alpha-proteobacteria.

This tree was obtained by maximum-likelihood (ML) from the concatenated alignment of 61 universal unicopy gene families using RAxML (version 7.0.4) (Stamatakis, 2006) with PROTMIXWAGF model (with 25 rate categroris) and with a start tree obtained from the consensus (using CONSENSE program from PHYLIP package (Felsenstein, 1993), MRE rule) of the 61 individual ML trees obtained with PhyML (Guindon and Gascuel, 2003) under a LG+4G model. The tree is rooted as proposed in Williams et al. (2007) based on the branching of outgroups; the purple arrow indicates an alternative root proposed in Abby et al. (2012) that minimized the number of horizontal transfer events in a reconciliation approach. The Rhizobiaceae clade is highlighted in green. Species that were integrated in the 47-Rhizobiales dataset are coloured in red. Tip names correspond to those defined in Table 2 for the red labels and to the Uniprot 5-letter code of organisms for the remainder of the tree (http://www.uniprot.org/docs/speclist).

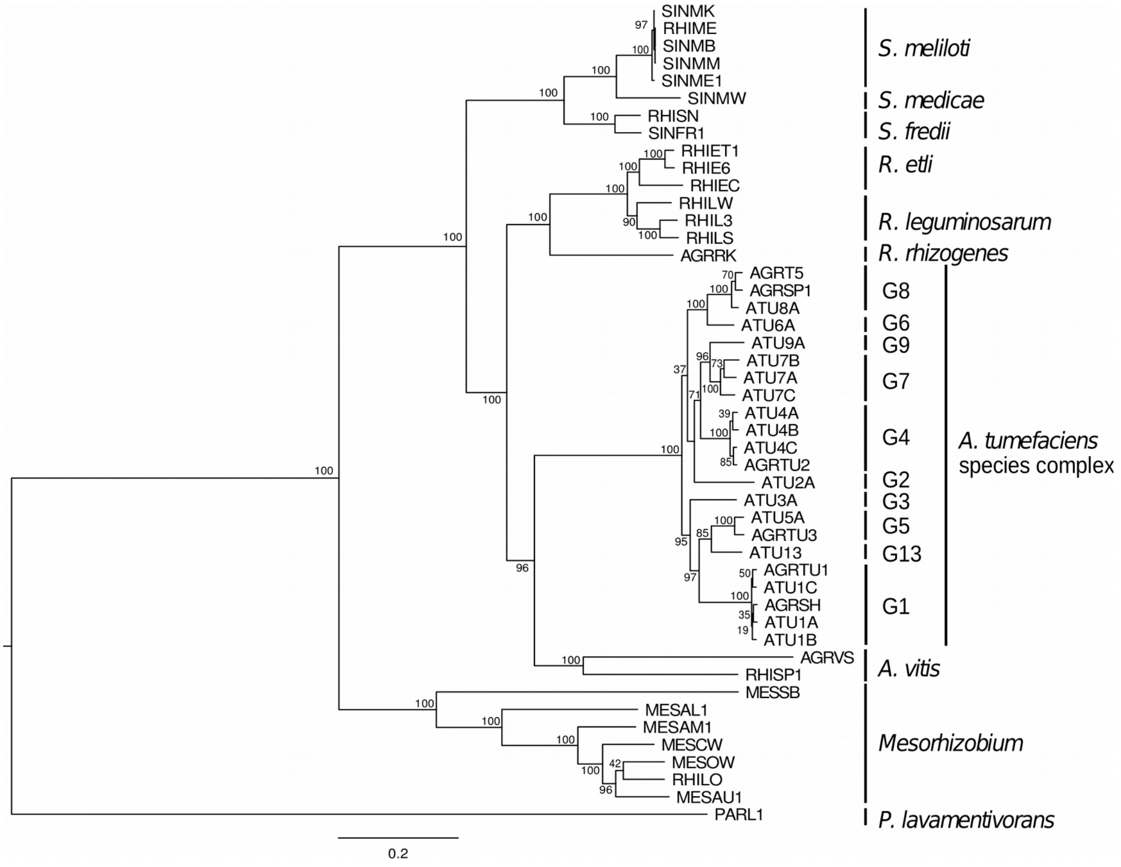

Fig. S2: Rereference phylogeny of Rhizobiales history.
Obtained by consensus of ML trees built from concatenates of 500 jacknife samplings of 25 genes among the 455 unicopy genes from the core of the 47 genomes. Supports of short branches within *S.meliloti* were all ≥ 0.9.

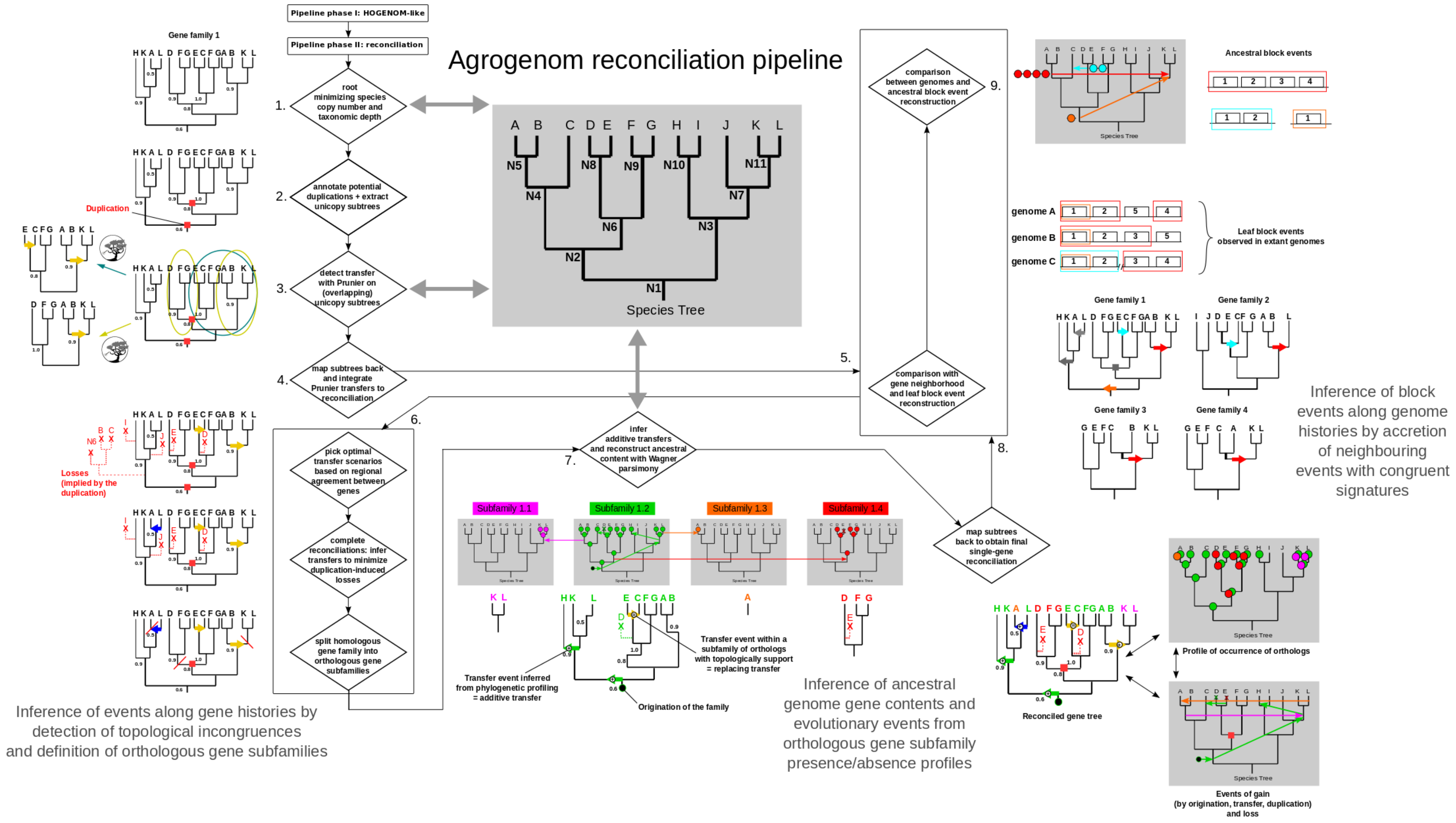

Fig. S3: Bioinformatic pipeline for reconciliation of gene and genome histories.
(1-6) Detection of horizontal transfer events; (7) Inference of gene content of ancestral genomes and integration of reconciliations with evolutionary scenarios; (8,9) Reconstruction of blocks of co-evolved genes.

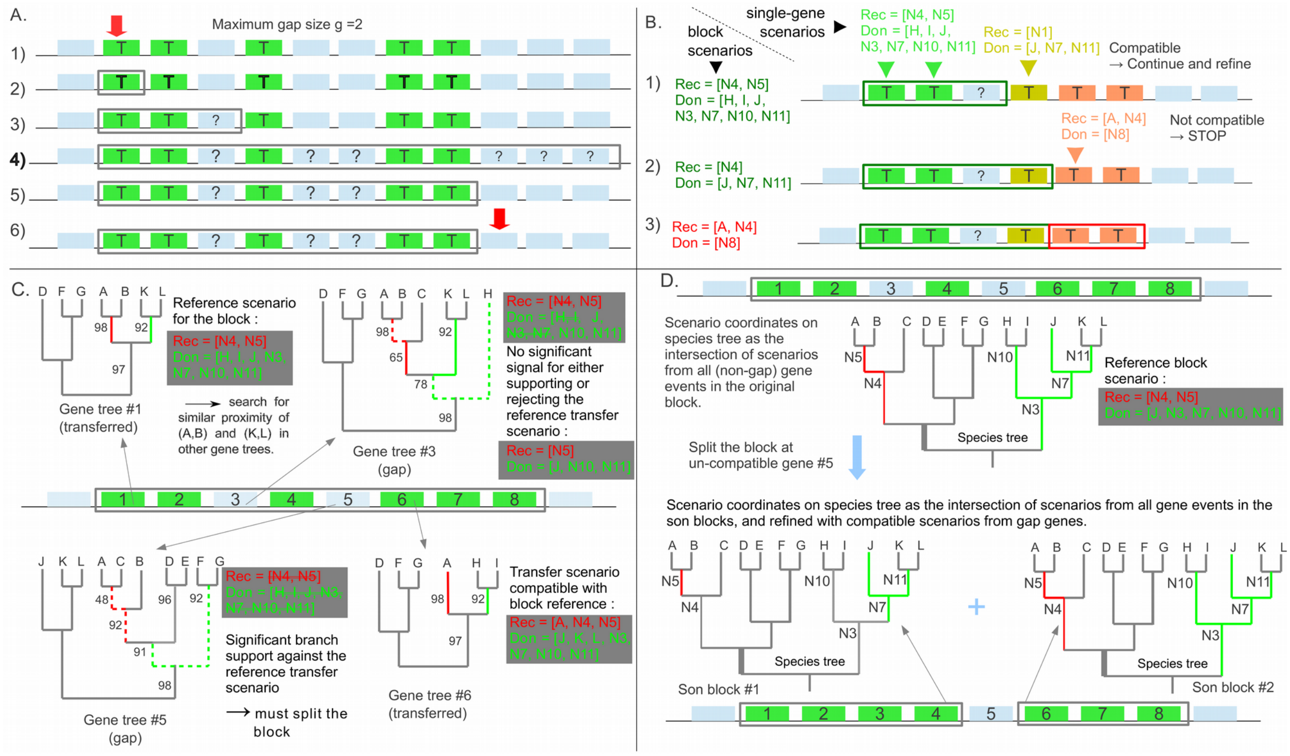

Fig. S4: Algorithm for construction of blocks of co-transferred genes.

A. Course of the extension of a block. A gene detected transferred (green 'T' boxes) is encountered during the course of the replicon (1). The block is initiated (2) and extended to gene having a consistent signal transfer (other 'T' boxes) or no signal of transfer (boxes '?'), In which case a gap is opened (3). When the extension leads to create a gap greater than size $g = 2$, the block is stopped (4), then extremal genes without signal for a transfer are removed from the block (5). The search for block events resumes after the last gene block (6).
B. Concordant or discordant scenarios of transfer. A block of events has an overall scenario corresponding to the intersection of those of its constituent genes. The scenarios are described as sets of possible receiver (Rec) and donor (Don) nodes, as exemplified Figure 2. The dark green block consists of genes whose transfer scenarios are identical (light green boxes, light green scenario) and gap genes without signal: the block has an overall scenario identical to light green genes (1). The block is extended to the brown gene for which the sets of possible receivers and donors are smaller: the global scenario sees its coordinates refined accordingly (2). When continuing the march on the chromosome to extend the dark green block, a pink gene is met; the donor set of the dark green block and that of the pink gene have an empty intersection: the scenarios are considered discordant, and the block is stopped (2); a new block is initiated (3). C-D. Checking the compatibility of 'gap' genes with the scenario of the block. C. Test if the scenario of the transferred genes (green boxes) is rejected by the 'gap' genes (blue boxes). D. Breaking of an inconsistent block into several blocks and re-computation of their respective scenarios.

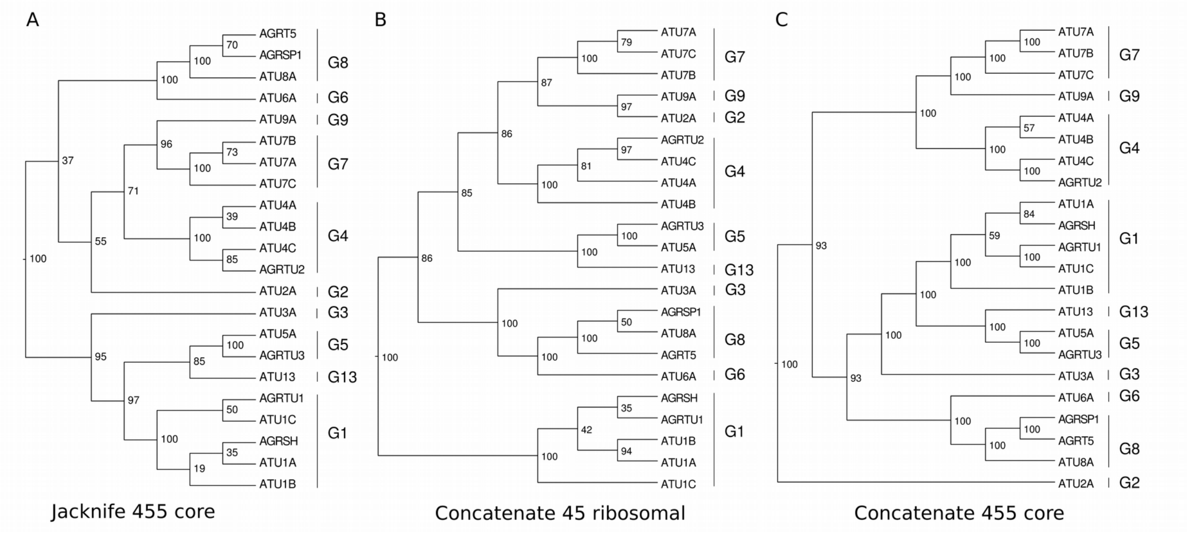

Fig. S5: Alternative reference tree topologies obtained with different methods:

(A) Consensus of ML trees obtained from concatenated alignments of jacknife gene samples (500 draws of 25 genes among 455 core unicopy genes), branch supports are based on their frequency in the 500 jacknife trees; (B) ML tree obtained from concatenated alignments of ribosomal protein genes, branch supports are based on 1,000 random bootstrap replicates ; (C) ML tree obtained from concatenated alignments of 455 core unicopy genes, branch supports are based on their frequency in 200 bootstrap replicates.

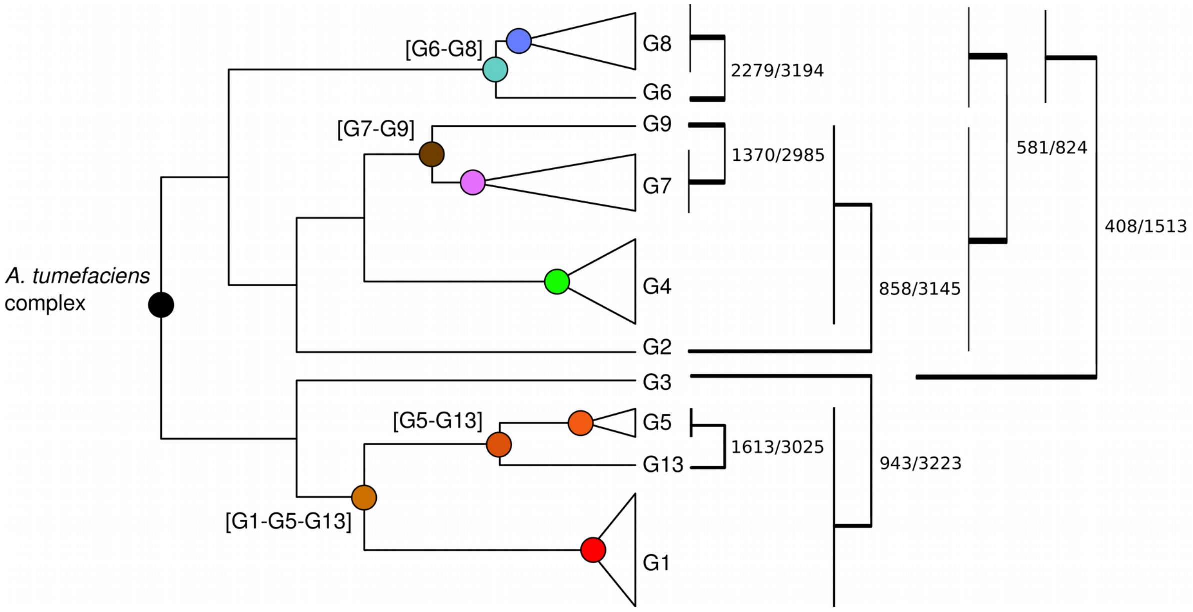

Fig. S6: Support for monophyly of groups in all gene trees.

Support for grouping of species into different clades, including some that are absent in the reference phylogeny (left), was tested in every gene tree. Tested groups are shown as pairs of clades in the reference tree, indicated by brackets on the right. Fractions indicate the count of gene families in the Agrogenom database that matched a pattern of monophyly over the number of families for which the monophyly could be tested (i.e. given the set of represented species covers representatives of the clades).

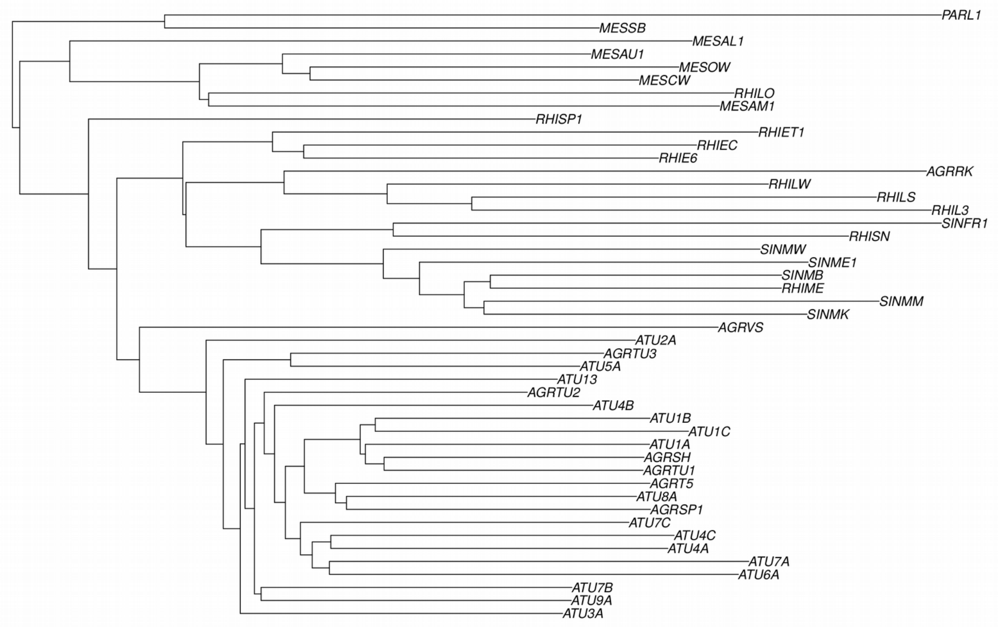

Fig. S7: Hierarchical clustering of *Rhizobiales* genomes according to their gene content. Hierarchical clustering was performed with the Neighbour Joining algorithm on euclidean distances computed from the occurence profiles of 41,664 gene families (possibly several counts) in extant genomes. Computations and plots were made using R package 'ape' (Popescu et al. 2012).

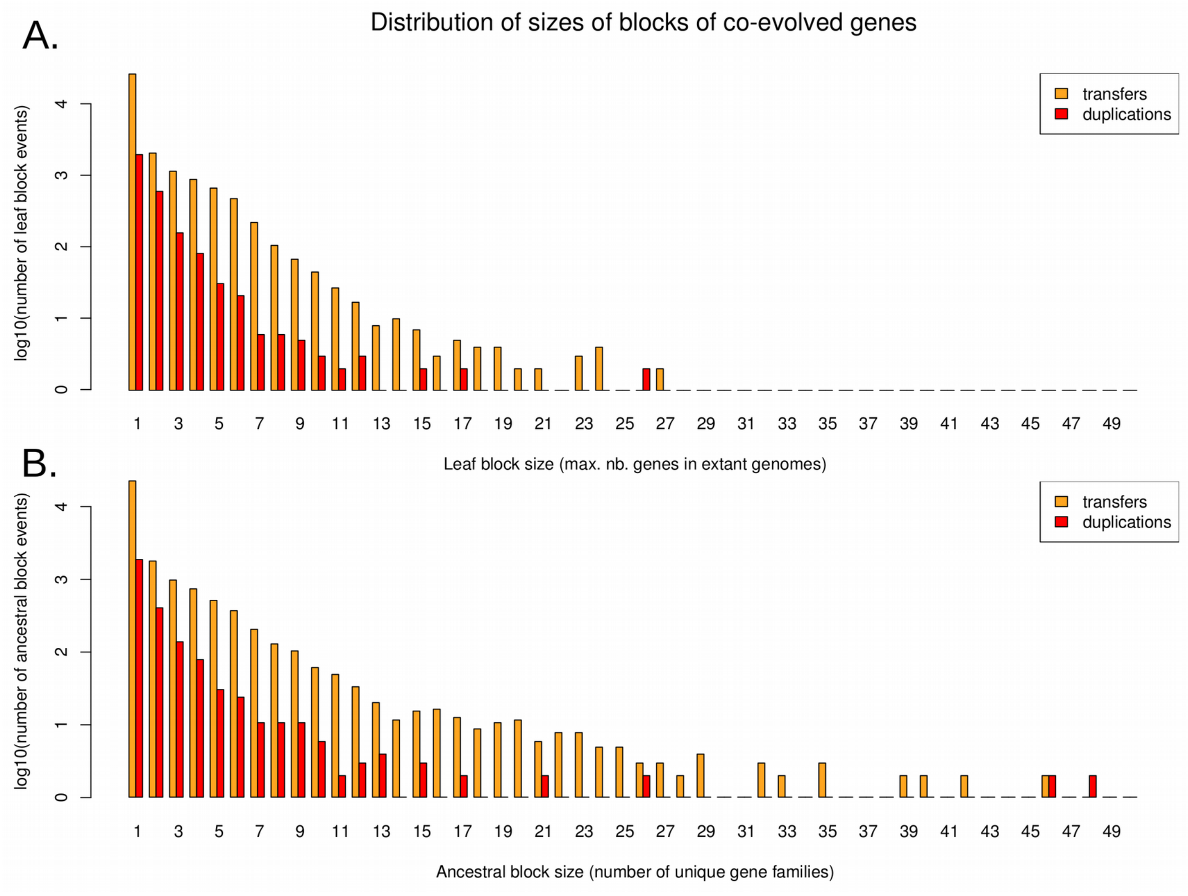

Fig. S8: Distribution of sizes of block events.

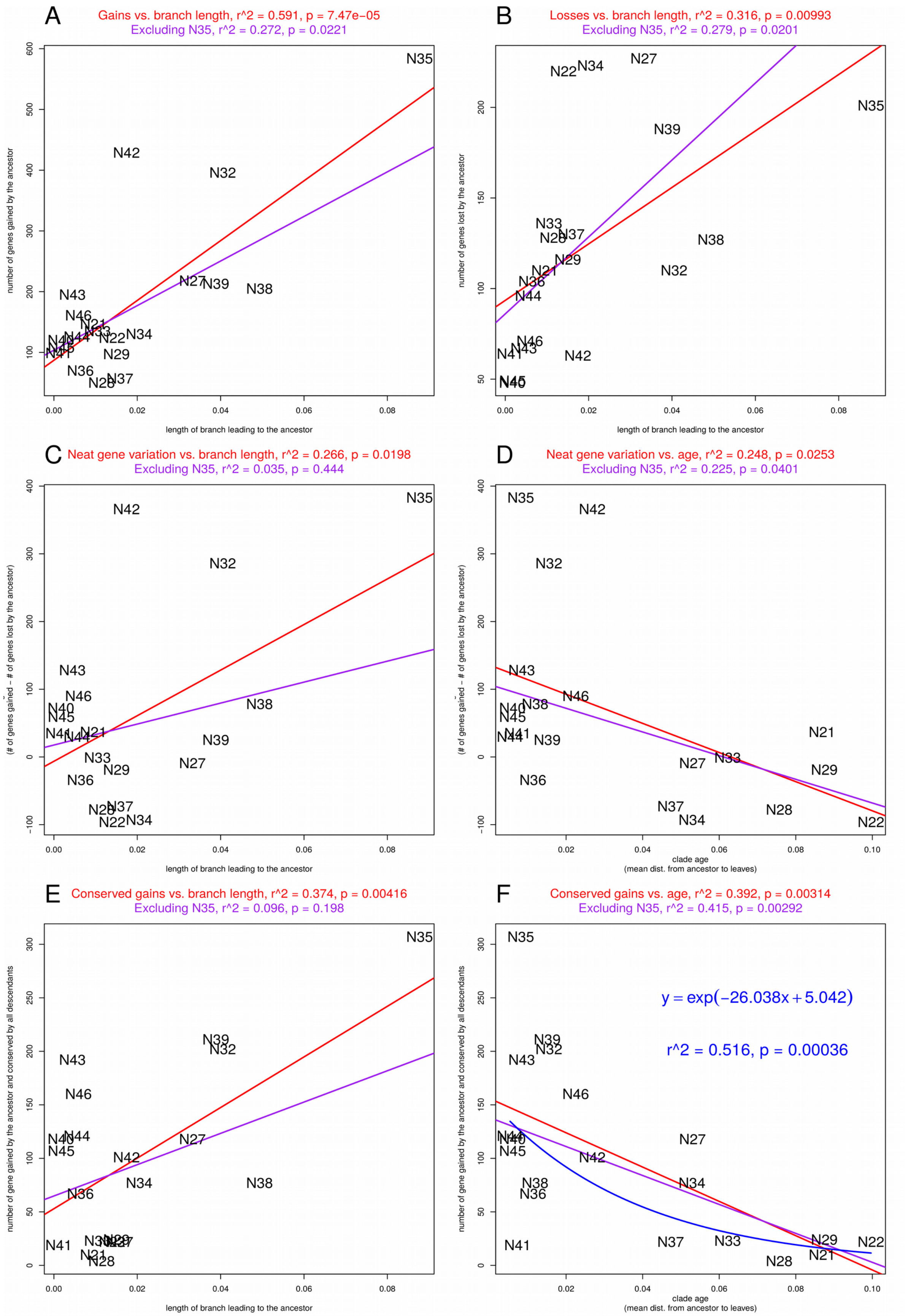

Fig. S9: Gene gain, loss and conservation within *At* clade ancestors.

Node numbers as in Figure 1 and Table S1.

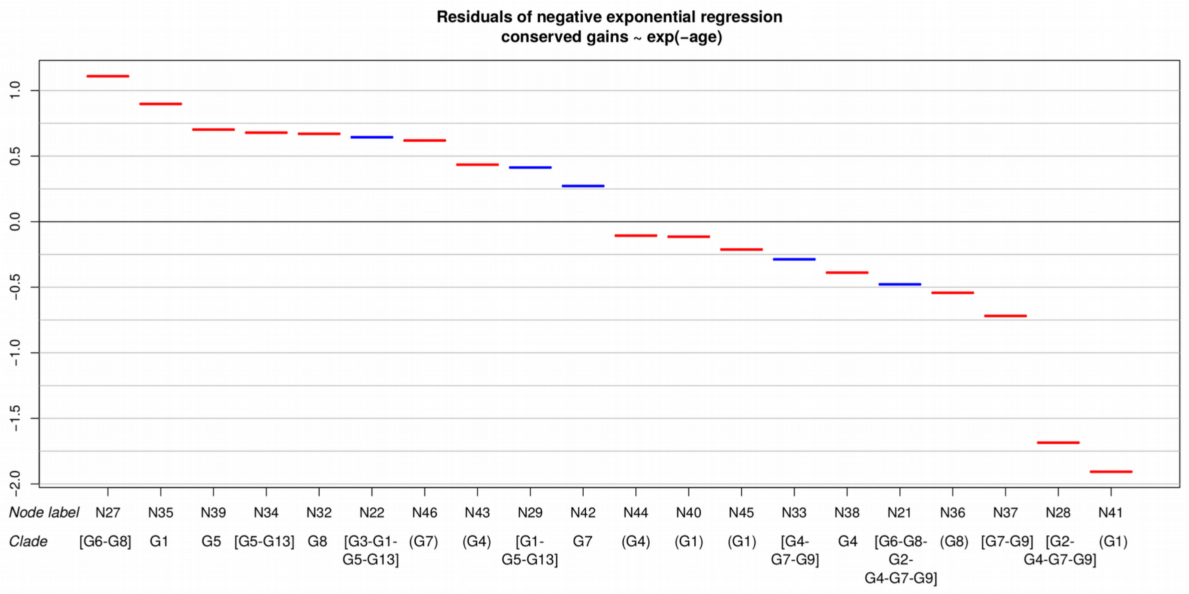

Fig. S10: Residuals of negative exponential regression of clade age vs. conservation of gained genes. Node numbers as in Figure 1. The regression is define as:

$$cg \sim \exp(-26.038 \cdot age + 5.042)$$

with *cg* the conserved gains. The regression has the following summary statistics:

$$\mathrm{Cov}(\log(cg), age) = -0.0269;\ \mathrm{sd}(age) = 0.0321;\ \mathrm{sd}(\log(cg)) = 1.165;\ r^2 = 0516$$

Residues in red fall outside of the 95% confidence interval of the regression. Clade names are indicated below corresponding species tree node labels when above the species level, or otherwise indicate in parenthesis the name of the species including the clade.

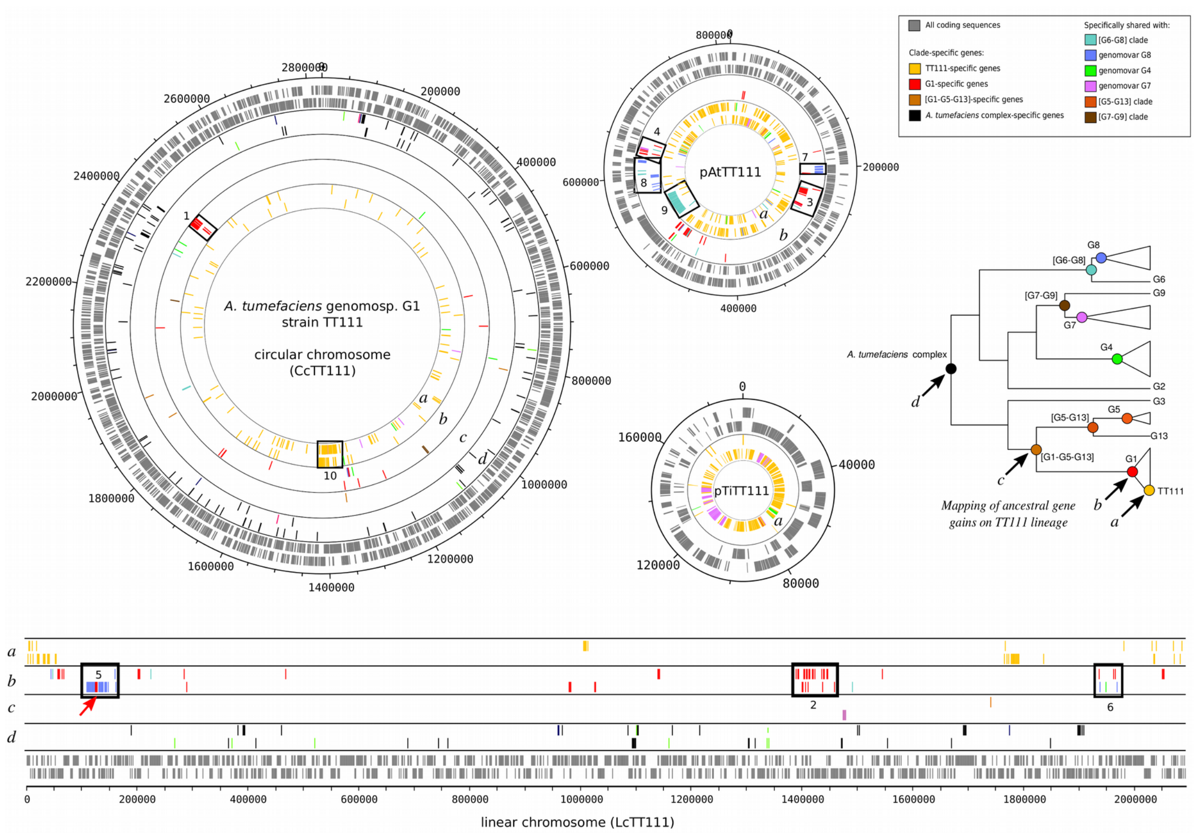

Fig. S11: Historical stratification of gains in the lineage of the *Agrobacterium sp.* G1 strain TT111. The four replicons of the genome are represented circularly or linearly according to their molecular topology; replicons are not drawn to scale. Tracks within outermost ring (lowermost layer for linear chromosome) represent location of CDSs on both DNA strands. Other rings (layers) show genes that were acquired along the history of the TT111 lineage, and are labeled (*a*-*d*) according to the species phylogeny in the right inset.. Colors of genes indicate their specific presence in one of the clade that includes TT111, or their common specific sharing with another clade (see legend box). Outer (lower) vs. inner (upper) tracks in the same rings (layers) distinguish clade-specific genes with strict vs. relaxed specificity criterion. Numbered frames show particular gene clusters within TT111 genome: (1-4) G1-specific clusters: (1) AtSp2: chemotaxis regulation (*che2*) and aromatic compound metabolism locus; (2) AtSp3: phenolics and amino-acid catabolism; (3,4) AtSp7 and AtSp9: phenolic compounds downstream degradation; (5-8), clusters specifically shared by G1 and G8: (5) AtSp14: lipopolysaccharide O-antigen biosynthesis and neoglucogenesis locus with G1-specific chemotaxis-regulating hybrid sensor (red arrow; red gene in Fig. S10); (6) AtSp12: outer-membrane lipoprotein and sensory protein; (7) AtSp17: deoxyribose uptake and assimilation; (8) AtSp15: exopolysaccharide (curdlan) synthesis, peptidoglycan modification and sensory protein; (9-10): clusters gained by TT111: (9) AtSp29: non-ribosomal peptide synthases involved in siderophore biosynthesis, shared by [G8-G6]; (10) prophage, partially shared by G3-CFBP6623 and G7-Zutra 3/1 (see Fig. S14).

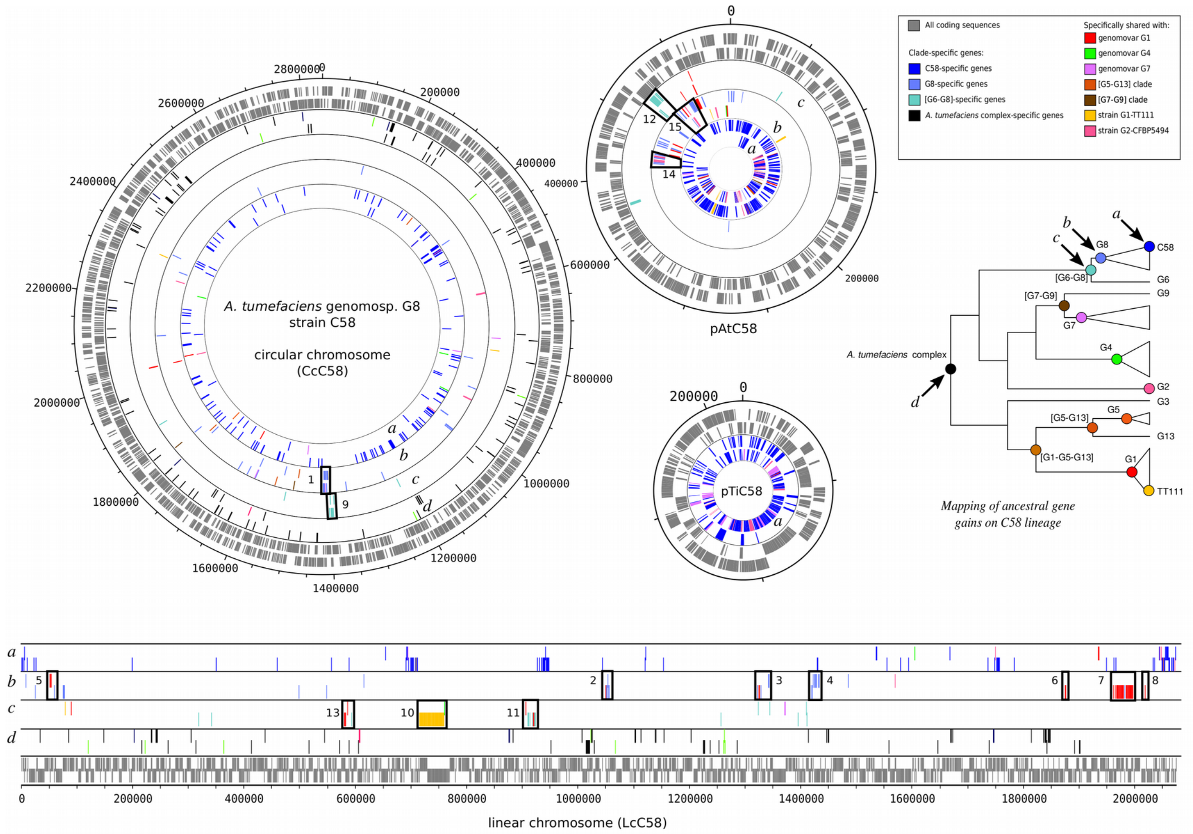

Fig. S12: Historical stratification of gains in the lineage of *Agrobacterium sp.* G8 (*A. fabrum*) strain C58. Legend as in Fig. S11. Numbered frames show particular gene clusters within C58 genome: (1-4) G8-specific gene clusters: (1) Atsp21: degradation of hydroxy-cinamic acids (ferulic acid); (2) AtSp23: degradation of complex amino-acids (opine-like compounds); (3) AtSp24 and AtSp25: Drug/toxic resistance (extrusion transporters), sarcosine oxidase; (4) AtSp26: sensing of environmental signals (phenolic compound, mechanical constrains); (5-8) clusters specifically shared by G1 and G8: (5) AtSp15: exopolysaccharide (curdlan) synthesis, peptidoglycan modification and sensory protein; (6) AtSp13: iron-sensing two component system FeuPQ; (7) AtSp14: lipopolysaccharide O-antigen biosynthesis; (8) AtSp12: outer-membrane lipoprotein and sensory protein; (9-12) [G6-G8]-specific gene clusters: (9) AtSp29: sugar (L-sorbose) uptake and catabolism; (10) AtSp30: non-ribosomal peptide synthases involved in siderophore biosynthesis, shared by G1-TT111 (see Fig. S11); (11) AtSp31: sugar metabolism; (12) dipeptide uptake and degradation; cluster specifically shared by G1 and [G6-G8]: (13) AtSp18: D-glucuronate uptake and degradation; (14-15) clusters specifically shared by G2 and G8: (14) AtSp27: Toxic extrusion / secondary metabolite secretion; (15) AtSp28: xanthine/cyclic compound degradation, two-component sensor.

Fig.

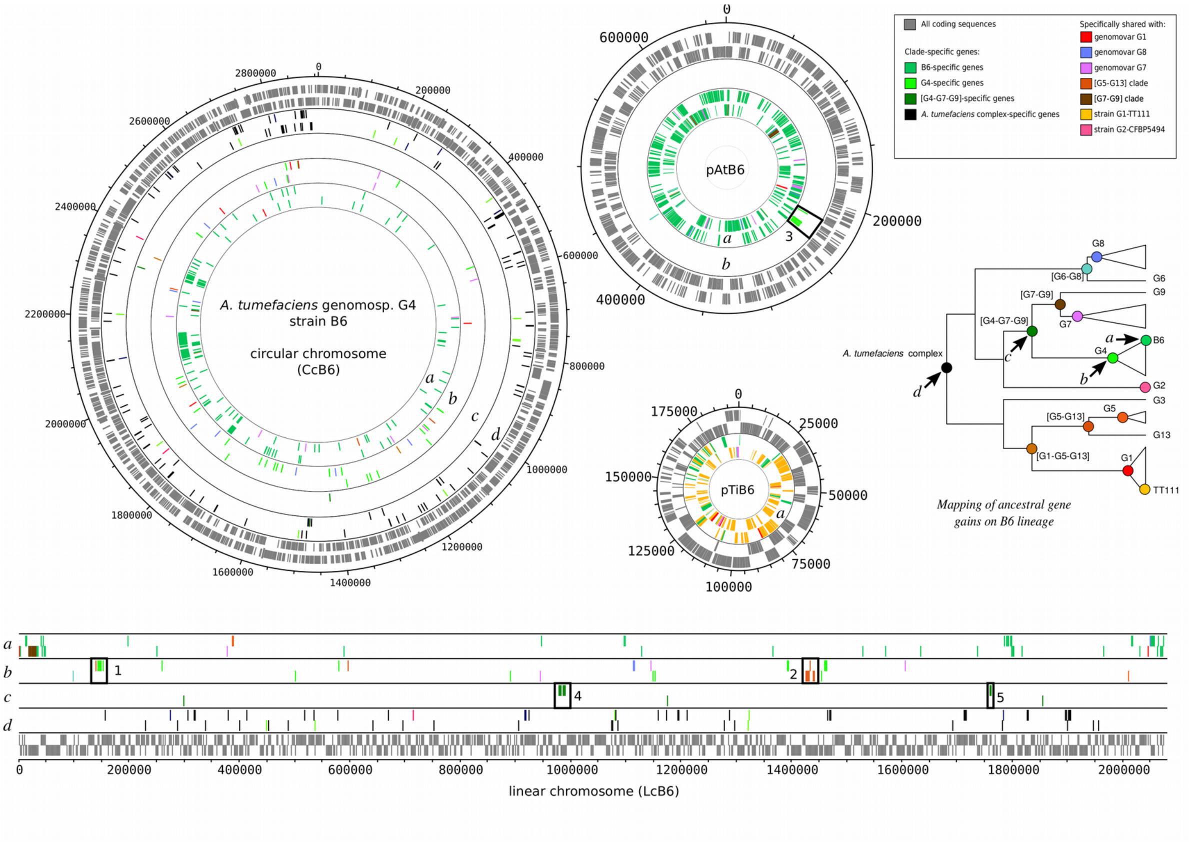

S13: Historical stratification of gains in the lineage of *A. sp* G4 (*A. radiobacter*) strain B6.

Legend as in Fig. S11. Numbered frames show particular gene clusters within B6 genome: (1-3) G4-specific gene clusters: (1) Atsp37 : aromatic compound (acriflavine) perception, degradation and efflux; (2) Atsp38 : uptake and catabolism of sugars (sorbose, dehydro-fructose) (shared by [G5-G13] clade); (3) Atsp39 : gamma-glutamyl cycle for detoxification of periplasmic compounds; (4-5) [G4-G7-G9]-specific gene clusters: (4) Atsp40 : uptake and degradation of a (sulfated) polygalacturonide/polyglucuronide; (5) Atsp41 : ferrichrome-iron sensing and uptake. Note that the major part of pTiB6 is shared by G1-TT111, as was previously shown (Lassalle et al., 2011), suggesting that pTiB6 and pTiTT111 are related by a recent transfer event.

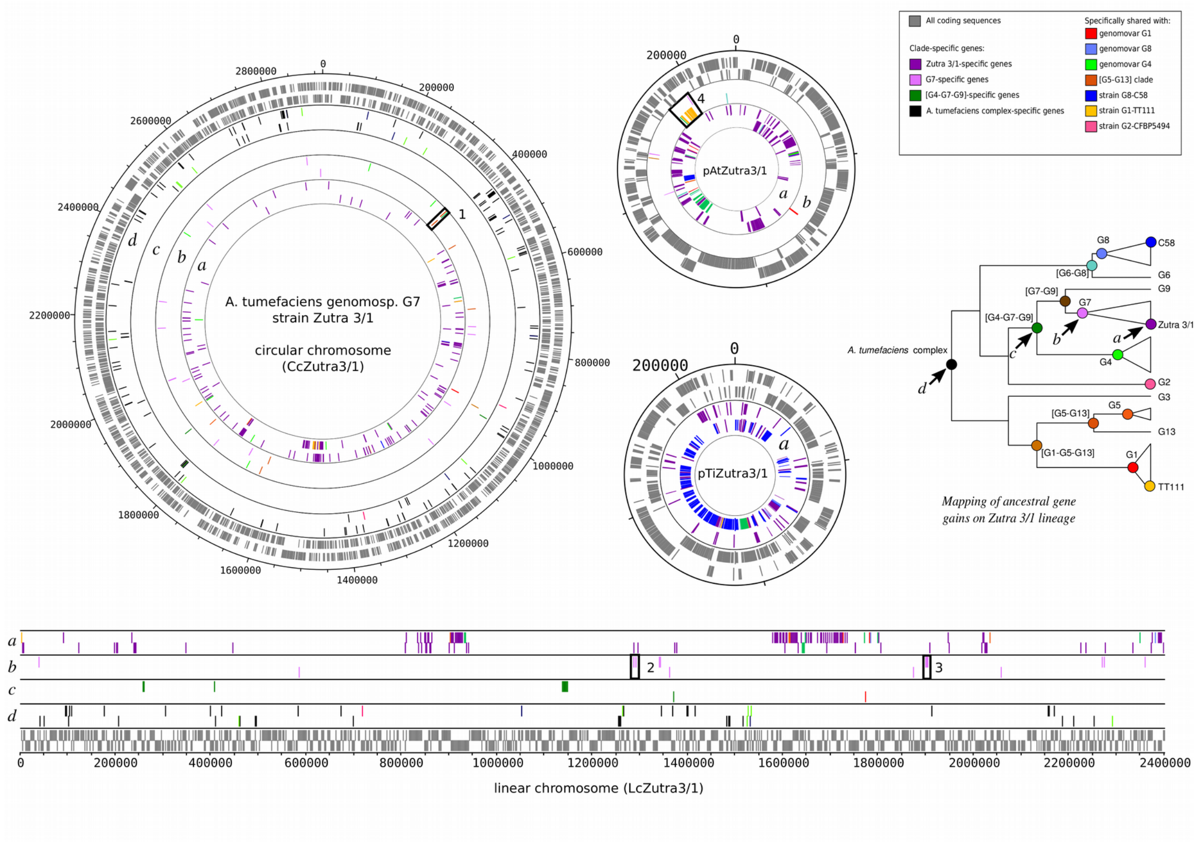

Fig. S14: Historical stratification of gains in the lineage of *A. sp. G7* strain Zutra 3/1.

Legend as in Fig. S11. Numbered frames show particular gene clusters within Zutra 3/1 genome: (1-4) G7-specific gene clusters: (1) Atsp42: Quorum sensing-regulated periplasmic protein-disulfide isomerase DsbABG (ensures proper conformation of disulfide-bonded proteins like c-type cytochromes for covalent haem attachment); (2) AtSp46: prohibitin (DNA synthesis represession), phage shock protein (represses the expression of sigma54-dependent operons); (3) AtSp47: system for sensing (FecIR), TonB-dependent import (FhuACD) and utilization (ViuB) of iron(3+)-hydroxamate siderophore; (4) AtSp48: enzymes and regulators of unknown function.

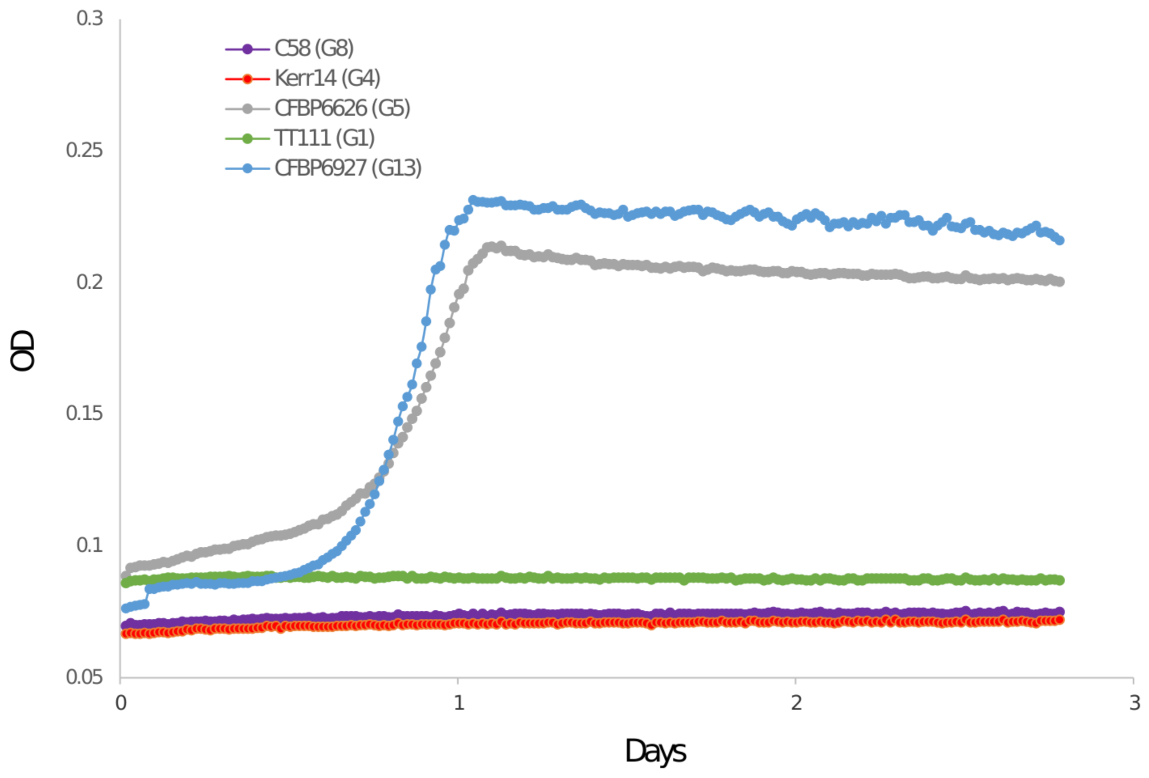

Fig. S15: Growth curves of representative of *At* genomic species on phenylacetate

Agrobacterium strains grown overnight in AT medium supplemented with succinate and ammonium sulfate were inoculated at an optical density at 600 nm (OD_{600}) of 0.05 in 200 µl AT medium supplemented with appropriate carbon and nitrogen sources.

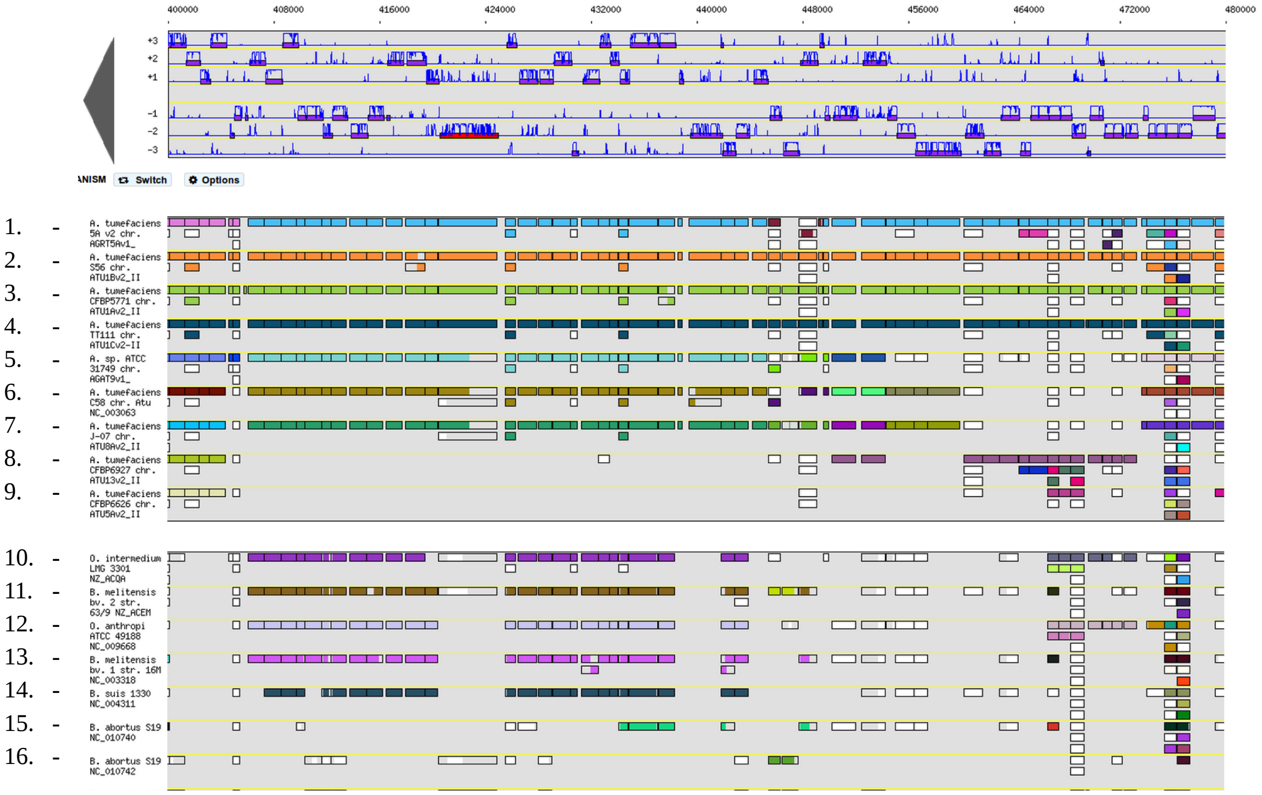

Fig. S16: Syntenic conservation of the AtSp14 cluster in G1, G8 and Brucelaceae.

Microscope MAGE's view of the locus in G1-H13-3 linear chromosome (top), with a projection of syntenic genes found in compared genomes (bottom), including (from top to bottom) genomes of G1 (rows 1-4), G8 (rows 5-7), G13 (row 8) and G5 (row 9), as well as genomes of selected Brucellaceae (rows 10-16). These genes code the biosynthesis of an O-antigen decoration of the lipopolysaccharide (LPS); HHSS gene is coloured in red. View accessible at https://www.genoscope.cns.fr/agc/microscope/mage/viewer.php? when selecting *Agrobacterium sp.* H13-3 chromosome CP002248 at coordinates 400,000 to 480,000 as a reference.
