# Supplementary material for: Ancestral Genome Estimation Reveals the History of Ecological Diversification in Agrobacterium
Source: Genome Biol Evol. 2017 Dec 6;9(12):3413–31. doi: 10.1093/gbe/evx255 (PMC5739047; doi:10.1093/gbe/evx255)
Supplement: Supplementary Materials [file reviews_round2_3.pdf › reviews_round2_3.pdf]

22-Sep-2017

Dear Dr. Lassalle,

Your manuscript GBE-161023.R1 entitled "Ancestral genome reconstruction reveals the history of ecological diversification in Agrobacterium." which you submitted to Genome Biology and Evolution, has now been reviewed. The comments of Prof. David Bryant, the Associate Editor who handled this peer review, and the comments of the referees are included at the bottom of this letter.

You will be pleased to see that the evaluation of the paper is generally quite positive, but some minor revisions will need to be undertaken before your manuscript can be accepted for publication. Therefore, I invite you to respond to the referees' comments and revise your manuscript. Please ensure that you include both, a Word document of the manuscript text and tables, and a pdf file of the whole paper, including all tables and figures but without tracking or supplementary material, as well as the usual files.

To revise your manuscript, log into https://mc.manuscriptcentral.com/gbe and enter your Author Centre, where you will find your manuscript title listed under "Manuscripts with Decisions." Under "Actions," click on "Create a Revision." Your manuscript number has been appended to denote a revision.

You will be unable to make your revisions on the originally submitted version of the manuscript. Instead, revise your manuscript using a word processing program and save it on your computer. Please also highlight the changes to your manuscript within the document by using the track changes mode in MS Word or by using bold or coloured text and either submit both versions (if there are many changes) or just a tracked version. The previous version of your paper will be deleted.

Once the revised manuscript is prepared, you can upload it and submit it through your Author Centre.

When submitting your revised manuscript, please respond to the comments made by the referees in the space provided, documenting also changes you make to the original manuscript. The responses will be available to reviewers should there be a further review.

IMPORTANT: Your original files are available to you when you upload your revised manuscript. Please delete any redundant files before completing the submission.

Because we are trying to facilitate timely publication of manuscripts submitted to Genome Biology and Evolution, your revised manuscript should be uploaded as soon as possible. If it is not possible for you to submit your revision within two months, we may have to consider your paper as a new submission.

Once again, thank you for submitting your manuscript to Genome Biology and Evolution and I look forward to receiving your revision.

Yours sincerely,
Bill Martin
Editor in Chief, Genome Biology and Evolution

w.martin@uni-duesseldorf.de

Associate Editor Recommendation:

Associate Editor: Bryant, David
Comments to the Author:
Thank you for submitting this revised version of your manuscript. I think it is a pity that you did not take the opportunity to significantly simplify and streamline the paper, however I acknowledge the difficulty of that task.

I was unable to find reviewers who were willing and able to take on a manuscript like this, so have ended producing the required review myself. That too has proven difficult, mainly because of the impossibility of finding those 2-3 hour blocks needed to carry out this kind of work, during the semester at a teaching university.

So I apologise for the delay and accept full responsibility. In the end, though,
I would very much like to see the manuscript published in GBE. I think that the only step remaining is to work on matters of presentation, making sure that your valuable contributions are not hidden by suboptimal structure or presentation.

>>> Dear GBE Editor,

we are really grateful for your personal commitment in reviewing our manuscript. We appreciate the scarcity of free time for a senior academic and must therefore thank you for your dedication.

Many relevant points have been raised, notably the need to improve the readability of the paper so that this study can reach a wider audience in the scientific community and make a significant impact.
We acknowledged that improvements were still possible and therefore did our best to make this revision a clearer support of the scientific message.

This notably included clarifying the procedure used for the block event estimation, for which a more step-by-step description (main doc., Methods section) and another version of pseudo-code (Sup. Text) are provided.
We also benefited from the service of a professional proofreader, who made necessary corrections of the English grammar and suggested many changes to improve sentence structures.
While some sections needed clarification and therefore additions of text, the text has been globally streamlined, with shorter sentences and less repetitions; this is also true of the Sup. Text, where we notably dropped an entire section (former section 4 for super-detailed description of the reconciliation procedure, now more adequately described online on the github repository).

Altogether, we have the (pleasant!) impression that the manuscript really does read better. We hope that we addressed all your other comments correctly and that that the present manuscript will finally be fit for publication in Genome Biology and Evolution.

With best regards,

Florent Lassalle, on behalf of all co-authors.

In addition to the many specific comments below, I'd like to make two general observations.

(1) This is a long manuscript, and there are still a large number of grammatical errors or, if not errors, text which could be improved. I have picked up some, but that is not my strength and personally I use a professional proof reader for my own work. My feeling it that this manuscript could benefit from that.

>>> A correction of the English language has been conducted by a professional scientific proof reader.

(2) Be very careful to distinguish between things which you know and things which you estimate. For example, we can't reconstruct evolutionary history - we estimate it.

>>> We made an extensive effort to avoid the verb 'reconstruct' throughout the ms, replacing it by 'estimated' or 'inferred'.
We preferred the latter word for the often mentioned "block event reconstruction" → "block event inference", as well as in the title: "Ancestral genome inference reveals the history of ecological diversification in *Agrobacterium*."
In places, to diversify the lexicon, we also used (when adequate): 'retrace', 'deduce'.
The title now reads: "Ancestral genome estimation reveals the history of ecological diversification in Agrobacterium."

As an example, you have an approach for detecting selection: at the start of manuscript the patterns 'suggest' purifying selection; by pg 13 the patterns are indicative of purifying selection; by the conclusion, signatures of purifying selection are being revealed.

>>> The use of 'suggestive' vs. 'indicative' vs. more affirmative wordings in conjunction with claims of occurrence of natural selection was checked throughout the manuscript and corrected; in particular the three sentences mentioned above (all relating to the excess of gene gains in certain ancestral *At* lineages *suggesting* purifying selection) were consistently corrected to use the 'suggest' wording.

In the following, line numbers are the ones which go from 1 to 800+

45 "Another approach". Its not clear to me what the first approach is. Perhaps spell this out.
>>> It was rather the only approach we could name; "In order to discover those functions that are ecologically relevant, an approach..." was added to the beginning of the sentence.

40 - 65 The first paragraph needs to be broken into smaller paragraphs covering the same topic. It could benefit from some reordering too.
>>> The paragraph was broken down into 1) the idea of seeking genomic traces of selection to understand bacterial ecology, 2) what kind of null model of evolution we rely on, i.e. a pangenome with fast turnover, 3) what sort of departure from this model we are looking for, i.e. the occurrence of domesticated, clade-specific genes.

73. were in majority clustered -> were usually clustered >>> corrected

99 Our estimated pan genome history >>> corrected

164 and the root consistent with the most parsimonious >>> corrected

167 assigning an event -> assigning a classification?

>>> We reworded this sentence to “We reconciled the gene tree topologies with the species tree by annotating each of the 467,528 nodes found in the 10,774 gene trees with an estimated event of origination, duplication, transfer (ODT), or speciation”

171 Shortly, gene trees -> In brief, gene trees >>> corrected

175 In presence -> To deal with >>> corrected

181 yielded an estimate of 22,322

>>> We reworded this sentence to “These reconciliations of potentially overlapping local subtrees yielded point estimate scenarios (involving a total of 22,322 phylogenetically supported transfer events), which were mapped back to the gene trees”

185 - 190. This raises a red flag. If supports were too low for Prunier, could this be due to sampling error? Have you discounted this possibility?

>>> (I will assume that this comment refers to a poor sampling of the reconciliation space by Prunier, not of topologies by the gene tree inference tool [here PhyML]; however this latter case is also accounted for, see below.)

This case cannot be due (in general) to a poor sampling by Prunier’s heuristic of the reconciliation space, because no transfer scenario would be invoked anyway if the putative transfer recipient was separated in the gene tree from cognate species (those leaves representing the recipient’s sister group in the species tree) only by branches with support lower than the threshold (0.9).

However weakly supported it is, the (point estimate) topology of the gene tree can remain in conflict with the species tree. These incongruences can be explained either by duplication(s) (and losses) or transfer(s). At this step 6 is evaluated what is more parsimonious between these two possible events in terms of induced losses, and the best is retained.

Another option would be to consider the tree topology wrong (as indeed it is not supported) and try to change it so that it induces less conflict. This is however exactly what is already done at step 2, where SPR moves (that don’t disrupt well supported branches) are attempted on nodes marked as potential duplications. This part of the reconciliation pipeline was not clearly mentioned in the main text (only in the sup methods and on the github page), so a sentence has been added to the paragraph (L178) and to the inset caption for step 2 in Figure S3.

Step 6 is therefore only intended to fully reconcile the gene and tree topology (notwithstanding how supported it is) with the species tree,

something that Prunier does not (implicitly considering that a topology rearrangement could be done on weakly supported branches to reach full agreement with the species tree). This is made clearer in this sentence (L196-200).

206 events that involved several genes >>> corrected

210 The right count and coordinates -> Counts and locations >>> corrected

212 - 215. I couldn’t understand what you meant here. Please restructure, rewrite and clarify.

>>> The sentence was broken down and clarified, it now reads:
“To get a point estimate of a scenario with gains and losses, one typically applies a criterion of parsimony on the count of loss events subsequent to a gain (e.g. by transfer), so the gain event is estimated to be located at the last common ancestor of the species represented in the recipient clade. Given that gene families often have different species representations, this can result in family-specific systematic biases when estimating event coordinates. Unmatched biases in coordinate estimates would strongly affect our ability to recognize that a same co-event affected neighbour gene families (Fig. 1 A).”

220 - 270. This bit of methodology is absolutely core to your paper, and yet I was not able to figure out exactly what you were doing (let alone be able to implement it)
I suggest that, here, you make a high level reference to a greedy algorithm. Then in the supplementary material you give actual pseudo-code - at least something that a programmer might have a fighting chance to implement (and a statistician might have a fighting chance to understand and critique)

>>> We understand the description of this procedure still lacked clarity while being at the centre of the method we describe. We thus made an effort to extensively reword and clarify the block construction algorithm in the Methods section and to provide a leaner and clearer version of the pseudo-code description of algorithms in the Sup. Text section 4.

The former version of the pseudo-code had a higher degree of detail, with more notations introduced (to the point that, indeed, it was closer to actual code than a description, and was not extremely accessible). It however responded to the request of reviewer 1 on the first round of review (who seemed satisfied of it); for this reason, this more detailed version of the algorithms was kept as an Annex and is now to be read online at:
https://github.com/flass/agrogenom/tree/master/doc/

232. Fix tense.

>>> Tense within this paragraph was harmonized to set the description in the past. This was generally applied to the manuscript to describe how we conducted the investigation.

244. Ancestral block events were then estimated >>> corrected

273 Contrast between what?

>>> The following sentence was reworded to better explain that the contrast is established between a background clade and a nested foreground clade.

350 Infer the history -> Estimate the history >>> corrected

352 “which combination as a” what do you mean here?

>>> We reworded these sentences as: “we reconciled the topologies of gene trees and species tree, i.e. we explained their incongruence by assigning events of either origination, duplication, transfer (ODT), or speciation to the gene tree nodes. […] The combination of events estimated in each gene tree resulted in an estimated scenario of evolution of the gene family along the species tree.”

369 Yet, to date, ODT scenarios have generally been evaluated >>> corrected

374 inferred putative block events

>>> corrected to “we inferred putative ‘block events’, i.e. unique evolutionary events that involved blocks of neighbour genes”

385 give percent loss too.

>>> We modified this sentence to “[…] resulted in the decrease of 13,421 ODT events, most of them transfer (10,978 T events, -25.4%) and an increase of loss (L) events (2,896, +9.7%)”

390. “This difference in the estimated number of gene losses is due to the frequent under-estimation… ” Sorry - I don’t see why this is the case. Please give a bit more explanation here.

>>> To justify this statement, we added the following sentences: “Indeed, the loss scenarios were generally estimated by fixing the timing of the preceding gene gain (O, D or T) events to their most recent possible location – the most parsimonious solution with respect to losses. In the case of block event scenarios, ODT events were dated to the most recent *common* location of all single-gene event parts, which by definition must be equally ancient as, or more ancient than the single-gene estimates. This resulted in globally older ancestor for block gain events, with a higher number of lineages between the ancestor and extant representatives in which to invoke subsequent losses”

405 indicating either a potential artefact or….

>>> We have no reason to think this is an artefact; rather, we reworded and expanded the sentence such as: “[…] indicating a divide in contemporary genomes between a long-standing gene repertoire and a large fraction of newly acquired genes still segregating in the population. Our ancestral genome estimation procedure did not estimate the count of unobserved ancient genes; however, a similar-size polymorphic gene repertoire probably existed in the At clade ancestors and was mostly lost in all sampled descendants.”

407 evolutionary distances between what? taxa?

>>> the expression ”evolutionary distance on the species tree” intended to cover the different branch length-based metrics discussed below, i.e. the length of the branch leading to the ancestor and the age of the ancestor (cumulative mean branch length of the node to its tips). This sentence was however of little use and was therefore removed.

445 Hence the best >>> corrected

446. Please explain why

>>> The argument is that traits coded by several independent evolving units such as genes, and not relying on a common genetic background are by combinatorial rule less likely to occur convergently in several individuals, which should experience the same set of mutations. This was emphasized in the phrasing:
“Hence, the best candidates for niche-specifying traits consist of novel and complex traits relying on an array of biochemical functions coded by separate evolving units (genes) and do not depend on pre-existing pathways, making such mutations unlikely to occur several times by chance”
A counter argument would be that genes participating in a same complex trait are not independent evolving units, as they occur in close linkage in genomes (operons) and are therefore more likely to be co-transferred and thus co-acquired. This is exactly what we study below, evaluating if such putatively rare arrays of co-functioning genes are being selected while accounting for the linear structure of bacterial genomes.

462 - 475.. Much of the description of this needs to be moved to the methods section.

>>> We agree, parts of the first paragraph ”To test whether […]” were redundant with the Methods and could simply be removed.

519. Is this section really relevant to the main contributions of the paper?

>>> We would think so. Indeed, features of bacterial genomic architecture have previously been used to delineate taxa as they constitute obvious synapomorphies and possibly carry adaptive value, or confer differential sexual efficacy among bearers, properties that can contribute to (sympatric) speciation. In this particular case of the pAt replicons - that were previously called ‘cryptic’ and whose contribution to the bacterium’s physiology was discounted in most studies - we show that they are in fact bearer of core genetic traits at the species level, with a likely adaptive role in the species’ ecological niche. This should change the perception of a lot of *Agrobacterium* biologists for whom plasmids are interchangeable among strains and species – a fact that we show not to be true for the pAt, which indeed constitute a species marker. This was stressed by adding the sentence:
“Functional investigation of the core functions borne by agrobacterial pAts could thus provide a better understanding of the specific ecophysiology of each At species.”

519 Rhizobiazeae present -> Rhizobiazeae have >>> corrected

521 called the chromid >>> corrected

Reviewers' Comments:

Referee: 1

Comments to the Author
My comments have been addressed
